# Activation of PKC supports the anticancer activity of tigilanol tiglate and related epoxytiglianes

**DOI:** 10.1038/s41598-020-80397-9

**Published:** 2021-01-08

**Authors:** Jason K. Cullen, Glen M. Boyle, Pei-Yi Yap, Stefan Elmlinger, Jacinta L. Simmons, Natasa Broit, Jenny Johns, Blake Ferguson, Lidia A. Maslovskaya, Andrei I. Savchenko, Paul Malek Mirzayans, Achim Porzelle, Paul V. Bernhardt, Victoria A. Gordon, Paul W. Reddell, Alberto Pagani, Giovanni Appendino, Peter G. Parsons, Craig M. Williams

**Affiliations:** 1 Drug Discovery Group, QIMR Berghofer Medical Research Institute, Brisbane, QLD Australia; 2 School of Biomedical Sciences, Faculty of Health, Queensland University of Technology, Brisbane, QLD Australia; 3 School of Biomedical Sciences, Faculty of Medicine, University of Queensland, Brisbane, QLD Australia; 4 School of Chemistry and Molecular Biosciences, University of Queensland, Brisbane, QLD 4072 Australia; 5 QBiotics Group, Yungaburra, QLD Australia; 6 Dipartimento di Scienze del Farmaco, Università Degli Studi del Piemonte Orientale, Largo Donegani 2, 28100 Novara, Italy; 7 QIMR Berghofer Medical Research Institute, Royal Brisbane Hospital, Locked Bag 2000, Brisbane, QLD 4029 Australia

**Keywords:** Natural products, Natural products, Mechanism of action

## Abstract

The long-standing perception of Protein Kinase C (PKC) as a family of oncoproteins has increasingly been challenged by evidence that some PKC isoforms may act as tumor suppressors. To explore the hypothesis that activation, rather than inhibition, of these isoforms is critical for anticancer activity, we isolated and characterized a family of 16 novel phorboids closely-related to tigilanol tiglate (EBC-46), a PKC-activating epoxytigliane showing promising clinical safety and efficacy for intratumoral treatment of cancers. While alkyl branching features of the C12-ester influenced potency, the 6,7-epoxide structural motif and position was critical to PKC activation in vitro. A subset of the 6,7-epoxytiglianes were efficacious against established tumors in mice; which generally correlated with in vitro activation of PKC. Importantly, epoxytiglianes without evidence of PKC activation showed limited antitumor efficacy. Taken together, these findings provide a strong rationale to reassess the role of PKC isoforms in cancer, and suggest in some situations their activation can be a promising strategy for anticancer drug discovery.

## Introduction

The Protein Kinase C (PKC) family of serine/threonine kinases was first identified almost 40 years ago^1^. The family comprises nine genes encoding ten main PKC isoforms in humans, divided into three subgroups according to sequence homology and cofactor requirements^2^. The classical, or conventional, PKC subgroup requires binding of calcium and diacylglycerol (DAG) for activation and comprises isoforms PKCα, − βI and-βII (alternatively spliced from the same gene), and  − γ. Conversely, members of the novel PKC subgroup (− δ, − ε, − η and − θ isoforms) require only DAG for activation, while the atypical PKCs (− ι and − ζ isoforms) are activated independently of calcium and DAG, but depend on distinct lipid-based mediators and protein–protein interactions^3^.

PKC isoforms have long been considered oncoproteins, due to their role as kinases and their identification as the direct targets of “tumor promoting” phorbol esters. However, despite significant investment in clinical trials with a number of investigational drugs, pharmacological inhibition of PKC has been unsuccessful in treating solid tumors^4^. Many questions remain around the possible role of PKC in tumorigenesis, with a growing body of evidence now suggesting that certain PKC isoforms may actually act as tumor suppressors. For example, recent studies have found over 1,000 cancer-associated somatic mutations in PKC isoforms, mostly resulting in loss of function^5^, while in addition, many cancers are known to display down-regulated expression of PKC family members^6–10^. It has therefore been proposed that anticancer therapies should focus on inducing or restoring PKC activity rather than inhibiting it^11,12^.

One important implication of this hypothesis is that to produce the greatest therapeutic effect on cancer, re-instating or inducing prolonged activation of specific PKC isoforms would ideally occur without subsequent degradation or downregulation of the protein^11,12^. However, classical phorbol esters are known to rapidly activate PKC signaling and then induce proteolytic degradation of activated PKC isoforms even after short exposure times^13^. It is therefore extremely challenging to develop pharmacological agents capable of enhancing PKC signaling in a sustained way, while avoiding down-regulation or degradation^11,12^.

Tigilanol tiglate (TT, also known as EBC-46), is an epoxytigliane isolated from the seed of the native Australian rainforest plant *Fontainea picrosperma* (Euphorbiaceae)^14^. The presence of an α-oriented ring B epoxide, common in daphnane diterpenoids, is extremely rare in phorbol esters and complex to install by semi-synthesis from tiglianes^15^, explaining the paucity of data on the bioactivity of these natural products. TT was previously found to be an activator of Protein Kinase C (PKC) isoforms, and a single intratumoral dose of TT caused hemorrhagic necrosis and tumor ablation in melanoma, HNSCC and other mouse models of cancer^16,17^. TT was also found to induce a respiratory burst from human polymorphonuclear cells, and cause increased permeability of human umbilical vein endothelial cell monolayers. Furthermore, the anti-cancer mechanism of action of TT was found to be at least in part PKC-dependent, as the pan-PKC inhibitor bisindolylmaleimide-1 (BIS-1) partially inhibited efficacy^16^.

TT has recently been approved by the European Medicines Agency as a veterinary pharmaceutical for intratumoral treatment of non-metastatic, non-resectable mastocytomas in dogs. The drug is also under clinical evaluation for treatment of a range of other cutaneous and subcutaneous cancers in humans^18,19^, and companion animals^20^, and is showing a promising safety and efficacy profile. For example, in a Phase III veterinary trial for treatment of canine mastocytomas, complete and enduring response was recorded in 60 out of 80 patients^21^, while in a Phase I human safety/dose-escalation study, maximum tolerated dose was not reached and signs of efficacy were observed in 9 tumor types, including complete response in 4 patients^22^.

The clinical efficacy of a PKC-activating drug in treating a range of cancers provided a unique opportunity to gain further insights both into the complex role of PKC in cancer and into fundamental structure–activity relationships of epoxytiglianes relevant to their future development as new drug candidates. To address this potential, a detailed phytochemical investigation to obtain a library of epoxytiglianes and closely-related derivatives from *F. picrosperma* was undertaken. We present evidence of a link between activation of PKC isoforms and tumor regression after intratumoral administration.

## Results

### TT (EBC-46) and family member structure elucidation

**Figure 1:**
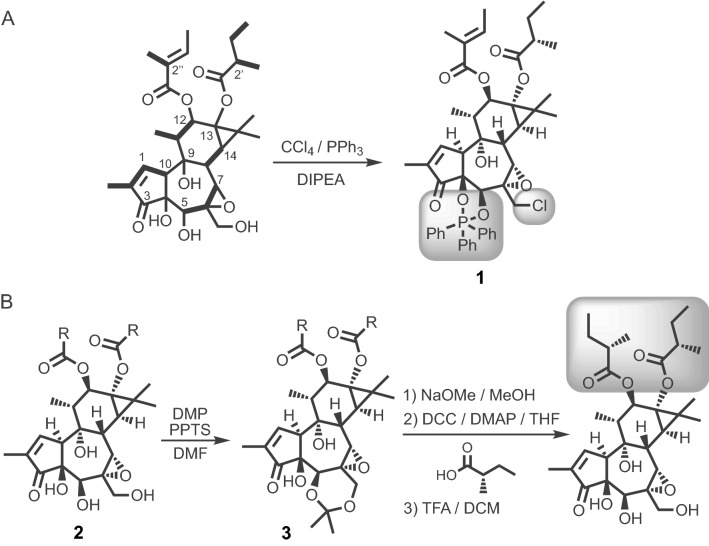
Chemical derivatization and semi-synthesis of TT. (**A**) Flat structure of TT with key COSY correlations in bold (left), and the dioxaphospholane derivative (**1**, right) revealing the absolute stereochemistry via x-ray crystallographic analysis. (**B**) Semi-synthesis of EBC-47 to deduce the absolute stereochemistry of the C12 ester at C2′’ [R = naturally occurring mixed esters; *DIPEA* *N*,*N*-diisopropylethylamine; *DMP* 2,2-dimethoxypropane; *PPTS* pyridinium *p*-toluensulfonate; *DMF* *N*,*N*-dimethylformamide; *DCC* *N*,*N*-dicyclohexylcarbodiimide; *DMAP* 4-*N*,*N*-dimethylaminopyridine; *THF* tetrahydrofuran; *TFA* trifluoroacetic acid; *DCM* dichloromethane].

Prior to this study only the flat structure of TT had been reported previously^14,16^, hence it was critical to determine the full stereochemical structure for stringent biological interpretation. In brief, a suitable crystalline derivative (**1**) (Fig. 1A) of TT was obtained using Appel conditions^23^, which confirmed all unassigned asymmetric centers and in turn the reported tigliane biosynthetic pathway^24^. Interestingly, x-ray crystallographic analysis of the tigliane system has only been sparsely reported^25–27^ reinforcing precise elucidation challenges for this class of diterpene.

**Figure 2:**
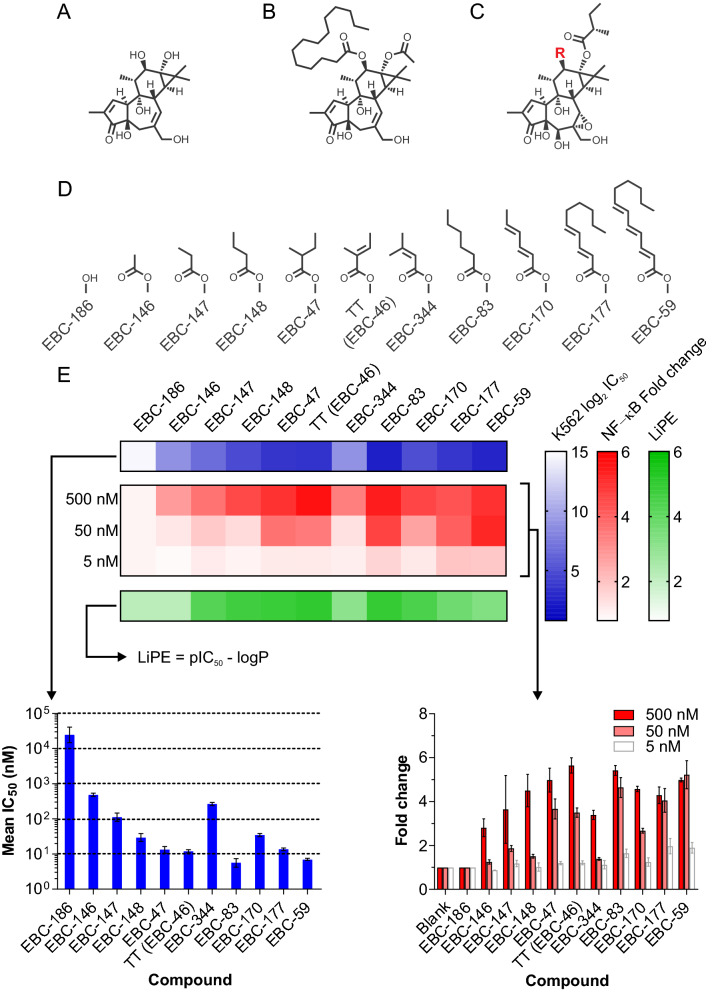
Different C12 ester length in epoxytiglianes purified from *Fontainea picrosperma* show varying potency. (**A**) Phorbol, and (**B**) PMA. (**C**) The epoxytigliane backbone (C12, R). (**D**) EcoBiotics Compounds (EBC, R =) isolated from *Fontainea picrosperma*. (**E**) Bioactivity of EBC analogues. K562 leukemia cell growth/survival assay (a surrogate for PKC activation) and NF-κB reporter system reveal differences in potency between EBC analogues. % Growth/Survival vs. Log_10_ [compound] curves were generated using K562 cells for the indicated compounds. These were subsequently used to calculate mean IC_50_ values (± 95% CI) (lower left panel), which was subsequently arranged into heatmap format (upper panel) for visualization. The fold change in luminescent signal from an NF-κB reporter assay was also determined for each epoxytigliane at three concentrations (500, 50 and 5 nM; lower right panel; mean ± SD). These data were converted into log_2_ values and displayed in heatmap format (upper panel). The lipophilic efficiency (LiPE) of each epoxytigliane was calculated using pIC_50_ values [-log_10_(IC_50_)] and experimentally determined logP values (see Supplementary data file 1). n = 2 or 3 for all EBC analogues.

**Figure 3:**
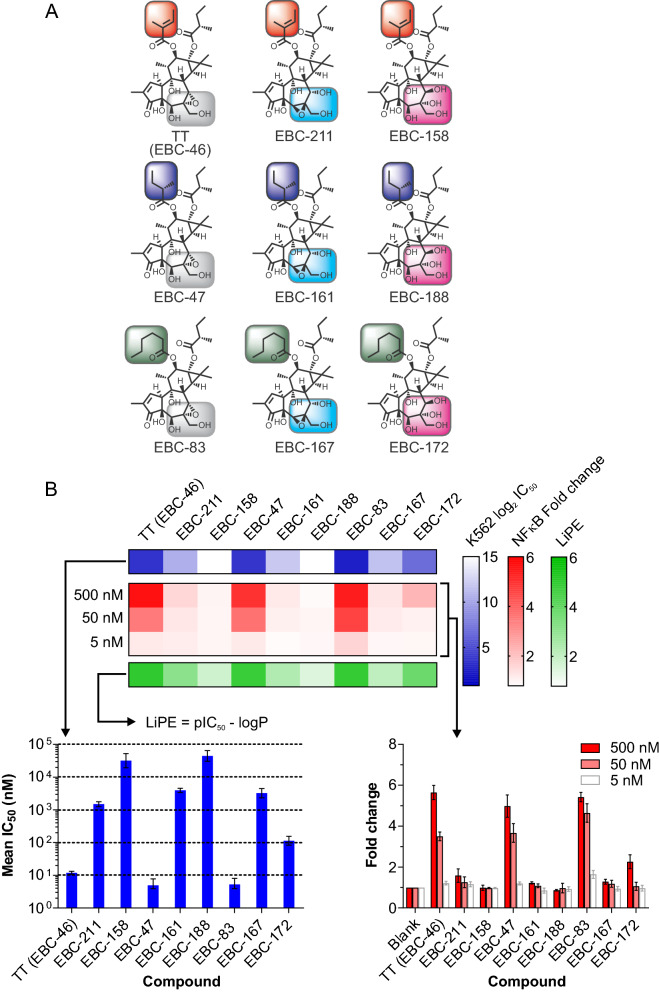
Changes in B-ring hydroxylation profile and C20 modification of epoxytiglianes purified from *Fontainea picrosperma* show varying potency. (**A**) EcoBiotics Compounds (EBC) isolated from *Fontainea picrosperma*; color highlights indicate structural similarities. (**B**) Bioactivity of EBC analogues. K562 leukemia cell growth/survival assay (a surrogate for PKC activation) and NF-κB reporter system reveal differences in potency between EBC analogues. % Growth/Survival vs. Log_10_ [compound] curves were generated using K562 cells for select tiglianes. These were subsequently used to calculate mean IC_50_ values (± 95% CI) (lower left panel), which was subsequently arranged into heatmap format (upper panel) for visualization. The fold change in luminescent signal from an NF-κB reporter assay was also determined for each tigliane at three concentrations (500, 50 and 5 nM; lower right panel; mean ± SD). These data were converted into log_2_ values and displayed in heatmap format (upper panel). The lipophilic efficiency (LiPE) of each tigliane was calculated using pIC_50_ values [− log_10_(IC_50_)] and experimentally determined logP values (see Supplementary data file 1). n = 2 or 3 for all EBC analogues.

Having fully elucidated **1** (Fig. 2A; Supplementary Figs. S1 and S2) it was a straightforward process to fully characterize TT and the additional 16 novel family members isolated through further investigation of *F. picrosperma*. Ten members were subsequently characterized that showed ordered side-chain variation, but only at C12 (i.e. EBC-47, -59, -83, 146–148, -170, -177, -186 and -344) (Fig. 2C, Supplementary Information data file S1, S2). Whereas three isolates were observed to have undergone a Payne rearrangement^28,29^, presumably induced by nucleophilic attack (S_N_2) by the adjacent hydroxyl group at C5, which resulted in epoxide formation at C5 and C6 (i.e. EBC-161, -167 and -211) (Fig. 3A, Supplementary Information Data File S1, S2). Furthermore, polyols EBC-158, -188 and -172 (Fig. 3A) were obtained as a likely result of epoxide (C6-C7) hydration, whether this conversion (or the Payne rearrangement) was a result of the isolation process (i.e., artefacts of isolation) is unknown at this stage. Lastly, in the case of EBC-47 the configuration of the C12 ester at C2″ was confirmed as *S* via a 4 step semi-synthesis (see **2** and **3**; Fig. 1B).

### Structure–activity analysis and assessment of cell growth inhibition of epoxytiglianes

The sixteen highly novel tiglianes, and TT (EBC-46) as the benchmark, were broken into two groups for structure–activity analysis and assessment of inhibition of cancer cell growth.

***Group 1*** contained an (*S*)-2-methylbutyrate at C13, allowing comparisons to focus on the length, branching and unsaturation of the C12 esters (Fig. 2C). The known growth inhibition of the K562 human myelogenous leukemia cell line at nM levels by the prototypic PKC activator PMA (Fig. 2B)^30^ was used to compare antineoplastic potential, integrating possible differences in drug uptake and PKC activation in vitro. The latter property was confirmed with the pan-PKC inhibitor bisindolylmaleimide I (BIS-1; Fig. S3), in line with our previous findings^16^.

As previously reported by others addressing straight-chain saturated C12 phorbol esters (e.g., PMA)^31–33^, it was found that in vitro bioactivity of the epoxytigliane esters increased with length (Fig. 2D) and hydrophobicity (LiPE^34,35^; Fig. 2E), with respect to potency of K562 growth inhibition (i.e., EBC-83 > EBC-148 > EBC-147 > EBC-146). The unsaturated esters, however, did not follow such a relationship, with EBC-177 (decan-2(*E*),4(*E*)-dienoyl) and EBC-59 (dodecan-2(*E*),4(*E*),6(*E*)-trienoyl) having similar or slightly less potency than EBC-83 (hexanoyl); while the bioactivity of EBC-170, containing a sorbate ester, had half the potency of EBC-83. NF-κB induction, another consequence of PKC activation^36^, was assessed using a reporter construct. Bioactivity of the epoxytiglianes in this assay followed a similar order as with K562 growth inhibition (Fig. 2E, Supplementary Table S1). PMA was more potent than the epoxytiglianes (Supplementary Fig. S3A). Branching of the C12 esters revealed substantial differences in activity; introduction of a 2-methyl in the case of EBC-47 increased potency compared with EBC-148 (butanoyl), whereas 3-methyl branching resulted in a tenfold loss of activity (i.e., EBC-344 vs TT). Loss of the C12 ester abolished the above bioactivities (i.e., EBC-186). Five of the above examples had similar potencies for growth inhibition compared with K562 when tested in a human breast cancer cell line (MCF7) of epithelial origin (Supplementary Fig. S3B).

***Group 2*** consisted of two B-ring modification types of TT, EBC-47 and -83 (Fig. 3A). Type 1 constituted the 5,6-epoxy-7-hydroxy isomer (e.g., EBC-211, -161 and -167 respectively), recognized as having undergone an epoxide Payne rearrangement^28^. Conveniently, additional amounts of these isolates were generated by semi-synthesis, which simply involved heating the parent compound at neutral pH. The 5,6-epoxides showed a striking, 2-log loss of bioactivity, as judged by inhibition of K562, induction of NF-κB (Fig. 3B) and inhibition of the growth of MCF7 cells (Supplementary Fig. S3B).

Type 2 was defined by epoxide hydration leading to ring opened 6,7-dihydroxy derivatives, which were also semi-synthesized via heating the natural 6,7-epoxides in mild acid. EBC-158 and EBC-188 were more than 3 logs less potent than the corresponding epoxides, whereas the hexanoate (i.e., EBC-172), was only 1 log less potent than its parent epoxide. These differences in activities were reflected in the lipophilic efficiencies (LiPE), which were high for the 6,7-epoxides, but much lower for the 5,6-epoxides and epoxide ring opened derivatives (Fig. 3B, Supplementary Table S1).

K562 growth inhibition was at least in part due to the activation of PKC, as pre-treatment with BIS-1 resulted in IC_50_ values approximately 4 orders of magnitude higher (Supplementary Fig. S3A). However, a direct correlation of growth inhibition with any specific PKC isoform expression was not possible (Supplementary Fig. S3B,C).

### PKC isoform translocation and signaling induced by epoxytiglianes

**Figure 4:**
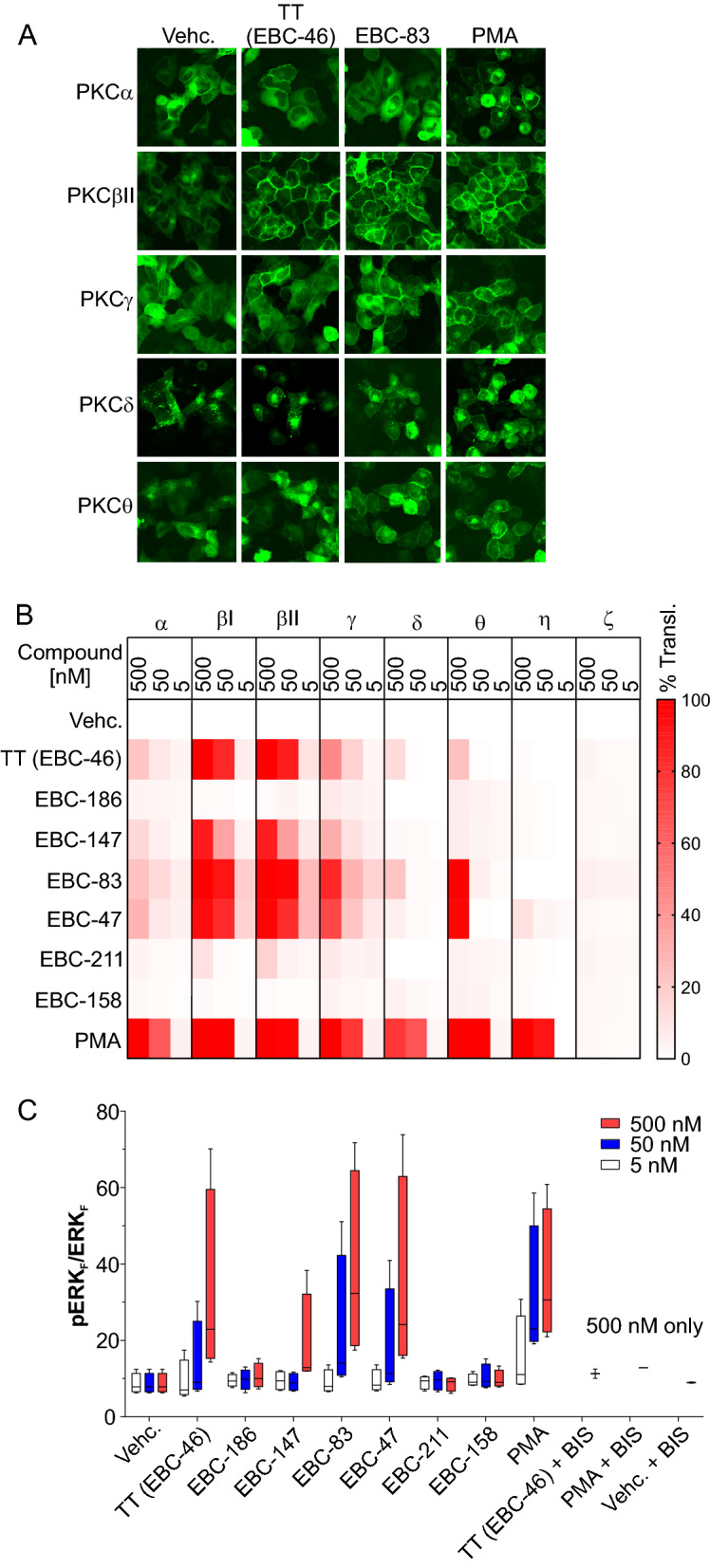
PKC isoform activation profile and ERK phosphorylation in response to epoxytigliane analogues. (**A**) Examples of PKCα, − βII, − γ, − δ and − θ translocation after treatment with vehicle (Vehc.) or 500 nM TT (EBC-46), EBC-83 or PMA. HeLa cells transfected with PKC-EGFP isoforms were incubated with the indicated concentrations of epoxytigliane for 1 h. Images were acquired using a GE InCell Analyzer 2000. These images were also used for the quantitation shown in (**B**). (**B**) Heatmap depicting PKC isoform translocation profile (− α, − βI, − βII, − γ, − δ, − θ, − η and − ζ—mean % of cells showing EGFP translocation to the plasma membrane) in response to 500, 50 and 5 nM TT (EBC-46) and the indicated analogues. > 150 cells counted per biological replicate. n = 3. (**C**) Analysis of ERK phosphorylation in response to administration of TT (EBC-46) and the indicated analogues. Cell extracts were analyzed using single plex phospho-ERK and total ERK alphaLISA kits from MM649 cells. Data expressed in box plot format (min to max values) using mean fluorescence intensity values (MFI) from phospho-ERK experiments normalized to MFI values from total ERK (p-ERK_F_/ERK_F_). n = 4.

Transient transfection of HeLa cells with PKC isoforms tagged to EGFP and subsequent treatment with either 500, 50 or 5 nM PMA, or the epoxytiglianes and analogues, for 1 h was used to assess the PKC isoform translocation profile of the selected derivatives (Fig. 4A). As expected, efficient translocation to the plasma membrane of the classical isoforms − α, − βI, − βII and − γ, as well as the novel − δ, − θ, and − η subtypes was observed with treatment with PMA (Fig. 4B). Interestingly, differing patterns of translocation were observed between high and moderate concentrations of PMA for PKC isoforms − α and − γ, where a mixed perinuclear/plasma membrane pattern was seen at 500 nM (Supplementary Fig. S4). No translocation was seen for the atypical PKC-ζ isoform lacking a C1 DAG-binding domain after treatment with PMA. Treatment with TT was consistent with previous studies^16^, with a high percentage of cells showing translocation of PKCβI and − βII isoforms, and less translocation seen for the − α, − γ, − δ, and − θ isoforms at both 500 and 50 nM (Fig. 4B). No translocation of the atypical PKC-ζ isoform was observed following TT treatment. In general, the 6,7-epoxytiglianes showed preference for translocation, and therefore activation, of PKC-βI and -βII isoforms. Additionally, the longer the carbon chain ester at C12, the more translocation was observed for PKCθ, with the exception of the branched chain case EBC-47. B-ring modifications, either type 1 (e.g., EBC-211) or type 2 (e.g., EBC-158) showed minimal translocation of any PKC isoforms in this assay (Fig. 4B; full data shown in Supplementary Fig. S4), supporting the above data. Assessing translocation of PKC-ε was not possible due to the toxicity of high levels of expression of this protein to cells following transient transfection, as previously reported^16^. Importantly, the observed translocation data likely corresponded with PKC activation, as treatment with the active analogues at the same time points showed increased phosphorylation of ERK1/2 in both MM649 (Fig. 4C) and HeLa cells (Supplementary Fig. S5), as well as increased cytokine release from peripheral blood mononuclear cells (Supplementary Fig. S6).

### Selected epoxytigliane analogues show anticancer efficacy in a xenograft melanoma model

**Figure 5:**
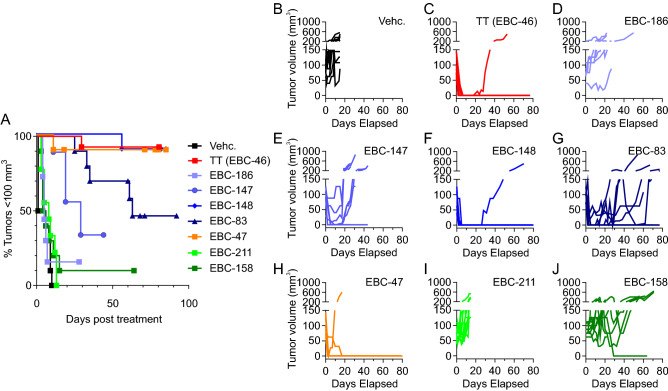
Varying efficacy of epoxytigliane analogues in an in vivo model of melanoma. (**A**) Kaplan Meier analysis of MM649 melanoma tumors reaching > 100 mm^3^ in BALB/c *Foxn1*^*nu*^ mice following single treatment with vehicle alone (40% PG) or 27 nmole of the indicated epoxytigliane. (**B**–**J**) Tumor volumes of MM649 tumors in BALB/c *Foxn1*^nu^ mice treated with 27 nmole of epoxytigliane analogues. (**B**) Vehicle only (Vehc.—40% PG), n = 10 tumors; (**C**) TT (EBC-46), n = 14 tumors; (**D**) EBC-186, n = 7 tumors; (**E**) EBC-147, n = 8 tumors; (**F**) EBC-148, n = 10 tumors; (**G**) EBC-83, n = 10 tumors; (**H**) EBC-47, n = 10 tumors; (**I**) EBC-211, n = 10 tumors; (**J**) EBC-158, n = 10 tumors.

Tumors established in BALB/c *Foxn1*^*nu*^ mice (“nude” mice, lacking T-cells) from intradermal injection of the human melanoma cell line MM649 were employed as a model for comparing the efficacies of TT and selected analogues administered by intratumoral injection. A dose response with TT showed that 27 nmol/tumor site, lower than that used previously^16^, was a suitable suboptimal level to allow comparison with analogues of higher or lower potency in formed tumors (average 100 mm^3^). Treatment with vehicle alone (40% propylene glycol in water) led to no decrease in tumor volume (Fig. 5B), with all tumors remaining over 100 mm^3^ (Fig. 5A). Treatment with TT (27 nmol) led to rapid reduction in tumor volume (Fig. 5C), with a single tumor regrowing 20 days after initial treatment (n = 14 tumors). Loss of the C12 ester (i.e., EBC-186) led to total loss of antitumor potency (Fig. 5D). For straight chain C12 esters, the butanoyl (EBC-148; Fig. 5F) was equivalent in potency to TT, whereas the acetyl (EBC-147) and hexanoyl (EBC-83) moieties (Fig. 5E,G, respectively) were less potent than TT with relapse in all but one tumor in each case. The result for the hexanoyl was somewhat surprising compared to its significant potency in vitro (Fig. 2E). EBC-47 (Fig. 5H) had similar potency to TT, with only a single tumor recurrence (n = 10 tumors). As found for their bioactivities in vitro, both of the B-ring modified derivatives EBC-211 (Fig. 5I) and EBC-158 (Fig. 5J) lacked efficacy against MM649 melanoma lesions, with no reduction of tumor volume observed apart from a single case with EBC-158.

**Figure 6:**
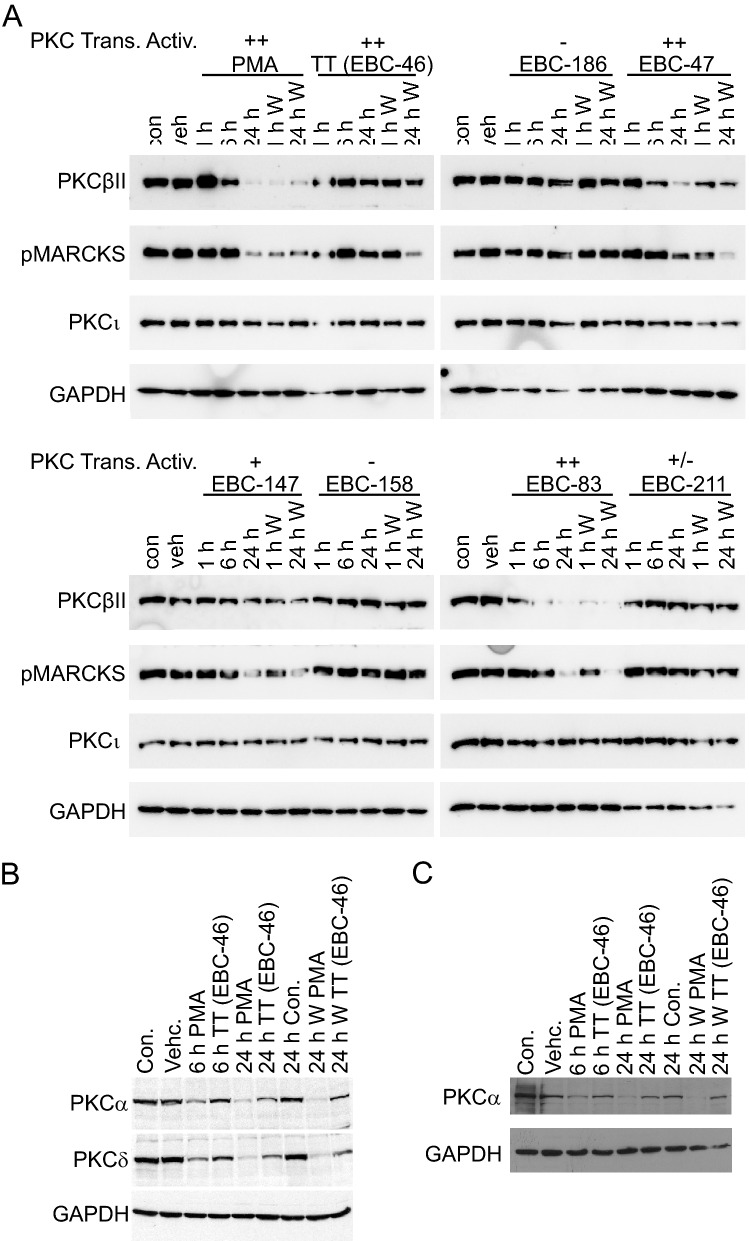
Limited PKC isoform degradation in cells following selected epoxytigliane treatment. (**A**) Western blot analysis of MM649 melanoma cells treated with 500 nM epoxytigliane analogues for the indicated times before harvesting. W—cells exposed to the epoxytiglianes for the indicated times before removal, washing and normal media for an additional 24 h prior to harvesting. (**B**,**C**) Western blot analysis of (**B**) SK-MEL-28 human melanoma or (**C**) B16-F0 murine melanoma cells treated with 500 nM TT (EBC-46) or PMA for the indicated times prior to harvesting. W—cells exposed to the epoxytiglianes for the indicated times before removal, washing and normal media for an additional 24 h prior to harvesting.

The effect on PKC isoform level and downstream signaling pathways following in vitro treatment of MM649 melanoma cells with the TT analogues on PKC isoform level and downstream signaling pathways were assessed to correlate with in vivo tumor ablation efficacy. PMA was again used as a positive control, and showed reduction of protein levels of PKCβ following 24 h treatment of MM649 melanoma cells, likely from proteolytic degradation as previously reported^37^. In addition, levels of phosphorylated MARCKS, a direct PKC substrate, were also significantly reduced following 24 h of treatment with PMA (Fig. 6A). As per previous studies, 1 or 24 h exposure, and subsequent washing out of PMA, along with further incubation for 24 h clearly demonstrated extended decreased levels of PKCβ and phosphorylated MARCKS. Notably, this pattern was replicated by EBC-83, an epoxytigliane demonstrating modest efficacy in vivo. However, exposure of other 6,7-epoxytiglianes to MM649 melanoma cells (Fig. 6A) did not lead to short term loss of PKC isoforms. While some reduction in phosphorylated protein level of direct downstream targets was observed with extended treatment of the most potent epoxytiglianes (i.e., TT or EBC-47 at 24 h treatment and 24 h recovery), detectable levels of PKC isoforms were maintained for those exhibiting the highest efficacy against MM649 tumors in vivo. No reduction in protein level of the atypical isoform PKCι was observed following short or prolonged treatment with any epoxytigliane or analogue, suggesting the effect is mediated by the PKC C1 domain. The ongoing presence of PKC isoforms following treatment with active epoxytiglianes was not unique to MM649 cells; treatment of SK-MEL-28 melanoma (Fig. 6B) or B16-F0 murine melanoma cells (Fig. 6C) with TT showed maintenance of PKC isoform protein level compared to PMA even with 24 h exposure and 24 h recovery from treatment.

## Discussion

The hallmark of most tigliane diterpenoids from *F. picrosperma* is the presence of multiple oxidation on the B-ring and a tigliate group on the ring C13-hydroxyl, with differences limited to the acyl decoration of the 12-hydroxyl and the glicidyl/triol nature of the trioxygenated ring B fragment. The configuration of the trioxygenated fragment on the B-ring was critical for bioactivity, since the 5-hydroxy-6,7-epoxy motif outperformed its regioisomeric (5,6-epoxy-7-hydroxy)- and the ring opened (triol) examples, the latter being almost inactive. Within the linear C12 alkyl esters, growth inhibition increased with chain length, as found in the phorbol esters^31–33^, but this relationship did not hold for alkenyl esters, where restricted conformational flexibility might interfere with membrane insertion/transport. The (*S*)-2-methylbutanoyl is a notable motif for both C12 and C13 esters. This residue had a significant influence on bioactivity, presumably related to an increasing hydrophobicity and more limited conformational freedom close to the polycyclic skeleton, an increased stability to esterase attack in vivo^38^, or a combination of both effects.

The test cell line K562 expresses both classical and novel PKC isoforms, similar in expression pattern to MM649 (Supplementary Fig. S3C). However, these cell lines vary greatly in their sensitivities to growth inhibition by epoxytiglianes, implicating potential differences in downstream signaling or activation of alternative C1 domain proteins in addition to PKC activation. Nevertheless, a dissection between growth inhibition in vitro and antitumor potency was observed, the latter peaking with efficacy of TT. In vitro activity data on a variety of cell types could be rationalized in terms of ester hydrophobicity and 6,7-epoxide-dependent PKC activation, but antitumor action selectively emphasized the 6,7-epoxide structural element, being optimal for TT and EBC-148, but declining with higher or lower levels of ester hydrophobicity, the latter attributed to less PKC activation. However, the effect on tumor growth and cell viability in vivo may not be a direct effect of the compounds on the tumor cells, given the apparent insensitivity to the compounds in vitro. These results likely indicate that in vivo efficacy may act through other mechanisms than those seen in vitro. There are many instances where sensitivity to anti-cancer drugs differ between in vitro and in vivo or 3D situations^39–41^. This apparent contradiction between sensitivities to this class of natural products in vitro versus in vivo has been previously observed for TT^16^. We previously identified MM649 cells to be relatively insensitive to TT in vitro*.* In contrast, treatment in vivo with 50 nmol TT lead to 90% ablation of MM649 tumors. Subsequent experiments showed a rapid loss of cell viability when tumors were treated in vivo, potentially due to the effect of TT on intratumoral vasculature^16^. Moreover, the in vivo efficacy of TT was shown to be, at least in part, attributable to activation of PKC. Treatment with BIS-1 prior to TT significantly reduced the efficacy of treatment, demonstrating the role of PKC^16^.

Previous studies have outlined the role of neutrophils in antitumor efficacy of PKC agonists^42,43^. In keeping, intralesional injection of active epoxytiglianes into tumors in BALB/c *Foxn1*^*nu*^ mice induces an initial swelling that recedes after days, potentially suggesting influx of components of the innate immune system. Our previous work with TT showed a minor role for neutrophils in antitumor efficacy with the use of blocking antibodies^16^. However, recent results have potentially highlighted the recruitment of neutrophils following treatment as an important factor in TT efficacy^17^. Therefore, the efficacy of the active epoxytiglianes may be due to the effects of the class on the innate immune system, in particular recruitment and activation of neutrophils, and warrants further investigation.

The TT analogues with modest activity in K562 growth inhibition, translocation of PKC and subsequent downstream phosphorylation of signaling pathways, resulted only in an initial tumor volume reduction (i.e., EBC-147, Fig. 5E). Whereas family members with low bioactivity against PKC and downstream signaling, showed no reduction of tumor volume following intralesional treatment (i.e. EBC-211, Fig. 5I and EBC-158, Fig. 5J). The 6,7-epoxytiglianes showing strong K562 growth inhibition, translocation of PKC and phosphorylation of signaling pathways could ablate established tumors upon intralesional injection (i.e., TT, Fig. 5C; EBC-47, Fig. 5H). Epoxytiglianes with acetyl (EBC-147; Fig. 5E) and hexanoyl (EBC-83; Fig. 5G) moieties showed reduced in vivo efficacy although they exhibited PKC activation. The loss of antitumor potency by EBC-83, the *n*-hexanoate analogue of TT, was surprising in the light of its higher hydrophobicity and PKC activation capacity compared to TT. While the reason for these discrepancies are not immediately clear, it appears that EBC-147 does not elicit as strong PKC activation or downstream signaling as EBC-148 or TT (Fig. 1, Figs. S5 and S6). It is also possible that tumor ablation in the melanoma xenograft model may be less dependent on PKC activation and more reliant on alternate targets, potentially including but not limited to those proteins containing a C1 domain for this group of natural epoxytiglianes and analogues. Further investigation of novel targets of the epoxytiglianes, as well as activation of alternate downstream signaling pathways is therefore needed.

The role of PKC activation in the antitumor activity of other phorboids is unclear. The 12-deoxyphorbol ester prostratin has been reported to reduce tumor volume in mouse models of pancreatic cancer^44^, but is only a weak modulator of PKC, while the activity of ingenol-3-angelate (marketed as ingenol mebutate or Picato used for treatment of actinic keratosis^45,46^ and basal cell carcinoma^47^) may also involve additional targets^48^.

In vivo antitumor efficacy of epoxytiglianes is consistent with a role for PKC activation, as evidenced by isoform translocation, activation of downstream signaling and increased cytokine production. Remarkably, and in sharp contrast with the archetypal phorbol ester PMA, treatment with the most efficacious epoxytiglianes was associated with retention of detectable intracellular levels of the PKCβ isoforms. The molecular details of this biased activation are unknown, but tumor ablation in the melanoma xenograft model appears to be dependent on PKC activation in addition to longevity of isoform stability and downstream signaling for this group of natural epoxytiglianes and analogues. In addition, activation of PKCθ mostly corresponded with in vivo efficacy. Interestingly, both PKCθ and PKCβ are known to activate NF-κB signalling^49,50^. Importantly, the epoxytiglianes without evidence of PKC activation showed limited antitumor efficacy. These combined factors are suggestive of a proposition that the potent antitumor activity observed with several of the epoxytiglianes arises from stimulation of PKC signaling. This hypothesis is supported by recent findings suggesting particular PKC isoforms may act as tumor suppressors, and not as oncoproteins as long thought^5,11,12^ and that in certain contexts, PKC may be considered a tumor suppressor^5,51^. The role of PKC isoforms in cancer is extremely complex and often contradictory, likely due to the effects being cell lineage specific. For example, PKCα inhibits cell motility in breast cancer^52^, but promotes migration of colon cancer^53^. Further, over-expression of PKCβ promotes breast cancer cell growth^54^, while other studies suggest PKCβ expression reduces breast cancer tumor formation^55^. Complete understanding of the specific roles of different PKC isoforms in cancer formation and progression remains an outstanding goal. In any case, our results suggest a reassessment of the role of PKC in cancer may be necessary, refocusing drug discovery efforts on activation rather than inhibition of a specific set of isoforms of this family of enzymes.

## Methods

### 1D and 2D NMR Spectra

1D and 2D NMR spectra were recorded as previously described^56^. Additional information is provided in the Supplementary Information document.

### X-ray crystallography

X-ray crystallographic data for compound **1** were collected on an Oxford Diffraction Gemini CCD diffractometer with Cu-Kα radiation (1.54184 Ǻ) operating within the range 2 < 2θ < 125°. Additional methods are included in the Supplementary Information document. The thermal ellipsoid plot (Supplementary Fig. S1) was produced with ORTEP (v1.0.3; https://www.chem.gla.ac.uk/~louis/software/ortep/index.html) and the simplified structure diagram, without H-atoms, was drawn with PLATON (https://www.platonsoft.nl/platon/). Crystallographic data including structure factors in CIF format have been deposited with the Cambridge Crystallographic Data Centre (CCDC 1889589). The disorder in the isobutyl group was resolved with the alkyl group occupying alternate positions related by a 180° rotation of the C–CO bond. The chloromethyl group was also disordered about by a slight twist of the C-CH2Cl bond. The contributors were modelled with complementary occupancies. This disorder is illustrated in Supplementary Fig. S2.

### Reagents

All natural and semi-synthetic epoxytiglianes were solubilised in 100% ethanol at 20 mg/ml (approx. 34–36 μM, dependent on compound) prior to dilution. Phorbol myristate acetate (PMA) was also solubilized in 100% ethanol at 20 mg/ml. Bisindoylmaleimide-I (BIS) was solubilized in 100% ethanol at 2 mM.

### Cell lines

Neonatal foreskin fibroblasts (NFF), K562 (leukemia), MCF7 (breast carcinoma), HeLa (cervical carcinoma), MM649 (melanoma), SK-MEL-28 (melanoma), FaDu (head and neck squamous cell carcinoma of the pharynx) and B16-F0 (mouse melanoma) were cultured in RPMI-1640 media supplemented with 10% fetal calf serum (FCS). Cells were maintained in a humidified incubator at 37 °C with 5% CO_2_. The identity of all human cell lines was confirmed via STR profiling and mycoplasma status was routinely assessed using MycoAlert (Lonza).

### Cell growth/survival assays

Cell growth/ survival of non-adherent K562 cells (3 × 10^3^ cells per well) was determined using the CellTiter 96 AQueous One Solution cell proliferation assay kit as previously described^57^. Cell growth/survival of adherent cell lines (3 × 10^3^ cells per well) was determined using a sulforhodamine B (SRB) assay as previously detailed^16^. Additional information can be found in Supplementary Information.

### NF-κB reporter assays

HeLa cells stably transfected with a NF-κB-luciferase reporter construct or vector lacking the promoter sequence as a negative control, were plated into black 96-well plates (Corning #3606) at a density of 2 × 10^4^ cells per well in 100 μl of media. The following day, media containing compound at a final concentration of 500 nM, 50 nM or 5 nM was subsequently administered to wells in duplicate. Vehicle (ethanol) only controls were also compiled and administered. 24 h after compound addition, 100 μl of luciferase substrate from a Steady-Glo Luciferase Assay System kit (Promega) was added to each well. The luminescence signal from each well was then measured using a H4 Hybrid Synergy plate reader (Biotek) (Gain: 135, time 5 s). Corrected luminescence values from treated wells were compared to untreated wells and results plotted as fold change in luminescence signal using Prism (v8.2.1, GraphPad; https://www.graphpad.com/scientific-software/prism/).

### Immunoblotting

Whole cell extracts (WCE) were prepared from cell pellets using sodium dodecyl sulfate (SDS) lysis buffer (10 mM Tris–HCl, pH 7.4, 100 mM NaCl, 1% SDS, 20% glycerol), supplemented with cOmplete protease and PhosSTOP phosphatase inhibitor cocktails (Roche). Pellets were solubilized using a 3:1 ratio of lysis buffer to pellet, after which samples were sonicated using a Branson Sonifier 250: Power Output 3, 30% Duty Cycle, 15 pulses). Insoluble material was removed via centrifugation (16,000×*g*) and protein concentration determined using a BCA assay kit (Pierce). Samples were separated via SDS-PAGE and transferred to nitrocellulose for immunoblotting as previously described^16^. The following antibodies were used in this study: anti-PKCα (#610107, 1:1000), anti-PKCγ (#611158, 1:1000), anti-PKCδ (#610397, 1:1000), anti-PKCε (#610085, 1:1000), anti-PKCη (#610814, 1:1000), anti-PKCθ (#610089, 1:1000) and anti-PKCι (#610175, 1:1000) were purchased from BD Transduction Laboratories. Anti-PKCζ (#9372, 1:1000) was acquired from Cell Signaling Technology, anti-PKCβ (ab38279) was acquired from AbCam and anti-GAPDH (#2275-PC-7, 1:5000) from R&D Systems.

### PKC translocation assays

HeLa were plated at 2 × 10^4^ cells per well into clear bottom, black 96-well plates (Corning #3606) in preparation for transient transfection with plasmids encoding EGFP-tagged PKC isoforms: PKCα, PKCβI, PKCβII, PKCγ, PKCδ, PKCθ, PKCη and PKCζ^16^. Plasmid/lipofectamine complexes were prepared as follows. Plasmid (0.16 µg) and Lipofectamine 2000 (0.48 µl; Life Technologies) were individually mixed with 25 µl Opti-MEM (Life Technologies) and incubated for 5 min at room temperature (RT). These solutions were combined and incubated for another 20 min at RT. The resultant complexes (50 µl) were then added to their respective wells. After 3 h incubation at 37 °C in 5% CO_2_, another 50 µl of media was added and the plate incubated overnight under the same conditions. After 24 h, cells were washed 2 × with PBS and treated with 500, 50 and 5 nM of compound, prediluted in media (100 µl). After 1 h treatment, the cells were washed × 2 with PBS and fixed with 50 µl of 2% formaldehyde/0.2% glutaraldehyde in PBS for 10 min at RT. Cells were then washed × 2 with PBS and nuclei stained using Hoechst 33342 (1:10,000 dilution in PBS for 7 min). Dye was removed, cells washed 3 × with PBS, 100 μl of PBS added to each well and the plate stored at 4 °C in the dark until imaging. Imaging was performed using a GE InCell Analyzer 2000 (GE). Several images were acquired per well and PKC isoform translocation to subcellular structures was assessed manually using Adobe Photoshop (vCS6; www.adobe.com). More than 150 cells were counted from each well, at least 50 cells per image.

### Mitogen activated protein kinase (MAPK) pathway activation

Quantitative detection of mitogen activated protein kinase (MAPK) pathway activation in response to compound addition was performed using AlphaLISA SureFire Ultra p-ERK1/2 (T202/Y204) and total ERK1/2 kits as per the manufacturer’s instructions (Perkin-Elmer). Here, 2.5 × 10^4^ HeLa or MM649 were seeded into a 96-well plate (Corning #3595) in media (100 μl) and incubated overnight at 37 °C in 5% CO_2_. After 24 h, cells were washed × 1 with PBS and treated with 500, 50 and 5 nM of compound, prediluted in media (100 µl). Cells were incubated for 1 h with compound as above, after which media was aspirated, cells washed once with PBS and lysed using 50 μl of kit derived lysis buffer. 10 μl of sample was incubated with 5 μl of donor and acceptor mix for 2 h prior to measurement using an Enspire alphascreen reader (Perkin-Elmer). Mean fluorescence intensity (MFI) values from phospho-ERK experiments were normalized to MFI values from total ERK (p-ERK_F_/ERK_F_) for each compound.

### Analysis of cytokine release from PBMCs

Human peripheral blood mononuclear cells (PBMCs) were isolated from heparinised blood (acquired from 3 different donors) by Ficol-Paque sedimentation. Briefly, whole blood was diluted 3:1 in prewarmed RPMI (no FCS) and layered on top of Ficol-Paque. Samples were centrifuged at 400×*g*, giving a red blood cell pellet and PBMC layer. The PBMC layer was harvested, washed × 3 with RPMI-1640 (no FCS) and finally resuspended in complete media. PBMCs were seeded at a density of 1.5 × 10^5^ cells per well in media and stimulated with compound at three concentrations (500, 50, 5 nM) in duplicate for 24 h. Media samples were taken from each of the required wells and frozen at − 80 °C until use. Each media sample was assayed for the presence of soluble IL-1β, IL-6, IL-8, IL-10, IL-12p70 and TNF using a CBA Human Inflammatory Cytokine Detection Kit according to the manufacturers’ instructions (Becton Dickinson). Mean fluorescence intensity values from each sample were compared against a standard curve to determine cytokine concentrations in cell culture supernatants (pg/ml ± S.D.) using FCAP Array analysis software (v3.0; http://www.bdbiosciences.com/cba).

### Xenograft tumor studies

Xenograft tumor studies were performed as previously described. Briefly, MM649 tumors (2 × 10^6^ cells in initial injection, 2 sites per BALB/c *Foxn1*^*nu*^ mouse) were grown to approximately 75–100 mm^3^, when mice were treated with either vehicle (40% propylene glycol in water, 50 µl), or 25 nmol of compound in vehicle (50 µl), via a single intra-tumoral injection. Tumor volume was measured twice weekly, recorded using digital calipers and expressed as mm^3^ according to the formula A × b × b × 0.5 (A = length, b = breadth).

### Human ethics statement

This study was performed in strict accordance with the recommendations in the Australian National Statement on Ethical Conduct in Human Research (2007—Updated December 2013), of the National Health and Medical Research Council of Australia. All protocols were reviewed and approved by the QIMR Berghofer Medical Research Institute Human Research Ethics Committee (QIMR-HREC, NHMRC HREC #EC00278), approval numbers P726 and P764. All participants provided written informed consent to donate their samples for research.

### Animal ethics statement

All experimental procedures were approved by the QIMR Berghofer Medical Research Institute Animal Ethics Committee (QIMRB-AEC): A0106-042 M and A0404-606 M. This study was performed in accordance with the recommendations in the Australian Code for the Care and Use of Animals for Scientific Purposes 8th Edition (2013; National Health and Medical Research Council of Australia).

### Statistical analysis

All values reported in this study represent the mean with either SD or SEM as indicated in each of the Fig. legends. The number of biological replicate experiments is also indicated in each of the Fig. legends. Statistical analysis was performed using Prism v8.2.1 (GraphPad; https://www.graphpad.com/scientific-software/prism/).

## Supplementary Information

Supplementary Information.

## Data Availability

Crystallographic data including structure factors in CIF format have been deposited with the Cambridge Crystallographic Data Centre (CCDC 1889589).

## References

[1] Castagna M (1982). Direct activation of calcium-activated, phospholipid-dependent protein kinase by tumor-promoting phorbol esters. J. Biol. Chem..

[2] Steinberg SF (2008). Structural basis of protein kinase C isoform function. Physiol. Rev..

[3] Hirai T, Chida K (2003). Protein kinase Czeta (PKCzeta): activation mechanisms and cellular functions. J. Biochem..

[4] Mochly-Rosen D, Das K, Grimes KV (2012). Protein kinase C, an elusive therapeutic target?. Nat. Rev. Drug Discov..

[5] Antal CE (2015). Cancer-associated protein kinase C mutations reveal kinase's role as tumor suppressor. Cell.

[6] Pongracz J, Clark P, Neoptolemos JP, Lord JM (1995). Expression of protein kinase C isoenzymes in colorectal cancer tissue and their differential activation by different bile acids. Int. J. Cancer.

[7] Suga K, Sugimoto I, Ito H, Hashimoto E (1998). Down-regulation of protein kinase C-alpha detected in human colorectal cancer. Biochem. Mol. Biol. Int..

[8] Dowling CM (2016). Protein kinase C beta II suppresses colorectal cancer by regulating IGF-1 mediated cell survival. Oncotarget.

[9] Mandil R (2001). Protein kinase Calpha and protein kinase Cdelta play opposite roles in the proliferation and apoptosis of glioma cells. Cancer Res..

[10] Lu HC (2009). Analysing the expression of protein kinase C eta in human hepatocellular carcinoma. Pathology.

[11] Newton AC, Brognard J (2017). Reversing the paradigm: Protein kinase C as a tumor suppressor. Trends Pharmacol. Sci..

[12] Newton AC (2018). Protein kinase C as a tumor suppressor. Semin. Cancer Biol..

[13] Lu Z (1998). Activation of protein kinase C triggers its ubiquitination and degradation. Mol. Cell Biol..

[14] Reddell, P. W. & Gordon, V. A. TIGLIEN-3-ONE DERIVATIVES. International Patent Number WO 2007/070985 A1. WO 2007/070985 A1 (2007).

[15] Boudreault PL, Mattler JK, Wender PA (2015). Studies on the regio- and diastereo-selective epoxidation of daphnanes and tiglianes. Tetrahedron Lett..

[16] Boyle GM (2014). Intra-lesional injection of the novel PKC activator EBC-46 rapidly ablates tumors in mouse models. PLoS ONE.

[17] Barnett CME (2019). Optimising intratumoral treatment of head and neck squamous cell carcinoma models with the diterpene ester Tigilanol tiglate. Investig. New Drugs.

[18] ACTRN:12614000685617. *Australian and New Zealand Clinical Trials Registry [Internet]: Identifier ACTRN12614000685617. Phase I Dose-Escalation Study to Determine the Safety and Tolerability of an Intratumoural Injection of EBC-46. *www.anzctr.org.au/ACTRN12614000685617.aspx, 2017).

[19] ACTRN12619001407189. *Australian and New Zealand Clinical Trials Registry [Internet]: Identifier ACTRN12619001407189. Exploratory Phase Ib/IIa Study of Intratumourally Administered Tigilanol Tiglate to Assess Safety, Tolerability and Tumour Response in Patients with Head and Neck Squamous Cell Carcinoma*. www.anzctr.org.au/ACTRN12619001407189.aspx (2019).

[20] Miller J (2019). Dose characterization of the investigational anticancer drug tigilanol tiglate (EBC-46) in the local treatment of canine mast cell tumors. Front. Vet. Sci..

[21] De Ridder TR (2020). Randomized controlled clinical study evaluating the efficacy and safety of intratumoral treatment of canine mast cell tumors with tigilanol tiglate (EBC-46). J. Vet. Intern. Med..

[22] Panizza BJ (2019). Phase I dose-escalation study to determine the safety, tolerability, preliminary efficacy and pharmacokinetics of an intratumoral injection of tigilanol tiglate (EBC-46). EBioMedicine.

[23] Williams CM, Mander LN, Bernhardt PV, Willis AC (2005). Investigating direct routes to an advanced intermediate for the synthesis of C-20 diterpene alkaloids. Tetrahedron.

[24] Wang HB, Wang XY, Liu LP, Qin GW, Kang TG (2015). Tigliane diterpenoids from the Euphorbiaceae and Thymelaeaceae families. Chem. Rev..

[25] Brandl F, Rohrl M, Zechmeister K, Hoppe W (1971). Rontgenstrukturanalysen von Neophorbol C31H35O9Br und Phorbol C20H28O6. Acta Crystallogr. Sect. B.

[26] Nothias LF (2017). Environmentally friendly procedure based on supercritical fluid chromatography and tandem mass spectrometry molecular networking for the discovery of potent antiviral compounds from *Euphorbia semiperfoliata*. J. Nat. Prod..

[27] Pettersen RC, Birnbaum GI, Ferguson G, Islam KMS, Sime JG (1968). X-Ray investigation of several phorbol derivatives. The crystal and molecular structure of phorbol bromofuroate–chloroform solvate at –160°. J. Chem. Soc. B..

[28] Payne GB (1962). Epoxide migrations with α, β-epoxy alcohols. J. Org. Chem..

[29] Hanson, R. M. *Epoxide Migration (Payne Rearrangement) and Related Reactions*. (2004).

[30] Koeffler HP, Bar-Eli M, Territo MC (1981). Phorbol ester effect on differentiation of human myeloid leukemia cell lines blocked at different stages of maturation. Cancer Res..

[31] Fujiki H (1980). Relationship between ornithine decarboxylase-inducing activity and configuration at C-4 in phorbol ester derivatives. J. Cancer Res. Clin. Oncol..

[32] Wender PA (1988). Modeling of the bryostatins to the phorbol ester pharmacophore on protein kinase C. Proc. Natl. Acad. Sci. USA.

[33] Wender PA, Koehler KF, Sharkey NA, Dell'Aquila ML, Blumberg PM (1986). Analysis of the phorbol ester pharmacophore on protein kinase C as a guide to the rational design of new classes of analogs. Proc. Natl. Acad. Sci. USA.

[34] Leeson PD, Springthorpe B (2007). The influence of drug-like concepts on decision-making in medicinal chemistry. Nat. Rev. Drug Discov..

[35] Ryckmans T (2009). Rapid assessment of a novel series of selective CB(2) agonists using parallel synthesis protocols: A Lipophilic Efficiency (LipE) analysis. Bioorg. Med. Chem. Lett..

[36] Busuttil V (2002). Blocking NF-kappaB activation in Jurkat leukemic T cells converts the survival agent and tumor promoter PMA into an apoptotic effector. Oncogene.

[37] Cozzi SJ, Parsons PG, Ogbourne SM, Pedley J, Boyle GM (2006). Induction of senescence in diterpene ester-treated melanoma cells via protein kinase C-dependent hyperactivation of the mitogen-activated protein kinase pathway. Cancer Res..

[38] Carotti A (2006). Lipophilicity plays a major role in modulating the inhibition of monoamine oxidase B by 7-substituted coumarins. Chem. Biodivers..

[39] Hongisto V (2013). High-throughput 3D screening reveals differences in drug sensitivities between culture models of JIMT1 breast cancer cells. PLoS ONE.

[40] Shannan B (2016). Enhancing the evaluation of PI3K inhibitors through 3D melanoma models. Pigment Cell Melanoma Res..

[41] Han K (2020). CRISPR screens in cancer spheroids identify 3D growth-specific vulnerabilities. Nature.

[42] Challacombe JM (2006). Neutrophils are a key component of the antitumor efficacy of topical chemotherapy with ingenol-3-angelate. J. Immunol..

[43] Li L (2010). The skin cancer chemotherapeutic agent ingenol-3-angelate (PEP005) is a substrate for the epidermal multidrug transporter (ABCB1) and targets tumor vasculature. Cancer Res..

[44] Wang MT (2015). K-Ras promotes tumorigenicity through suppression of non-canonical Wnt signaling. Cell.

[45] Lebwohl M (2012). Ingenol mebutate gel for actinic keratosis. N. Engl. J. Med..

[46] Stockfleth E (2018). Phase IV head-to-head randomized controlled trial comparing ingenol mebutate 0.015% gel with diclofenac sodium 3% gel for the treatment of actinic keratosis on the face or scalp. Br. J. Dermatol..

[47] Izzi S, Sorgi P, Piemonte P, Carbone A, Frascione P (2016). Successfully treated superficial basal cell carcinomas with ingenol mebutate 005% gel: Report of twenty cases. Dermatol. Ther..

[48] Parker CG (2017). Chemical proteomics identifies SLC25A20 as a functional target of the ingenol class of actinic keratosis drugs. ACS Cent. Sci..

[49] Lin X, O'Mahony A, Mu Y, Geleziunas R, Greene WC (2000). Protein kinase C-θ participates in NF-κB activation induced by CD3-CD28 costimulation through selective activation of IκB kinase β. Mol. Cell. Biol..

[50] Moscat J, Diaz-Meco MT, Rennert P (2003). NF-kappaB activation by protein kinase C isoforms and B-cell function. EMBO Rep..

[51] Adams RA (2015). Ectopic expression of protein kinase C-beta sensitizes head and neck squamous cell carcinoma to diterpene esters. Anticancer Res..

[52] Gauthier ML, Torretto C, Ly J, Francescutti V, O'Day DH (2003). Protein kinase Calpha negatively regulates cell spreading and motility in MDA-MB-231 human breast cancer cells downstream of epidermal growth factor receptor. Biochem. Biophys. Res. Commun..

[53] Masur K, Niggemann B, Zanker KS, Entschladen F (2001). Norepinephrine-induced migration of SW 480 colon carcinoma cells is inhibited by beta-blockers. Cancer Res..

[54] Li H, Weinstein IB (2006). Protein kinase C beta enhances growth and expression of cyclin D1 in human breast cancer cells. Cancer Res..

[55] Manni A (1996). Induction of a less aggressive breast cancer phenotype by protein kinase C-alpha and -beta overexpression. Cell Growth Differ..

[56] Maslovskaya LA (2020). EBC-232 and 323: A structural conundrum necessitating unification of five in silico prediction and elucidation methods. Chemistry.

[57] Tran TD (2020). Potent antibacterial prenylated acetophenones from the australian endemic plant *Acronychia crassipetala*. Antibiotics (Basel)..

