# Supplementary material for: Activation of PKC supports the anticancer activity of tigilanol tiglate and related epoxytiglianes
Source: Sci Rep. 2021 Jan 8;11:207. doi: 10.1038/s41598-020-80397-9 (PMC7794351; doi:10.1038/s41598-020-80397-9)

Activation of PKC supports the anticancer activity of tigilanol tiglate and related epoxytiglianes

Jason K. Cullen[1], Glen M. Boyle[1,2,3,*], Pei-Yi Yap[1], Stefan Elmlinger[1], Jacinta L. Simmons[1], Natasa Broit[1], Jenny Johns[1], Blake Ferguson[1], Lidia Maslovskaya[1,4], Andrei I. Savchenko[4], Paul Malek Mirzayans[4], Achim Porzelle[4], Paul V. Bernhardt[4], Victoria A. Gordon[5], Paul W. Reddell[5], Alberto Pagani[6], Giovanni Appendino[6], Peter G. Parsons[1,3] and Craig M. Williams[4,*].

[1]Drug Discovery Group, QIMR Berghofer Medical Research Institute, Brisbane, Queensland, Australia

[2]School of Biomedical Sciences, Faculty of Health, Queensland University of Technology, Brisbane, Queensland, Australia

[3]School of Biomedical Sciences, Faculty of Medicine, University of Queensland, Brisbane, Queensland, Australia

[4]School of Chemistry and Molecular Biosciences, University of Queensland, Brisbane, Queensland, Australia

[5]QBiotics Group, Yungaburra, Queensland, Australia; [6]Dipartimento di Scienze del Farmaco, Università degli Studi del Piemonte Orientale, Largo Donegani 2, 28100 Novara, Italy.

[*]To whom correspondence may be addressed. E-mail: Glen.Boyle@qimrberghofer.edu.au or c.williams3@uq.edu.au

*Correspondence: Glen M. Boyle, QIMR Berghofer Medical Research Institute, Locked Bag 2000, Royal Brisbane Hospital, Brisbane, QLD 4029, Australia. Phone: +61 7 3362 0319. Email: Glen.Boyle@qimrberghofer.edu.au
Craig M. Williams, School of Chemistry and Molecular Biosciences, University of Queensland, Brisbane, QLD 4072, Australia. Phone: +61 7 3365 3530. Email: c.williams3@uq.edu.au

Running title: PKC activity and anticancer activity of epoxytiglianes

Key words: Protein Kinase C, epoxytigliane, activation, anticancer treatment, *in vivo* efficacy.

SUPPLEMENTARY FIGURES

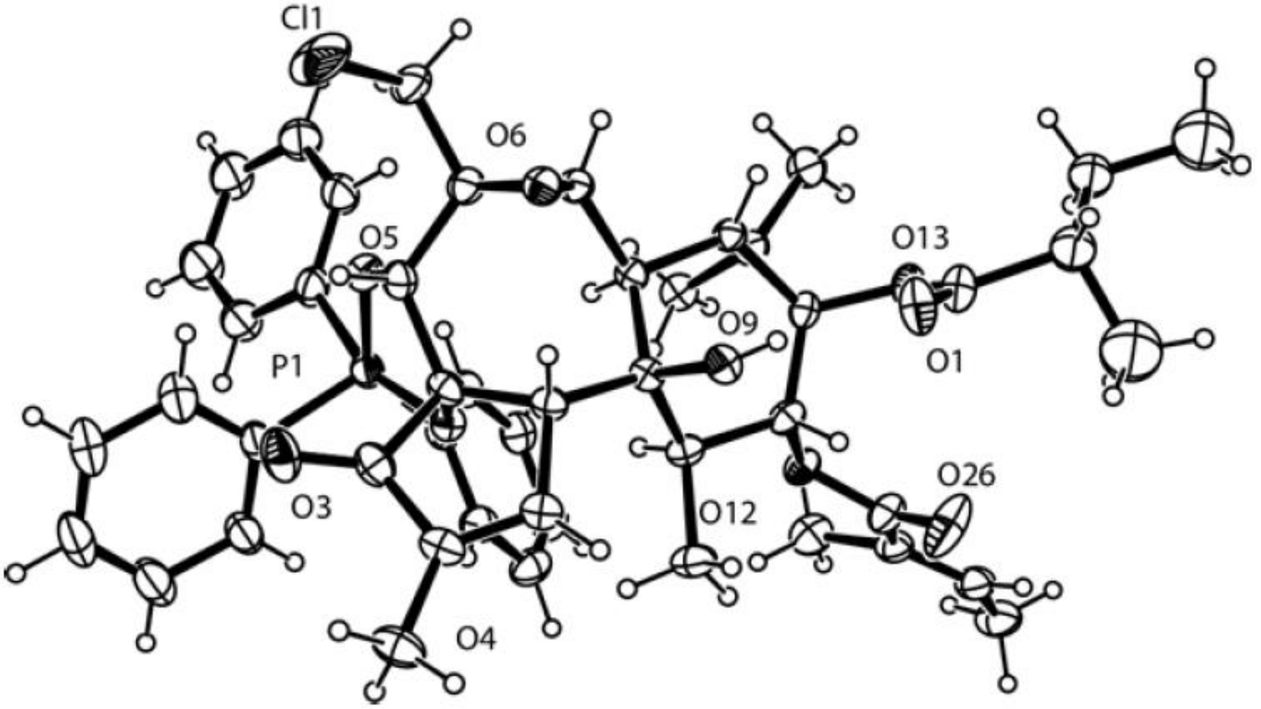

Figure S1. ORTEP drawing of compound 1
30% probability ellipsoids shown. Disorder not shown for clarity.

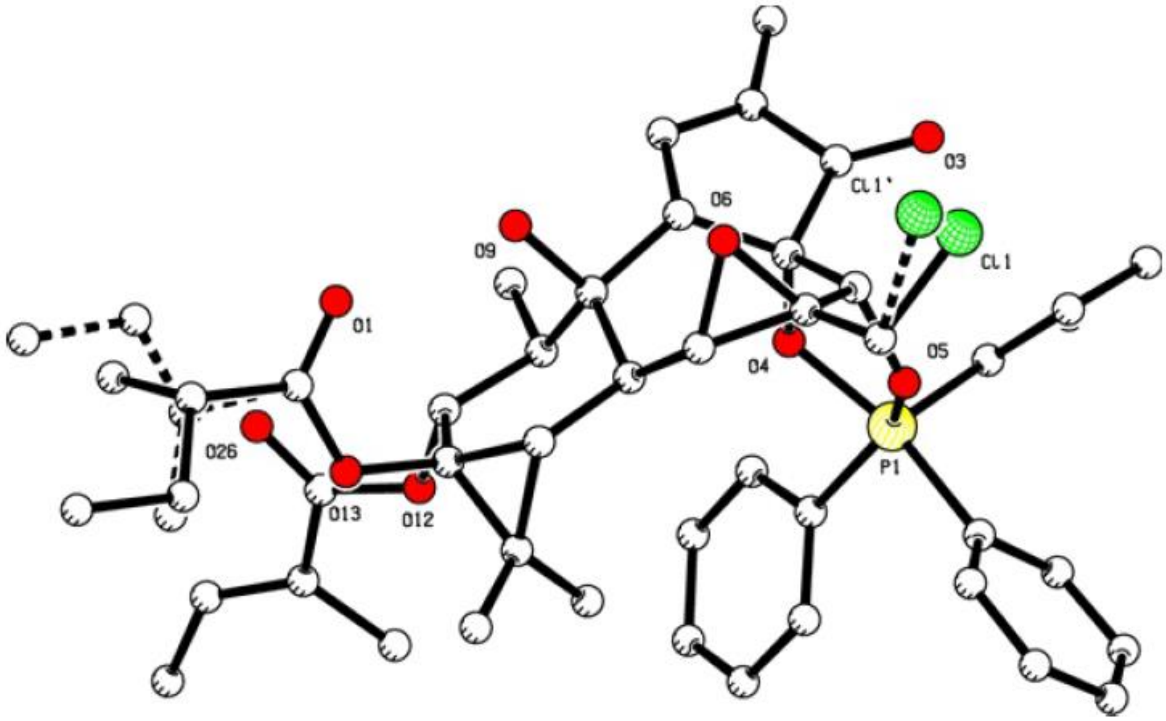

Figure S2. PLATON drawing of compound 1
Figure showing disorder; H-atoms omitted for clarity.

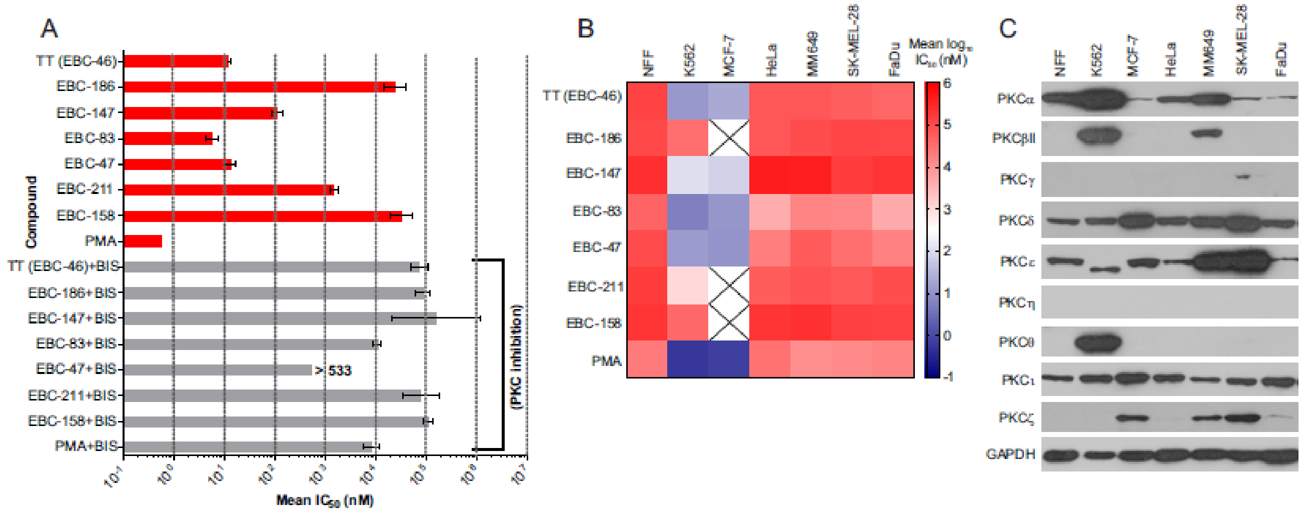

Figure S3. Differences in epoxytigliane potency are observed between various cancer cell lines
(A) K562 IC_{50} values of selected natural epoxytigliane analogues. Grey bars: PKC inhibition prior to epoxytigliane treatment with 4 µM BIS.
(B) Heatmap depicting Mean log_{10} IC_{50} values of TT (EBC-46) and the selected epoxytigliane analogues (determined from % Growth/Survival vs. [compound] plots - MTS or SRB assay) in the indicated cell lines. PMA is shown for comparison. n = 2 or 3 for all analogues.
(C) PKC isoform expression in primary (NFF) and a selection of cancer cell lines (K562 – leukemia; MCF-7 – breast; HeLa – cervical; MM649 and SK-MEL-28 – melanoma; FaDu – tongue squamous cell carcinoma). Whole cell extracts (WCEs) separated via SDS-PAGE and immunoblotting performed with antibodies to PKCα, -β, -γ, -δ, -ε, -η, -θ, -ι, -ζ and GAPDH. 20 µg protein per lane.

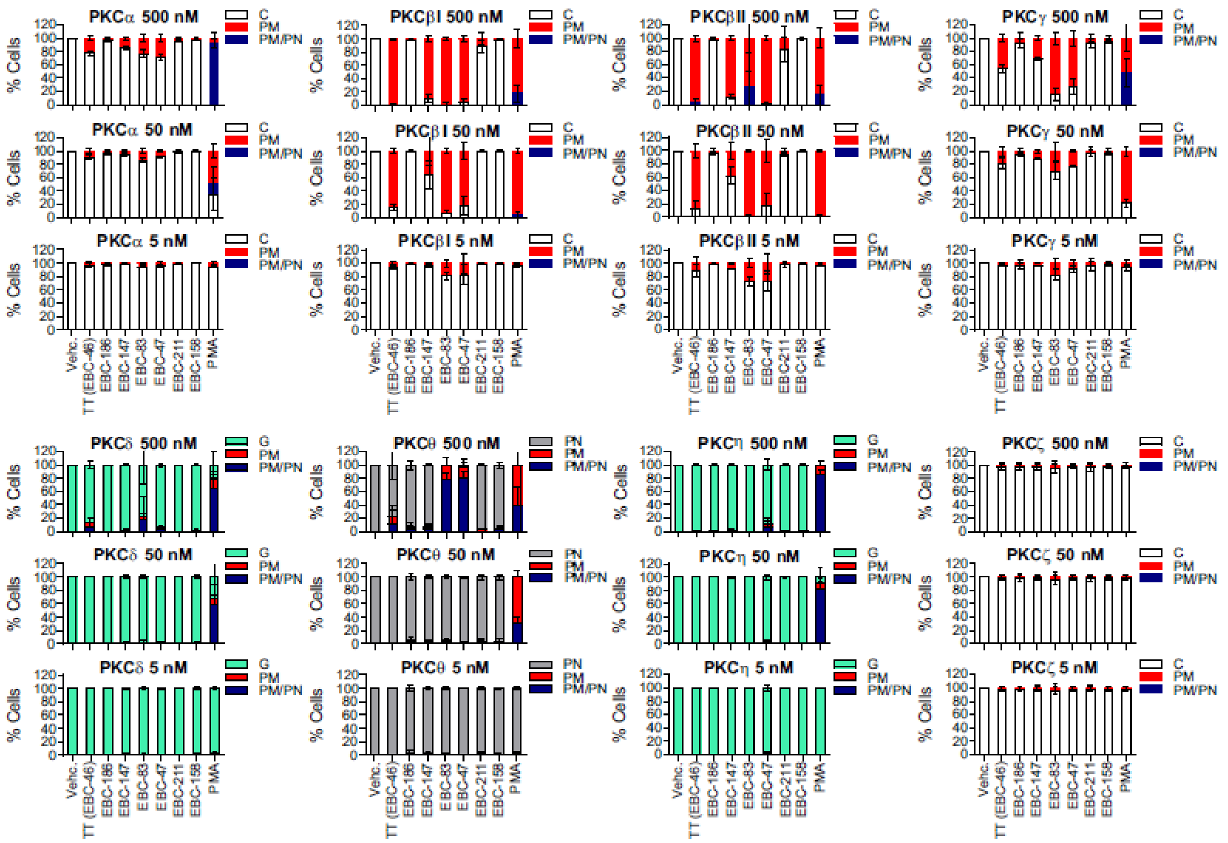

Figure S4. Complete PKC translocation profiles for the epoxytigliane analogues used in this study
The percentage of cells (± SD) showing cytoplasmic (C), granular (G), perinuclear (PN), plasma membrane (PM) or plasma membrane/perinuclear staining (PM/PN) are shown for each isoform and compound tested. Experiments performed as detailed in Figure 4.

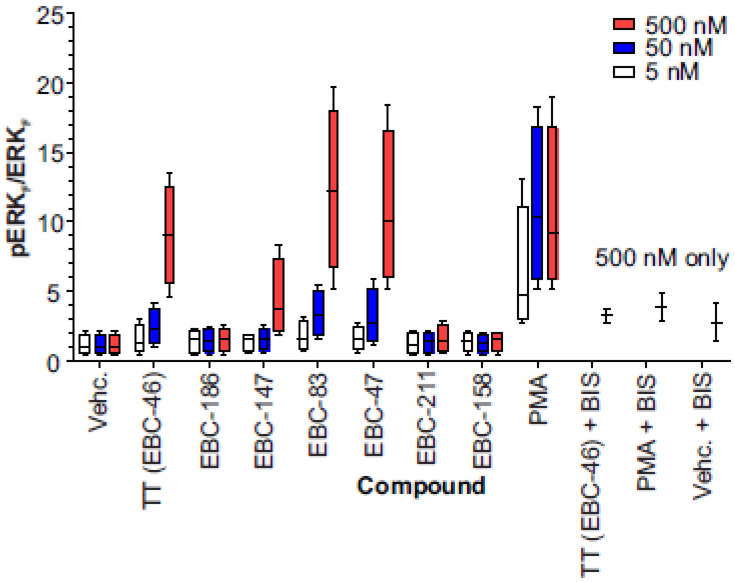

Figure S5. ERK phosphorylation in response to epoxytigliane analogues in HeLa cells
Analysis of ERK phosphorylation in response to administration of TT (EBC-46) and the indicated analogues. Extracts from HeLa cells were analyzed using single plex phospho-ERK and total ERK alphaLISA kits. Data expressed in box plot format using mean fluorescence intensity values (MFI) from phospho-ERK experiments normalized to MFI values from total ERK ($p\text{-}ERK_F/ERK_F$). n = 4.

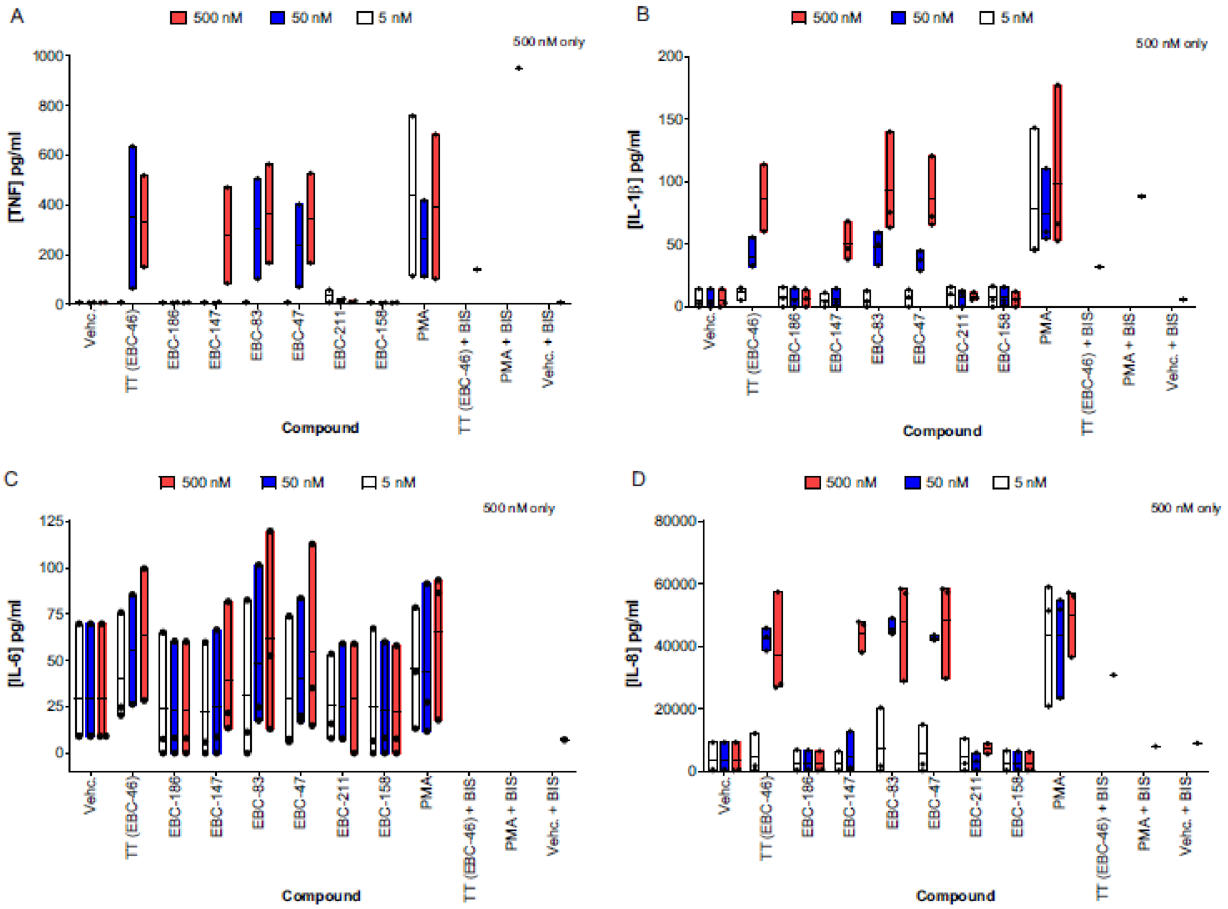

Figure S6. Epoxytiglianes promote proinflammatory cytokine release from perpheral blood mononuclear cells (PBMCs)

PBMCs isolated from 3 different individuals were treated with 500, 50 or 5 nM of compound for 24 h in RPMI-1640, 10% FCS media. Supernatants were removed and (A) TNF, (B) IL-1β, (C) IL-6 and (D) IL-8 levels determined in each sample using a CBA human inflammatory cytokine kit (BD Biosciences). All samples run in duplicate. n = 3 for all data except TNF where n = 2. Data expressed as pg/ml (range is shown with median value – bar).

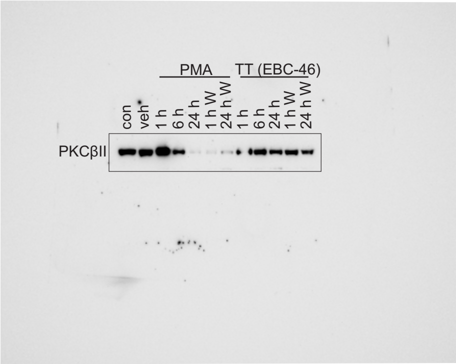

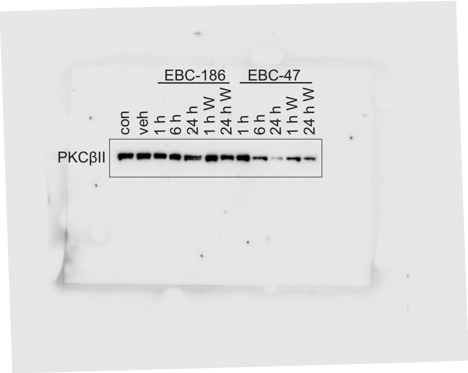

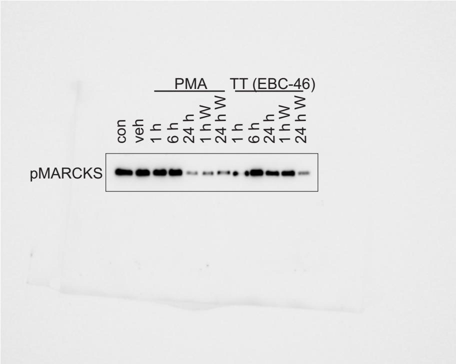

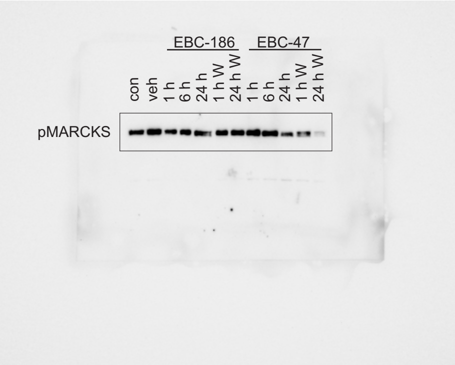

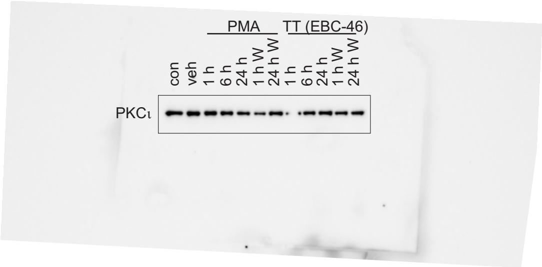

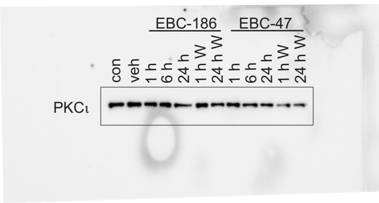

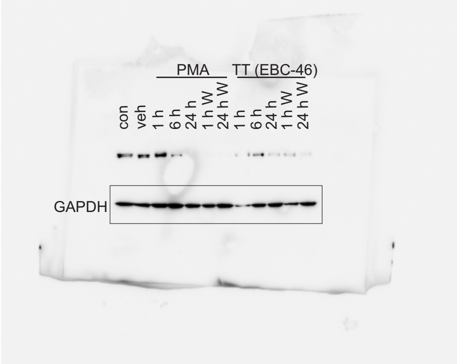

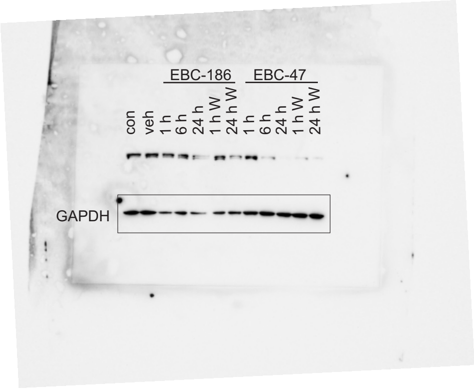

Supplementary Figure 7 - uncropped images relating to Figure 6

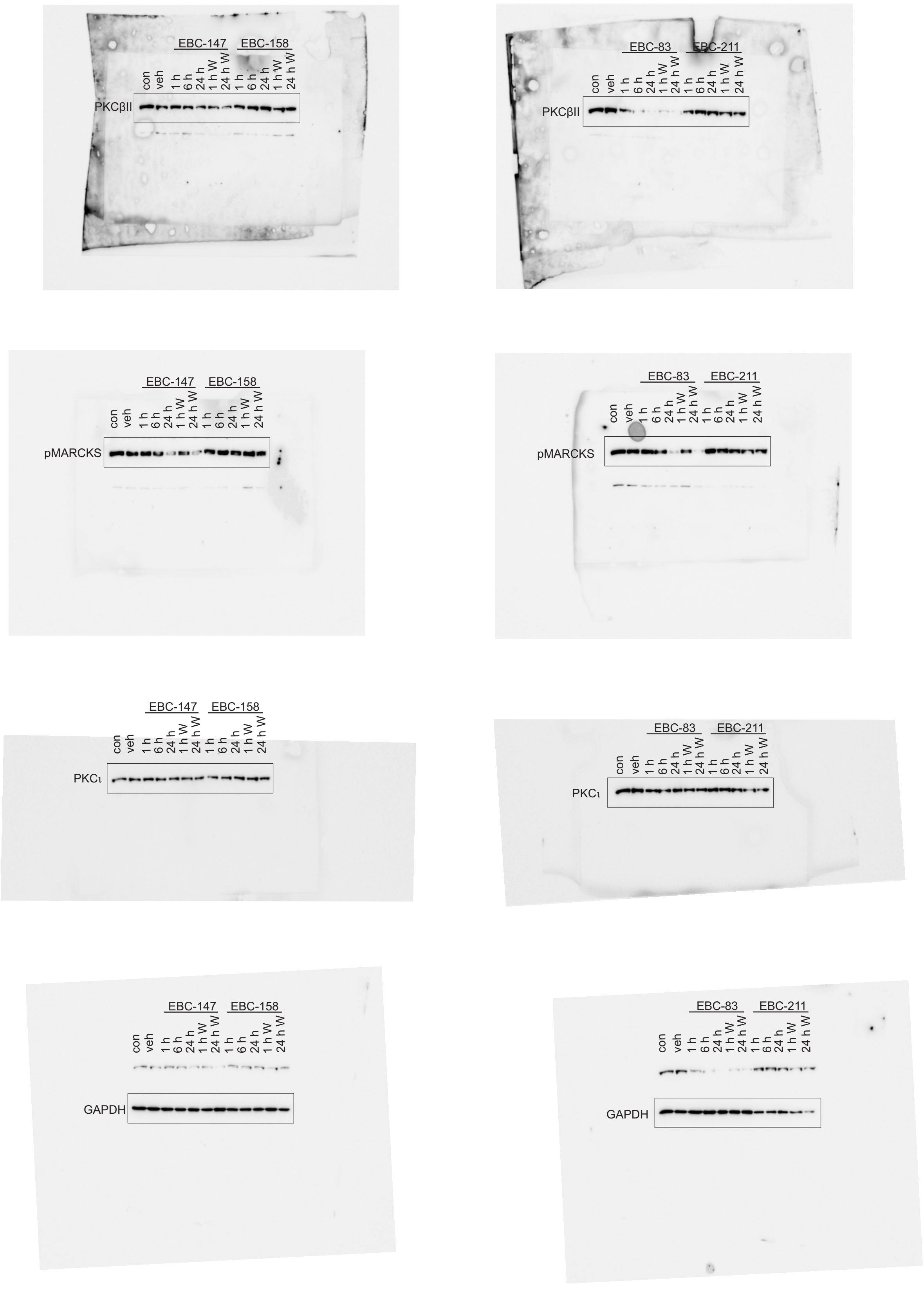

Supplementary Figure 7 - uncropped images relating to Figure 6

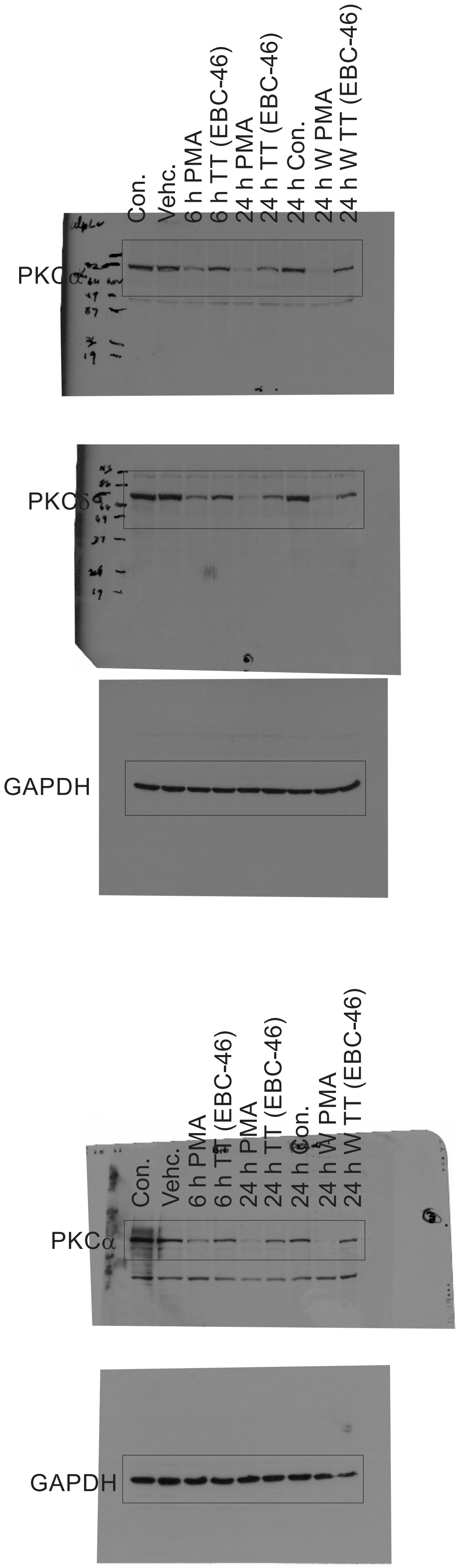

Supplementary Figure 7 - uncropped images relating to Figure 6

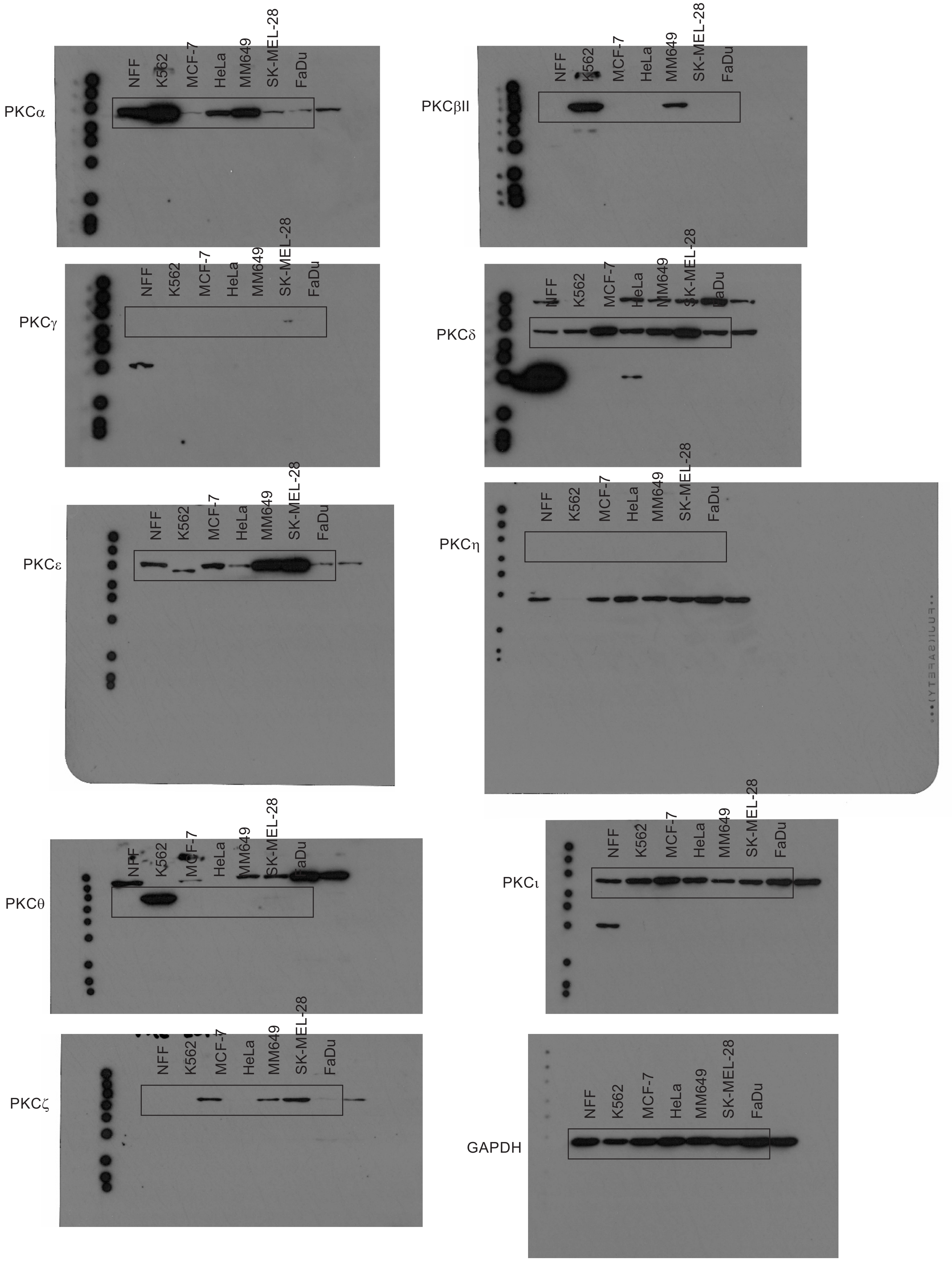

Supplementary Figure 8 - uncropped images relating to Figure S3C

Supplementary Table S1 - Characteristics and bioactivity of epoxytiglianes

Compound	Structural Features			M_r	M^{+23} assayed	Purity in tested sample (%UV 249nm)	K562 SURVIVAL: mean IC_{50} nM (95% CI).			
	C12 ester	C13 ester	Other				Exp1	Exp 2	Exp 3	with BIS-1
TT (EBC-46)	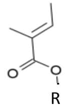	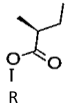		562.27	585.4	99.3	11.9 (10.8-13.3)			72276
EBC-47	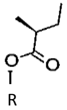	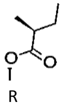		564.27	587.4	98.7	13.2 (10.5-16.4)			>533
EBC-59	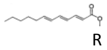	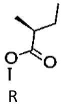		656.8	679.4	86.4	6.9 (6.6-7.5)			>457
EBC-83	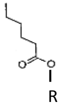	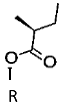		578.69	601.5	99.4	5.6 (4.2-7.4)			10764
EBC-146	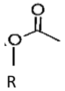	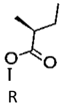		522.58	545	96.9	481.9 (433.5-535.8)		[redacted]	>19135
EBC-147	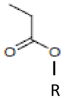	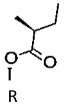		536.61	559	98.5	111.7 (85.3-146.6)		[redacted]	161808
EBC-148	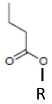	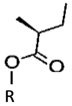		550.64	573.2	98.1	29.4 (22.7-38.1)			>544

EBC-158	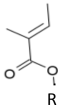	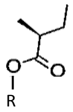	C6 and C7 OH. No C6C7 epoxy.	580.66	603.8	98.1	31915 (19319-52844)	109395
EBC-161	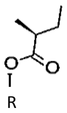	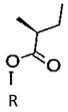	C7 OH. No C6C7 epoxy or C5 OH. C5C6 epoxy.	564.66	587.1	99.8	3935.5 (3372.9-4591.98)	N.D.
EBC-167	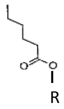	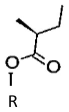	C7 OH. No C6C7 epoxy or C5 OH. C5C6 epoxy.	578.69	601.5	99.6	3228.5 (2317.4-4487.4)	N.D.
EBC-170	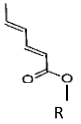	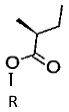		574.66		97.9	34.6 (31.1-38.5)	N.D.
EBC-171	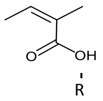	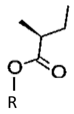	No C6C7 epoxy.	546.65	569.7	96.1	14.4 (11.2-18.4)	N.D.
EBC-172	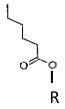	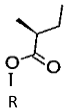	C6 and C7 OH. No C6C7 epoxy.	596.71	619.4	99.9	113.2 (82.8-155.2)	N.D.
EBC-177	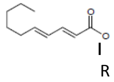	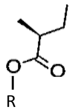		630.77	653.77	98.1	13.7 (12.5-14.9) [redacted]	>1585
EBC-186	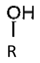	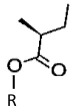		480.55	502.9	95.4	24490 (14689-40831)	86297
EBC-188	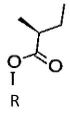	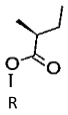	C6 and C7 OH. No C6C7 epoxy.	582.68	605.3	96.1	44463.1 (30549.2-64714.3)	N.D.

EBC-211	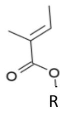	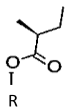	C7 OH. No C6C7 epoxy or C5 OH. C5C6 epoxy.	562.65	585.4	100
EBC-344	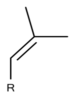	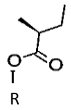		562.64	585.3	92.6
PMA	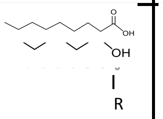	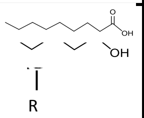	No C6C7 epoxy	616.83	639	91

1496.2 (1258.9-1774.2)		79615
264.9 (237.7-295.1)		N.D.
0.55 (0.51-0.58)		8511

Absolute IC50 Values calculated from Log plots in Prism using Log(inhibitor) vs. response - Variable slope algorithm. Mean IC50 values (nM) shown (95% CI in brackets)

BIS-1 : bisindolylmaleimide-I (cPKC inhibitor). Survival of K562 were determined via MTS based assay.

Compound	LogP	LiPE	Molar K562 IC_{50}
TT (EBC-46)	2.91	5.014453039	1.19E-08
EBC-47	3.06	4.819426069	1.32E-08
EBC-59	4.85	3.311150909	6.9E-09
EBC-83	3.32	4.931811973	5.6E-09
EBC-146	2.56	3.757043074	4.819E-07
EBC-147	2.68	4.271946827	1.117E-07
EBC-148	2.84	4.69165267	2.94E-08

EBC-158	2.73	1.766005151	0.000031915
EBC-161	3.05	2.355000083	3.9355E-06
EBC-167	3.41	2.080999209	3.2285E-06
EBC-170	3.01	4.450923901	3.46E-08
EBC-171	2.94	4.901637508	1.44E-08
EBC-172	3.15	3.796153573	1.132E-07
EBC-177	4.14	3.723279433	1.37E-08
EBC-186	2.43	2.181011215	0.00002449
EBC-188	2.87	1.482000261	4.44631E-05

EBC-211	2.87	2.95501035	1.4962E-06
EBC-344	3.22	3.356918042	2.649E-07
PMA	7.36 (cLogP)	0.516148359	1.33E-08
		Lipophillic efficiency (LiPE) calculated using following equation: LiPE = -log10(IC50)-LogP.	

SUPPLEMENTARY INFORMATION – METHODS

General Experimental Procedures

Optical rotations were measured using a Jasco P-2000 spectrophotometer. IR spectra were obtained using an ATR FT-IR spectrometer Perkin Elmer SPECTRUM 2000 and UV spectra were obtained with a Perkin Elmer Lambda-35 UV/vis spectrometer. 1D and 2D NMR spectra were recorded using the following spectrometers: Bruker DRX500 (^{1}H NMR 500.13 MHz; ^{13}C NMR 125.77 MHz), Bruker Avance AV-750 (^{1}H NMR - 750.13 MHz; ^{13}C NMR 187.53 MHz), Bruker 700 Avance III HD (^{1}H NMR 700.48 MHz; ^{13}C NMR 176.15 MHz) and Brucker Avance-900 (^{1}H NMR - 900.14 MHz; ^{13}C NMR 226.36 MHz) both equipped with cryoprobe in $CDCl_3$: δ in ppm, J in Hz. Low resolution electrospray ionisation mass spectrometry measurements (LRESIMS) were recorded in positive or negative ionization mode on a Bruker Esquire HCT (High Capacity 3D ion trap) instrument with a Bruker ESI source. High resolution electrospray ionisation (HRESIMS) accurate mass measurements were recorded in positive mode on a Bruker MicrOTOF-Q (quadrupole – Time of Flight) instrument with a Bruker ESI source. Accurate mass measurements were carried out with external calibration using sodium formate as the reference calibrant. LCMS was performed using a Shimadzu Prominence HPLC coupled to a SCIEX API 3200 mass spectrometer and run on a Halo RP-amide column (4.6 mm × 150 mm × 2.7 µm) in a 45-95% acetonitrile gradient containing 0.1% formic acid. HPLC instruments include HPLC Agilent 1190 and Gilson Fraction collector FC204. Column chromatography (CC): Silica gel 60 (200-300 mesch, 15-40 µ, Merck, Germany). Semi-preparative HPLC was carried out on a Phenomenex Luna 5 µm C18 (2), 250 × 15.00 mm, 5 µ column. HPLC separation using analytical column was made on Phenomenex Luna C18(2) 100A, 250 × 4.6 mm, 5 µ column. Additional methods are included in the Supplementary Information document.

X-ray Crystallography

X-ray crystallographic data for compound **1** were collected on an Oxford Diffraction Gemini CCD diffractometer with Cu-Kα radiation (1.54184 Å) operating within the range $2 < 2\theta < 125°$. The sample was cooled to 173 K with an Oxford Cryosystems Cryostream Cooler (600 series). Data reduction and empirical absorption corrections were performed using CrysAlisPro (Oxford Diffraction). The structure was solved by Direct Methods and refined with SHELX and all calculations were carried out within the WinGX package. All non-H atoms were refined aniostropically except the disordered isobutyl group and the minor contributor to the disordered chloromethyl group. The absolute structure was determined by anomalous dispersion. The thermal ellipsoid plot (Supplementary Fig. S1) was produced with ORTEP and the simplified structure diagram, without H-atoms, was drawn with PLATON. Crystallographic data

including structure factors in CIF format have been deposited with the Cambridge Crystallographic Data Centre (CCDC 1889589). The disorder in the isobutyl group was resolved with the alkyl group occupying alternate positions related by a 180° rotation of the C-CO bond. The chloromethyl group was also disordered about by a slight twist of the C-CH_2Cl bond. The contributors were modelled with complementary occupancies. This disorder is illustrated in Supplementary Fig. S2.

Isolation and purification

Fontainea picrosperma kernels (collected on a number of occasions at Upper Barron, Queensland; 17°27' S, 145°30' E; QBiotics herbarium voucher YA2040) were crushed and homogenized in ethanol (4 volumes). The oil obtained by evaporation of solvent was partitioned via column chromatography on silica gel (heptane: ethyl acetate) into pooled fractions. Fractions were then further purified by HPLC isocratically using methanol/water. Compounds were initially characterized by retention time (RRT) (A249 nm) relative to EBC-46 (TT) and by M+23 mass, determined by LCMS.

EBC-46: Retention time 8.5 min (RRT=1), M+23 585.4, prep 73% methanol/5 ml/min.

EBC-47: RRT 1.14, M+23 587.4, prep 73% methanol/5 ml/min.

EBC-59: RRT 2.20, M+23 679.4, prep 85% methanol/5 ml/min

EBC-83: RRT 1.47, M+23 601.6, prep 75% methanol 5 ml/min.

EBC-146: RRT 0.49, M+23 545.0, prep 70% methanol/8 ml/min.

EBC-147: RRT 0.67, M+23 559.0, prep 70% methanol/8 ml/min.

EBC-148: RRT 0.91, M+23 573.2, prep 67% methanol/8 ml/min.

EBC-158: RRT 0.78, M+23 603.2, semi-synthetic.

EBC-161: RRT 1.15, M+23=587.2, semi-synthetic.

EBC-167: RRT 1.47, M+23=601.5, semi-synthetic.

EBC-170: RRT 1.12, M+23 597.2, prep 73% methanol, followed by the Halo RP-Amide column in acetonitrile/water at 2 ml/min.

EBC-172: RRT 1.24, M+23=619.4, semi-synthetic.

EBC-177: RRT 2.07, M+23 653.4, prep 85% methanol/5 ml/min.

EBC-186: RRT 0.27, M+23 503.0, prep 60% methanol/8 ml/min.

EBC-188: RRT 0.92, M+23=601.5, semi-synthetic.

EBC-211: RRT 0.96, M+23=585.5, semi-synthetic.

EBC-344: RRT 1.05, M+23 585.4, prep 73% methanol, followed by the Halo RP-Amide column and then on a Phenomenex Luna 3 µm C18(2) 100Å, 250 mm × 4.60 mm column, in 52% acetonitrile.

Characterization

Physicochemical and spectral data of isolated compounds (See Supplementary Information Data File S1 for ^{1}H and ^{13}C NMR characterization tables).

EBC-46 was isolated as a colourless oil; $[\alpha]^{25.3}_{D}$ –9.1° (c 0.21, $CDCl_3$); IR cm^{-1}: 3399, 3061, 2967, 2928, 2880, 1708, 1651, 1634, 1456, 1380, 1328, 1253, 1153, 1134, 1075, 1023, 977; HRESIMS $[M+Na]^+$ m/z 585.2669 (calcd for $C_{30}H_{42}O_{10}Na$ – 585.2670).

EBC-47 was isolated as a colourless oil; $[\alpha]^{27.1}_{D}$ 39.1° (c 0.107, $CDCl_3$); IR cm^{-1}: 3383, 3058, 2961, 2926, 2878, 1729, 1712, 1694, 1647, 1630, 1459, 1378, 1266, 1238, 1188, 1155, 1077, 1019, 1012; HRESIMS $[M+Na]^+$ m/z 587.2826 (calcd for $C_{30}H_{42}O_{10}Na$ – 587.2827).

EBC-59 was isolated as a colourless oil.

EBC-83 was isolated as a colourless oil; $[\alpha]^{27.9}_{D}$ –14.4° (c 0.47 , $CDCl_3$); IR cm^{-1}: 3408, 3060, 2958, 2927, 2874, 1737, 1713, 1705, 1627, 1459, 1378, 1326, 1241, 1163, 1083, 1025, 986, 934, 823, 807; HRESIMS $[M+Na]^+$ m/z 601.3010 (calcd for $C_{31}H_{46}O_{10}Na$ – 601.2983).

EBC-146 was isolated as a colourless oil; $[\alpha]^{27.5}_{D}$ 18.0° (c 0.371, $CDCl_3$); IR cm^{-1}: 3385, 3112, 3013, 2965, 2918, 2881, 2808, 2687, 2635, 1743, 1583, 1557, 1495, 1458, 1437, 1423, 1400, 1245, 1141, 1096, 1057, 955, 900, 879; HRESIMS $[M+Na]^+$ m/z 545.2362 (calcd for $C_{27}H_{38}O_{10}Na$ – 545.2357).

EBC-147 was isolated as a colourless oil; $[\alpha]^{27.5}_{D}$ –3.7° (c 0.409, $CDCl_3$); IR cm^{-1}: 3402, 3128, 2968, 2927, 2881, 2635, 1739, 1707, 1690, 1583, 1494, 1459, 1437, 1430, 1423, 1400, 1245, 1192, 1141, 1082, 1058, 1008, 955, 900, 878; HRESIMS $[M+Na]^+$ m/z 559.2516 (calcd for $C_{28}H_{40}O_{10}Na$ – 559.2514).

EBC-148 was isolated as a colourless oil; $[\alpha]^{27.5}_{D}$ –5.5° (c 0.126, $CDCl_3$); IR cm^{-1}: 3405, 3114, 3007, 2966, 2926, 2881, 1740, 1712, 1691, 1583, 1494, 1460, 1437, 1423, 1400, 1247, 1141, 1057, 900, 879; HRESIMS $[M+Na]^+$ m/z 573.2677 (calcd for $C_{29}H_{42}O_{10}Na$ – 573.2670).

EBC-158 was isolated as a colourless oil; $[\alpha]^{28.2}_{D}$ –4.6° (c 0.373, $CDCl_3$); IR cm^{-1}: 3375, 3062, 2958, 2925, 2876, 2857, 1742, 1706, 1690, 1650, 1629, 1459, 1379, 1330, 1257, 1192, 1151, 1136, 1075, 1020, 978; HRESIMS $[M+Na]^+$ m/z 603.2793 (calcd for $C_{30}H_{44}O_{11}Na$ – 603.2776).

EBC-161 was isolated as a colourless oil; $[\alpha]^{27.7}_{D}$ 51.0° (c 0.908, $CDCl_3$); IR cm^{-1}: 3433, 3332, 3067, 2966, 2926, 2878, 1728, 1706, 1627, 1459, 1378, 1266, 1238, 1188, 1152, 1088, 1073, 1060, 1011, 914; HRESIMS $[M+Na]^+$ m/z 587.2820 (calcd for $C_{30}H_{44}O_{10}Na$ – 587.2827).

EBC-167 was isolated as a colourless oil; $[\alpha]^{27.9}_{D}$ 37.1° (c 0.249, $CDCl_3$); IR cm^{-1}: 3401, 3334, 3074, 2958, 2926, 2872, 1737, 1707, 1633, 1457, 1378, 1242, 1165, 1092, 1058, 1011, 919; HRESIMS $[M+Na]^+$ m/z 601.2996 (calcd for $C_{31}H_{46}O_{10}Na$ –601.2983).

EBC-170 was isolated as a colourless oil; HRESIMS $[M+Na]^+$ m/z 597.2662 (calcd for $C_{31}H_{42}O_{10}Na$ – 597.2670).

EBC-172 was isolated as a colourless oil; $[\alpha]^{26.0}_{D}$ 26.3° (c 0.07, $CDCl_3$); IR cm^{-1}: 3750, 3424, 3369, 2961, 2927, 2875, 2860, 1739, 1713, 1692, 1631, 1546, 1455, 1378, 1338, 1244, 1166, 1083, 1031, 1010, 931, 802; HRESIMS $[M+Na]^+$ m/z 619.3076 (calcd for $C_{31}H_{48}O_{11}Na$ – 619.3089).

EBC-177 was isolated as a colourless oil; $[\alpha]^{23.4}_{D}$ –20.6° (c 0.034, $CDCl_3$); IR cm^{-1}: 3407, 3068, 2956, 2923, 2855, 1715, 1701, 1641, 1459, 1378, 1247, 1164, 1081, 1050, 1028, 803; HRESIMS $[M+Na]^+$ m/z 653.3312 (calcd for $C_{35}H_{50}O_{10}Na$ – 653.3296).

EBC-186 was isolated as a colourless oil; $[\alpha]^{25.7}_{D}$ 55.6° (c 0.073, $CDCl_3$); IR cm^{-1}: 3399, 2954, 2923, 2854, 1713, 1700, 1459, 1377, 1157, 1083, 1024, 802; HRESIMS $[M+Na]^+$ m/z (calcd for $C_{25}H_{36}O_9Na$ – 503.2252).

EBC-188 was isolated as a colourless oil; $[\alpha]^{21.5}_{D}$ 25.3° (c 0.035, $CDCl_3$); HRESIMS $[M+Na]^+$ m/z 605.2948 (calcd for $C_{30}H_{46}O_{11}Na$ – 605.2932).

EBC-211 was isolated as a colourless oil; $[\alpha]^{27.9}_{D}$ 16.8° (c 0.04, $CDCl_3$); IR cm^{-1}: 3350, 2956, 2924, 2855, 1746, 1702, 1650, 1457, 1377, 1260, 1155, 1078, 1033, 1016; HRESIMS $[M+Na]^+$ m/z 585.2680 (calcd for $C_{30}H_{42}O_{10}Na$ – 585.2670).

EBC-344 was isolated as a colourless oil; HRESIMS $[M-H]^-$ m/z 561.2700 (calcd for $C_{30}H_{41}O_{10}$ – 561.2705).

Semi-synthesis

EBC-158: TT (EBC-46) (2.0 g, 3.6 mmol) was dissolved in a 1:1 mixture of ethanol and dilute aqueous sulfuric acid (0.6 M, 400 ml) and heated at 60°C for 16 h. After removal of ethanol and extraction with ethyl acetate the product was fractionated by HPLC on a Phenomenex Luna 5 µm C18(2) 100Å, 250 mm × 21.2 mm column, flow rate 8.0 ml/min, with 67% methanol in water (isocratic). Relevant fractions were acidified (0.01 % formic acid) and evaporated at a maximum temperature of 50°C to give EBC-158 (415 mg) as a colorless solid in 20% yield.

EBC-161: EBC-47 (25 mg, 0.0444 mmol) was dissolved in a 1:1 mixture of ethanol and phosphate buffer (0.067 M, pH 6.5) (20 ml) and heated at 60°C for 9 days. After removal of ethanol and extraction with ethyl acetate the product was fractionated by HPLC on a Phenomenex Luna 5 µm C18(2) 100Å, 250 mm × 21.2 mm column, flow rate 10.0 ml/min with 77% methanol in water, giving EBC-161 (4.67 mg) as a pale yellow oil in 19% yield. An analytical sample was obtained by further purification on a Phenomenex Luna 3 µm C18(2) 100Å, 250 mm × 4.60 mm column, flow rate 1.0 ml/min with 52% acetonitrile in water (isocratic).

EBC-167: EBC-83 (25 mg, 0.043 mmol) was dissolved in a 1:1 mixture of ethanol and phosphate buffer (0.067 M, pH 6.5) (20 ml) and heated at 60°C for 9 days. After removal of ethanol and extraction with ethyl acetate the product was fractionated by HPLC on a Phenomenex Luna 5 µm C18(2) 100Å, 250 mm × 21.2 mm column, flow rate 10.0 ml/min, 77% methanol in water (isocratic) giving EBC-167 (8.18 mg) as a pale yellow oil in 33% yield.

EBC-172: EBC-83 (32.94 mg, 0.057 mmol) was dissolved in a 1:1 mixture of ethanol and dilute aqueous sulfuric acid (0.6 M, 30 ml) and heated at 60°C for 16 h. After removal of ethanol and extraction with ethyl acetate the product was fractionated by HPLC on a Phenomenex Luna 5 µm C18(2) 100Å, 250 mm × 21.2 mm column, flow rate 8.0 ml/min with 70% methanol in water mobile phase (isocratic). Relevant fractions were acidified (0.01% formic acid) and evaporated at 50°C, giving EBC-172 (6.02 mg) as a pale yellow oil in 18% yield. An analytical sample was obtained by further purification on a Phenomenex Luna 3 µm C18(2) 100Å, 250 mm × 4.60 mm column, flow rate 1.0 ml/min with 52% acetonitrile in water mobile phase (isocratic).

EBC-188: EBC-47 (28.56 mg, 0.051 mmol) was dissolved in a 1:1 mixture of ethanol and dilute aqueous sulfuric acid (0.6 M, 20 ml) and heated at 60°C for 16 h. After removal of ethanol and extraction with ethyl acetate the product was fractionated by HPLC on a Phenomenex Luna 5 µm C18(2) 100Å, 250 mm × 21.2 mm column, flow rate 8.0 ml/min with 70% methanol in water mobile phase (isocratic). Relevant fractions were acidified (0.01% formic acid) before evaporation at 50°C, giving EBC-188 (4.10 mg) as a pale yellow oil in 14% yield. An analytical sample was obtained by further purification on a Phenomenex Luna 3 µm C18(2) 100Å, 250 mm × 4.60 mm column, flow rate 1.0 ml/min with 52% acetonitrile in water mobile phase (isocratic).

EBC-211: TT (EBC-46) (2.5 g, 4.4 mmol) was dissolved in a 1:1 mixture of ethanol and phosphate buffer (0.067 M, pH 6.5) (500 ml) and heated at 60°C for 5 days. After removal of ethanol and extraction with ethyl acetate the product was fractionated by HPLC on a Phenomenex Luna 5µm C18(2) 100Å, 250 mm × 21.2 mm column, flow rate 8.0 ml/min with 67% methanol in water mobile phase (isocratic). Impure EBC-211 fractions were pooled and further purified under the same conditions. EBC-211 fractions were acidified (0.01% formic acid) and dried by rotary evaporation at a maximum temperature of 50°C to yield EBC-211 (394 mg) as a pale yellow oil in16% yield.

Treatment of TT (EBC-46) under Appel conditions (crystallization for x-ray analysis): To a mixture of TT (EBC-46) (10 mg, 17.8 µmol) and triphenylphosphine (18.6 mg, 71.1 µmol) in anhydrous carbon tetrachloride under an argon atmosphere was added diisopropylethylamine (49.5 µl, 0.28 mmol). The reaction mixture was then gradually heated to 80°C with stirring, and then heating continued at this temperature overnight. After 14 h the solvent was removed *in vacuo* and the crude mixture purified by consecutive (×2) silica gel column chromatography (diethyl ether and diethyl ether:petroleum ether (1:1) respectively) to afford the title compound as an amorphous solid (2.8 mg) in 19% yield, which was crystallized from ethanol to afford white crystals. ^{1}H NMR (749 MHz, $CDCl_3$) □ 0.27 (3 H, s), 0.89 (3 H, t, *J*=7.5 Hz), 0.93 (3 H, d, *J*=6.6 Hz), 1.06 (3 H, s), 1.08 (3 H, d, *J*=6.9 Hz), 1.17 (1 H, d, *J*=6.7 Hz), 1.40 (1 H, ddq, *J*=14.0, 7.0, 6.8 Hz), 1.49 (3 H, dd, *J*=3.0, 1.4 Hz), 1.61 (1 H, dq, *J*=10.2, 6.6 Hz), 1.68 (1 H, ddq, *J*=14.0, 7.3, 7.0 Hz), 1.80 (3 H, dq, *J*=1.4, 1.1 Hz), 1.84 (3 H, dq, *J*=7.1, 1.1 Hz), 2.26 (1 H, d, *J*=6.7 Hz), 2.32 (1 H, sxt., *J*=7.0 Hz), 3.15 (1 H, d, *J*=1.1 Hz), 3.54 (1 H, d, *J*=11.7 Hz), 4.29 (1 H, m, *J*=2.0 Hz), 4.37 (1 H, d, *J*=11.7 Hz), 4.99 (1 H, dd, *J*=16.2 on P, 1.3 Hz), 5.37 (1 H, d, *J*=10.2 Hz), 6.72 (1 H, qq, *J*=7.1, 1.4 Hz), 7.17 (6 H, m, *J*=7.7, 3.6 Hz), 7.25 (3 H, m, *J*=1.6 Hz), 7.42 (6 H, m, *J*=12.0, 8.5, 1.2 Hz), 7.45 (1 H, bs); ^{13}C NMR (126 MHz, $CDCl_3$) □ C4 was not resolved by 1D or 2D, 10.01 (1 C, s), 11.56 (1 C, s), 12.29 (1 C, s), 14.45 (1 C, s), 14.84 (1 C, s), 16.13 (1 C, s), 16.72 (1 C, s), 23.88 (1

C, s), 26.13 (1 C, s), 26.50 (1 C, s), 36.53 (1 C, s), 36.63 (1 C, s), 41.06 (1 C, s), 46.34 (1 C, s), 48.35 (1 C, d, *J*=8.5 Hz), 48.74 (1 C, s), 61.72 (1 C, d, *J*=3.6 Hz), 65.58 (1 C, s), 66.40 (1 C, s), 67.20 (1 C, d, *J*=3.1 Hz), 76.66 (1 C, s), 76.76 (1 C, br. s.), 127.06 (6 C, d, *J*=12.6 Hz), 128.54 (1 C, s), 129.03 (3 C, d, *J*=2.7 Hz), 133.55 (1 C, s), 134.60 (6 C, d, *J*=9.4 Hz), 137.32 (1 C, s), 144.88 (3 C, d, *J*=118.9 Hz), 158.06 (1 C, s), 167.22 (1 C, s), 178.65 (1 C, s), 203.29 (1 C, s); HRESIMS $[M+Na]^+$ m/z 863.3098 (calcd for $C_{48}H_{54}ClO_9Na$ – 863.3086).

Protection of tigliane-12,13-diesters (**2**) as acetonides (**3**): To a solution of pyridinium-*p*-toluenesulfonate (PPTS, 4.1 g, excess) in *N*,*N*-dimethylformamide (DMF, 50 ml), a solution of the tigliane 12,13-diesters (8.2 g) in *N*,*N*-dimethylformamide (10 ml) was added. After stirring at room temperature for 2 minutes, 2,2-dimethoxypropane (DMP, 120 ml) was added, and stirring was continued for 24 h at room temperature. The reaction was eventually worked up by dilution with brine (150 ml) and extraction with EtOAc (50 ml). The organic phase was washed with brine (2 × 150 ml), dried (Na_2SO_4), filtered and evaporated, and the residue was purified by gravity column chromatography on silica gel (PE/EtOAc 8:2→6:4 as eluent) to afford the acetonides (5.2 g, 3.5%) as a gummy yellowish solid.

Acetonides (**3**) deacylation: A sodium methoxide solution (NaOMe, 0.21 M) was freshly prepared by slowly adding, under stirring, small pieces of sodium (9.7 g) to methanol (HPLC grade, 2 l). To this solution (128 ml) was added the above acetonide mixture (6.4 g) under vigorous stirring. The pH of the reaction mixture was maintained in the range of 11.5-12.0 by judicious addition of NaOMe (0.21 M), taking care not to overshoot pH = 12.5. The pH was controlled with pH strips for the range 0.3 – 2.3. After stirring at room temperature for 24 hours, the reaction was worked up by neutralization with acetic acid, filtration and evaporated to ca. 1/20 of the original volume. EtOAc (20 ml) was added to this solution, and washed with 2 N H_2SO_4 (100 ml). The acidic aqueous phase was counter-extracted with EtOAc, and the combined EtOAc extracts were washed with brine (2 × 300 ml), dried (Na_2SO_4), filtered and evaporated. The residue was purified by gravity column chromatography on silica gel (PE/EtOAc 6:4→4:6 as eluent) to afford 1.4 g of a white powder.

EBC-47-acetonide: To a solution of deacyl-EBC-46 acetonide (100 mg, 0.23 mmol) in THF (5 ml), 4-*N*,*N*-dimethylaminopyridine (DMAP) (15 mg, 0.12 mmol) was added, and the solution was heated to 60°C (oil bath temperature). Separately, to a solution of (*S*)-2-methylbutyric acid (163 µl, 1.50 mmol) in THF (10 ml/g), *N*,*N'*-dicyclohexylcarbodiimide (DCC, 310 mg, 1.50 mmol) was added. After stirring for ~15 minutes the suspension was filtered through a wad of cotton wool, and added dropwise to the THF

solution of deacyl-EBC46 acetonide. After stirring for 24 hours at 60°C, the reaction was worked up by dilution with EtOAc (~200 ml) and washing with H_2SO_4 (2 M, ~50 ml), brine (2 × ~50 ml), and next with sat. $NaHCO_3$ (~50 ml) and brine (2 × ~50 ml). After drying (Na_2SO_4), filtration and evaporation, the residue was purified by gravity column chromatography on silica gel (PE/EtOAc 9:1→6:4 as eluent) to afford the titled compound (111 mg, 80%) as a white powder.

Semi-synthesis of EBC-47 (deprotection): The acetonide diester (100 mg) was added to a freshly prepared solution of trifluoroacetic acid (TFA) in dichloromethane (2% v/v; 200 µl, 2 µl/mg). After stirring for 6-12 h at room temperature, the reaction was worked up by washing with a mixture of sat. $NaHCO_3$ (~10 ml) and brine (~40 ml), and next with brine alone (2 × ~40 ml). After drying (Na_2SO_4), filtration and evaporation, the residue was purified by gravity column chromatography on silica gel (PE/EtOAc 8:2→2:8 as eluent) to afford the TT (EBC-46) analogues (yield ca: 60-70%).

Log P determination

Log P analysis was performed isocratically on a Halo RP-amide column: 4.6 mm x 150 mm x 2.7 µm, P/N: 92814-707, flow rate 1.0 ml/min in either 55% acetonitrile or 65% acetonitrile/water for the more hydrophobic compounds. The log P of tiglianes were determined from standard curves of the retention times of diterpene esters of known log P.

Cell growth/survival assays

K562 cells (3×10^3 cells per well) were seeded into U-bottom 96-well plates (Corning #3799) in 90 µl of media. Compounds were prepared via serial dilution in media (to 10× final assay concentration), and 10 µl of compound / vehicle dilutions were subsequently added to cells in duplicate. Vehicle (ethanol) only controls were also prepared. Treated cells were then incubated in a humidified incubator at 37°C, 5% CO_2 for 6 days, before assessment of growth/viability using the CellTiter 96® AQ_{ueous} One Solution Cell Proliferation Assay kit (Promega) according to the manufacturer's instructions. Absorbance values at 490 nm were recorded using a H4 Hybrid Synergy plate reader (Biotek). A media only control was also measured for background subtraction. Modified absorbance values from compound treated wells were normalized to vehicle treated samples and the % growth/survival in each sample determined. Percentage growth/survival was plotted against Log_{10}[Compound] nM to generate absolute IC_{50} curves for each compound using Prism v8.2.1 (GraphPad).

For adherent cell based assays, cells were plated into clear 96-well plates (Corning #3595) in 100 µl of media at 3×10^3 cells per well. After 24 h, 2 methodologies were used to treat cells. With MCF7, media was removed and 90 µl of fresh media inserted into each well. Compounds were then prepared and delivered as detailed above for K562 based assays. For the remaining cell lines, media was replaced with 100 µl media containing compound in duplicate over the concentration range 0-500 µM. Typically, plates were incubated at 37°C, 5% CO_2 for 3-7 days dependent on cell type. Cell growth/survival in the adherent cell context was determined using a sulforhodamine B (SRB) assay.

Supplementary Information Data File S1 - NMR Tables

^{1}H and ^{13}C NMR characterization tables

^{1}H and ^{13}C NMR data for TT (EBC-46) recorded at 500/125 MHz in $CDCl_3$.

Position	^{1}H, δ (ppm)	multiplicity	*J*, Hz	^{13}C, δ (ppm)
1	7.71	dd	(2.5, 1.5)	164.7
2				133.4
3				209.9
4				72.4
5	4.22	s		71.3
6				61.8
7	3.26	s		65.2
8	3.17	d	(6.9)	36.0
9				77.2
10	4.06	dq	(2.6, 2.6)	48.9
11	1.94	m		45.9
12	5.42	d	(9.8)	76.7
13				65.5
14	1.26	d	(6.9)	36.1
15				26.6
16	1.21	s		17.2
17	1.24	s		23.6
18	0.84	d	(6.4)	15.1
19	1.73	dd	(2.9, 1.5)	9.7
20	3.82	AB	(12.2)	64.6
1'				167.4
2'				128.4
3'	6.79	qq	(6.9, 1.5)	137.6
4'	1.77	dq	(7.1, 1.2)	14.4
5'	1.80	dq	(1.5, 1.5)	12.2
1"				178.9
2"	2.37	sxt	(6.9)	41.2
3"	1.43	m		26.1
3"	1.71	m		
4"	0.92	t	7.3	11.6
5"	1.11	d	6.8	16.1
9-OH	6.02	br. s.		

^{1}H and ^{13}C NMR data for EBC-47 recorded in $CDCl_3$.

Position	^{1}H, δ (ppm)	multiplicity	J, Hz	^{13}C, δ (ppm)
1	7.71	dd	(2.4, 1.5)	164.7
2				133.5
3				209.9
4				72.4
5	4.22	s		71.5
6				61.7
7	3.26	s		65.2
8	3.16	d	(6.8)	36.0
9				77.2
10	4.06	t	(2.7)	48.9
11	1.9	dd	(10.0, 6.6)	45.5
12	5.4	d	(10.3)	76.2
13				65.5
14	1.26	d	(6.8)	36.1
15				26.5
16	1.22	s		23.7
17	1.21	s		17.2
18	0.85	d	(6.4)	15.0
19	1.74	dd	(2.7, 1.2)	9.7
20	3.85	d	(12.2)	64.5
20	3.78	m	(12.7)	
1'				175.9
2'	2.36	q	(7.0)	41.8
3'	1.44	td	(13.9, 7.3)	26.7
3'	1.63	dd	(7.8, 5.9)	
4'	0.91	t	(7.8)	11.6
5'	1.12	d	(6.8)	17.0
1"				178.8
2"	2.36	q	(7.0)	41.2
3"	1.44	td	(13.9, 7.3)	26.2
3"	1.69	dd	(13.9, 7.1)	
4"	0.89	d	(7.3	11.6
5"	1.11	d	(6.8)	16.1
9-OH	5.98	m		
OH	3.61	m		

^{1}H and ^{13}C NMR data for EBC-59 recorded in $CDCl_3$.

Position	^{1}H, δ (ppm)	multiplicity	*J*, Hz	^{13}C, δ (ppm)
1	7.72	dd	(2.4, 1.3)	164.8
2				133.5
3				210.0
4				72.4
5	4.22	s		71.6
6				61.6
7	3.27	s		65.3
8	3.17	d	(6.7)	36.0
9				76.7
10	4.06	d	(2.7)	48.9
11	1.95	dq		45.9
12	5.41	d		77.1
13				65.5
14	1.27	m		36.2
15				26.7
16	1.24	s		17.2
17	1.22	s		23.6
18	0.86	d	(7.0)	15.1
19	1.74	dd	(2.8, 1.2)	9.7
20	3.86	m		64.5
20	3.78	m		
1'				166.6
2'	5.79	d	(15.2)	119.5
3'	7.23	dd	(15.5, 10.9)	145.3
4'	6.19	dd	(14.8, 11.2)	127.5
5'	6.51	dd	(14.9, 10.7)	141.7
6'	6.11	dd	(15.1, 10.7)	129.7
7'	5.92	dt	(15.2, 7.2)	141.1
8'	2.12	q		33.0
9'	1.39	m		28.6
10'	1.26	m		31.4
11'	1.29	m		22.5
12'	0.87	m	(7.0)	14.0
1"				179.0
2"	2.38	sxt	(7.0)	41.2
3"	1.71	m		26.2
3"	1.45	dd	(14.1, 7.0)	
4"	0.92	t	7.5)	11.6
5"	1.12	d	(7.0)	16.2
4-OH	3.57	s		
OH	6.04	br. s		

^1H and ^{13}C NMR data for EBC-83 recorded in $CDCl_3$.

Position	^1H, δ (ppm)	multiplicity	*J*, Hz	^{13}C, δ (ppm)
1	7.7	dd	(2.4, 1.3)	164.6
2				133.4
3				209.9
4				72.4
5	4.22	s		71.4
6				61.8
7	3.25	s		65.2
8	3.14	d	(6.6)	35.96
9				77.1
10	4.06	t	(2.6)	48.9
11	1.9	dq	10.0, 6.5)	45.6
12	5.37	d	(10.0)	76.5
13				65.5
14	1.25	d	(6.6)	36.04
15				26.6
16	1.21	s		17.1
17	1.21	s		23.6
18	0.84	d	(6.5)	15.0
19	1.74	dd	(2.9, 1.3)	9.7
20	3.79	dd	(12.5, 5.7)	64.6
20	3.83	dd	(12.5, 7.9)	
1'				173.3
2'	2.27	dt	(7.4, 3.7)	34.5
3'	1.6	quin	(7.4)	24.9
4'	1.29	m	(7.5, 7.2, 3.9)	31.1
5'	1.29	m	(7.5, 7.2, 3.9)	22.3
6'	0.87	t	(7.0)	13.9
1"				178.8
2"	2.36	sxt	(7.0)	41.2
3"	1.7	ddd	(13.9, 7.3, 7.1)	26.2
3"	1.43	m	(14.1, 7.4, 7.1)	
4"	0.91	t	(7.5)	11.6
5"	1.11	d	(7.0)	16.1
4-OH	3.64	br. s.		
5-OH	3.94	d	(2.5)	
9-OH	5.95	br. s.		

^{1}H and ^{13}C NMR data for EBC-146 recorded in $CDCl_3$.

Position	^{1}H, δ (ppm)	multiplicity	*J*, Hz	^{13}C, δ (ppm)
1	7.7	s		164.6
2				133.5
3				209.9
4				72.4
5	4.21	s		71.5
6				61.7
7	3.26	s		65.2
8	3.14	d	(6.8)	36.0
9				76.8
10	4.04	t	(2.7)	48.9
11	1.91	m		45.7
12	5.33	d	(9.8)	77.1
13				65.4
14	1.25	d	(6.8)	36.1
15				26.7
16	1.21	s		17.1
17	1.23	s		23.7
18	0.85	d	(6.4)	15.1
19	1.75	dd	(2.9, 1.0)	9.7
20	3.78	d	(12.7)	64.5
20	3.85	d	(12.7)	
1'				170.6
2'	2.04	s		21
1"				178.9
2"	2.36	m	(7.0, 6.8)	41.2
3"	1.44	m	(14.1, 7.3, 7.2)	26.2
3"	1.71	dd		
4"	0.92	t	(7.6)	11.6
5"	1.12	d	(6.8)	16.2
4-OH	3.58	br. s		
9-OH	5.97	br. s		

^{1}H and ^{13}C NMR data for EBC-147 recorded in $CDCl_3$.

Position	^{1}H, δ (ppm)	multiplicity	*J*, Hz	^{13}C, δ (ppm)
1	7.71	m		164.6
2				133.5
3				209.9
4				72.4
5	4.21	s		71.5
6				61.7
7	3.26	s		65.2
8	3.15	d	(6.8)	36.0
9				77.1
10	4.05	m		48.9
11	1.91	m		45.7
12	5.35	d	(9.8)	76.8
13				65.4
14	1.25	dd	(10.3, 6.8)	36.1
15				26.7
16	1.21	s		17.1
17	1.22	s		23.7
18	0.85	d	(6.8)	15.1
19	1.74	dd	(2.9, 1.5)	9.7
20	3.78	d	(12.2)	64.6
20	3.84	d	(12.7)	
1'				173.9
2'	2.31	m		27.8
3'	1.13	t		9.3
1"				178.9
2"	2.37	dd	(13.7, 6.8)	41.2
3"	1.44	m	(14.0, 7.0, 6.6)	26.2
3"	1.7	dd	(14.2, 6.8)	
4"	0.92	t	(7.6)	11.6
5"	1.12	d	(7.3)	16.1
9-OH	5.92	br. s.		

^{1}H and ^{13}C NMR data for EBC-148 recorded in $CDCl_3$.

Position	^{1}H, δ (ppm)	multiplicity	*J*, Hz	^{13}C, δ (ppm)
1	7.71	dd	(2.4, 1.0)	164.7
2				133.5
3				209.9
4				72.3
5	4.21	s		71.7
6				61.6
7	3.27	s		65.2
8	3.15	d	(6.4)	36.0
9				77.1
10	4.05	d	(2.4)	48.9
11	1.90	dd	(10.0, 6.6)	45.6
12	5.38	d	(9.8)	76.6
13				65.5
14	1.26	d	(6.8)	36.1
15				26.6
16	1.22	s		17.1
17	1.23	s		23.7
18	0.85	d	(6.4)	15.1
19	1.75	d	(2.9)	9.7
20	3.77	m	(12.2)	64.5
20	3.86	m	(12.7)	
1'				173.1
2'	2.27	m		36.4
3'	1.64	m	(14.6, 7.2, 7.1)	18.7
4'	0.94	t	(7.3)	13.5
1"				178.8
2"	2.37	qt	(7.0, 6.8	41.2
3"	1.45	dq	(14.0, 7.1)	26.2
3"	1.71	dd	(13.7, 6.8)	
4"	0.92	t	(7.6)	11.6
5"	1.12	d		16.1
4-OH	3.54	br. s		
5-OH	3.83	br. s		
9-OH	5.97	br. s		

^{1}H and ^{13}C NMR data for EBC-158 recorded in $CDCl_3$.

Position	^{1}H, δ (ppm)	multiplicity	J, Hz	^{13}C, δ (ppm)
1	7.71	s		162.9
2				134.4
3				209.5
4				74.6
5	4.08	bs		72.2
6				76.6
7	4.08	bs		82.5
8	2.90	d	(6.4)	36.7
9				77.8
10	3.55	m		52.4
11	2.09	m	(10.0, 6.5, 6.4)	44.6
12	5.40	d	(9.8)	76.4
13				66.0
14	1.15	d	(6.4)	35.7
15				26.6
16	1.25	s		23.5
17	1.22	s		17.0
18	0.87	d	(6.8)	14.8
19	1.78	s		9.8
20	3.79	d	(11.2)	68.9
20	3.67	d	(10.8)	
1'				167.5
2'				128.3
3'	6.80	m	(7.1, 6.8, 1.5)	137.8
4'	1.77	s		14.4
5'	1.80	s		12.2
1"				179.8
2"	2.38	q	(6.8)	41.2
3"	1.71	dd	(14.2, 6.8)	26.1
3"	1.45	m	(14.1, 7.6, 7.2)	
4"	0.93	t	(7.6)	11.6
5"	1.13	d	(7.3)	16.2
4-OH	3.55	bs		
5-OH	4.97	s		
6-OH	4.52	d	(2.4)	
7-OH	3.31	s		
9-OH	6.54	br. s.		
20-OH	3.11	m	(10.3)	

^{1}H and ^{13}C NMR data for EBC-161 recorded in $CDCl_3$.

Position	^{1}H, δ (ppm)	multiplicity	J, Hz	^{13}C, δ (ppm)
1	7.59	s		160.0
2				134.6
3				206.3
4				71.3
5	3.85	d	1.0	62.2
6				67.6
7	4.37	d	5.3	77.2
8	2.33	d	5.4	35.1
9				79.1
10	3.54	dd	2.4, 2.2	57.2
11	2.10	dd	10.2, 6.5	43.3
12	5.36	d	10.3	75.4
13				65.6
14	1.18	d	5.7	34.6
15				26.0
16	1.17	s		16.9
17	1.19	s		23.5
18	0.84	d	6.6	13.9
19	1.80	dd	2.8, 1.3	10.3
20	3.98	m	12.8	63.2
20	3.83	d	13.0	
1'				175.9
2'	2.35	m		41.7
3'	1.62	dt	8.2, 6.9	26.7
3'	1.42	m		
4'	0.89	m	7.6	11.6
5'	1.12	d	7.0	16.8
1"				179.6
2"	2.38	d	4.3	41.3
3"	1.68	d	7.1	26.2
3"	1.46	dt	6.8, 3.3	
4"	0.91	d	7.5	11.6
5"	1.11	d	7.0	16.1
4-OH	3.96	s		
7-OH	5.03	d	5.5	
9-OH	6.46	s		
20-OH	3.14	br. s.		

^1H and ^{13}C NMR data for EBC-167 recorded in $CDCl_3$.

Position	^1H, δ (ppm)	multiplicity	*J*, Hz	^{13}C, δ (ppm)
1	7.58	d	(1.5)	159.8
2				134.6
3				206.0
4				71.3
5	3.78	d	(1.1)	62.5
6				67.1
7	4.42	d	(3.9)	77.2
8	2.3	d	(3.8)	35.2
9				79.1
10	3.53	t	(2.5)	57.3
11	2.08	dq	(10.3, 6.5)	43.3
12	5.34	d	(10.3)	75.6
13				65.6
14	1.19	s		34.6
15				26.1
16	1.18	s		16.8
17	1.21	s		23.5
18	0.83	d		13.9
19	1.81	dd	(2.8, 1.3)	10.3
20	3.94	d	(12.5)	64.0
20	3.82	d	(12.6)	
1'				173.3
2'	2.28	m		34.5
3'	1.61	m		24.9
4'	1.29	m		31.1
5'	1.30	m	(7.6, 7.3, 3.6)	22.3
6'	0.88	t	(6.9)	13.9
1"				179.6
2"	2.37	q	(7.0)	41.3
3"	1.70	m	(14.1, 7.3, 7.1)	26.2
3"	1.44	dt	(14.1, 7.0)	
4"	0.92	t	(7.5)	11.6
5"	1.12	d	(7.1)	16.1
4-OH	3.35	br. s.		
7-OH	5.06	d	(5.4)	
9-OH	6.48	s		
20-OH	2.73	m		

^{1}H and ^{13}C NMR data for EBC-170 recorded in $CDCl_3$.

Position	^{1}H, δ (ppm)	multiplicity	*J*, Hz	^{13}C, δ (ppm)
1	7.72	dd	(2.2, 1.3)	164.8
2				133.5
3				210.0
4				72.4
5	4.21	br. s.		71.7
6				61.6
7	3.28	s		65.3
8	3.18	d	(6.7)	36.1
9				77.1
10	4.06	br. s.		49.0
11	1.96	dd	(9.8, 6.2)	45.9
12	5.44	d	(10.1)	76.8
13				65.5
14	1.27	m		36.2
15				26.7
16	1.23	br. s.		23.7
17	1.26	s		17.2
18	0.87	d	(6.5)	15.2
19	1.75	dd	(2.8, 1.2)	9.7
20	3.87	m		64.5
20	3.78	m		
1'				166.6
2'	5.83	d	(15.0)	120.7
3'	7.59	ddd	(15.3, 11.7, 1.2)	139.6
4'	6.14	m		127.3
5'	5.94	m		136.2
6'	1.86	dd	(7.3, 1.7)	14.1
1"				179.0
2"	2.37	m		41.2
3"	1.70	m		26.2
3"	1.45	s		
4"	0.93	t		11.6
5"	1.13	m	(6.8)	16.2

^{1}H and ^{13}C NMR data for EBC-172 recorded in $CDCl_3$.

Position	^{1}H, δ (ppm)	multiplicity	*J*, Hz	^{13}C, δ (ppm)
1	7.71	dd	(2.4, 1.5)	162.9
2				134.4
3				209.6
4				74.6
5	4.08	s		72.2
6				76.6
7	4.07	d	(2.4)	82.5
8	2.89	d	(6.4)	36.7
9				77.8
10	3.54	d	(2.4)	52.3
11	2.05	dq	(10.1, 6.6)	44.3
12	5.35	d	(9.8)	76.2
13	3.27	s		65.9
14	1.14	d	(6.4)	35.6
15				26.6
16	1.22	s		16.9
17	1.22	s		23.6
18	0.87	d	(6.4)	14.8
19	1.79	dd	(2.9, 1.5)	9.8
20	3.79	d	(11.2)	68.9
20	3.67	m	(11.2)	
1'				173.4
2'	2.29	td	(7.3, 3.9)	34.5
3'	1.60	dd	(15.2, 7.3)	24.9
4'	1.28	m		31.1
5'	1.3	m		22.3
6'	0.88	t	(7.1)	13.9
1"				179.7
2"	2.38	d	(7.3)	41.3
3"	1.71	dt	(14.7, 13.7, 7.3)	26.2
3"	1.44	td	(14.1, 7.1)	
4"	0.92	t	(7.6)	11.6
5"	1.13	d	(7.3)	16.2
4-OH	3.46	s		
5-OH	4.94	s		
7-OH	4.48	d	(2.0)	
9-OH	6.49	br. s.		
20-OH	3.09	d	(11.2)	

^{1}H and ^{13}C NMR data for EBC-177 recorded in $CDCl_3$.

Position	^{1}H, δ (ppm)	multiplicity	*J*, Hz	^{13}C, δ (ppm)
1	7.72	dq	(2.5, 1.3)	164.8
2				133.5
3				210.0
4				72.3
5	4.22	m		71.7
6				61.6
7	3.27	s		65.3
8	3.17	d	(6.6)	36.1
9				77.1
10	4.05	m		49.0
11	1.95	dq	(9.7, 6.4)	45.9
12	5.41	d	(9.9)	76.7
13				65.5
14	1.26	m		36.2
15				26.7
16	1.22	s		23.6
17	1.24	s		17.2
18	0.86	d	(5.6)	15.1
19	1.75	dd	(2.9, 1.3)	9.7
20	3.87	m		64.5
20	3.78	dd	(12.0, 4.6)	
1'				166.6
2'	5.75	d	(15.4)	118.8
3'	7.20	dd	(15.5, 9.9)	145.6
4'	6.16	s		128.3
5'	6.13	dd	(6.7, 6.2)	145.3
6'	2.15	m		33.0
7'	1.41	m		28.4
8'	1.26	br. s.		31.3
9'	1.29	m		22.4
10'	0.87	d	(11.7)	14.0
1"				178.9
2"	2.38	m		41.2
3"	1.73	m		26.2
3"	1.45	m		
4"	0.93	t	(7.5)	11.6
5"	1.12	d	(7.0)	16.2
OH	3.55	m		

^{1}H and ^{13}C NMR data for EBC-186 recorded in $CDCl_3$.

Position	^{1}H, δ (ppm)	multiplicity	J, Hz	^{13}C, δ (ppm)
1	7.70	dd	(2.4, 1.5)	163.8
2				133.9
3				209.7
4				72.1
5	4.20	s		71.5
6				62.4
7	3.27	s		66.0
8	3.08	d	(7.3)	36.5
9				77.6
10	3.79	d	(2.9)	50.7
11	1.76	d	(15.7)	47.3
12	3.90	d	(9.8)	78.3
13				68.4
14	1.24	m		34.8
15				27.9
16	1.20	s		23.4
17	1.26	s		17.2
18	1.07	d	(6.4)	16.2
19	1.77	dd	(2.7, 1.2)	9.8
20	3.81	m		65.0
1'				180.1
2'	2.41	m	(7.0, 6.8)	41.0
3'	1.71	dt	(13.7, 7.3)	26.5
3'	1.45	ddd	(13.8, 7.1, 7.0)	
4'	0.92	t	(7.6)	11.7
5'	1.16	d	(6.8)	16.6

^{1}H and ^{13}C NMR data for EBC-188 recorded in $CDCl_3$.

Position	^{1}H, δ (ppm)	multiplicity	*J*, Hz	^{13}C, δ (ppm)
1	7.71	dd	(2.3, 1.4)	162.9
2				134.4
3				209.5
4				74.6
5	4.08	s		72.2
6				76.6
7	4.07	d	(2.2)	82.5
8	2.90	d	(6.2)	36.7
9				77.9
10	3.55	d	(2.6)	52.4
11	2.05	m		44.2
12	5.38	d	(10.0)	75.9
13				65.9
14	1.15	d	(6.2)	35.6
15				26.5
16	1.23	s		23.6
17	1.22	s		16.9
18	0.88	d	(6.6)	14.7
19	1.78	dd	(2.7, 1.3)	9.8
20	3.79	d	(11.3)	69.0
20	3.67	m	(11.0)	
1'				176.0
2'	2.35	m		41.8
3'	1.46	m		26.7
3'	1.64	m		
4'	0.90	t	(7.5)	11.6
5'	1.12	d	(7.1)	17.0
1"				180.3
2"	2.38	d	(6.9)	41.3
3"	1.70	ddd	(13.9, 7.3, 7.1)	26.2
3"	1.46	m		
4"	0.92	t	(7.5)	11.6
5"	1.12	d	(7.1)	16.1
4-OH	3.47	s		
5-OH	4.95	s		
6-OH	3.27	s		
7-OH	4.48	d	(2.2)	
9-OH	6.49	m		
20-OH	3.07	d	(10.6)	

^{1}H and ^{13}C NMR data for EBC-211 recorded at 188/750 MHz in $CDCl_3$.

Position	^{1}H, δ (ppm)	multiplicity	*J*, Hz	^{13}C, δ (ppm)
1	7.59	dd	(2.0, 1.5)	159.9
2				134.6
3				206.1
4				71.3
5	3.84	d	(1.1)	62.3
6				67.5
7	4.39	d	(5.5)	77.2
8	2.33	d	(5.7)	35.2
9				79.1
10	3.55	t	(2.6)	57.2
11	2.13	q	(2.9)	43.7
12	5.39	d	(10.2)	75.9
13				65.7
14	1.19	s		34.7
15				26.1
16	1.2	s		17.0
17	1.21	s		23.5
18	0.84	d	(6.6)	14
19	1.81	dd	(2.9, 1.5)	10.3
20	3.98	dd	(12.8, 7.3)	63.5
20	3.83	dd	(12.8, 4.9)	
1'				167.4
2'				128.3
3'	6.8	dd	(7.1, 1.5)	137.7
4'	1.77	dd	(7.1, 1.1)	14.4
5'	1.8	t	(1.30	12.2
1"				179.7
2"	2.38	q	7	41.2
3"	1.72	dq		26.1
3"	1.44	dt	(14.1, 7.0)	
4"	0.92	t	(7.5)	11.6
5"	1.12	d	(6.9)	16.2
4-OH	3.7	br. s.		
7-OH	5.06	d	(5.5)	
9-OH	6.53	br. s.		
20-OH	2.99	br. s.		

^1H and ^{13}C NMR data for EBC-344 recorded at 226/900 MHz in $CDCl_3$.

Position	^1H, δ (ppm)	multiplicity	J, Hz	^{13}C, δ (ppm)
1	7.72	dd	2.4, 1.2	164.9
2				133.4
3				210.0
4				72.4
5	4.21	s	-	71.7
6				61.6
7	3.27	s	-	65.3
8	3.15	d	6.7	36.1
9				77.1
10	4.05	d	2.4	49.0
11	1.91	m	-	45.8
12	5.41	d	9.9	75.5
13				65.6
14	1.25	br. s	-	36.0
15				26.5
16	1.22	br. s	-	17.1
17	1.22	br. s	-	23.7
18	0.85	d	5.7	15.0
19	1.75	dd	2.8, 1.2	9.8
20	3.77	d	12.5	64.5
20	3.86	d	12.5	
1'				166.0
2'	5.62	m	1.2	115.8
3'				157.2
4'	2.13	d	1.2	20.3
5'	1.88	d	1.2	27.4
1"				178.8
2"	2.37	m	-	41.3
3"	1.45	m	-	26.3
3"	1.72	dd	13.8, 7.5	
4"	0.92	t	7.5	11.6
5"	1.12	d	7.0	16.2

Supplementary Information Data File S2- Spectra

S3.1 ^{1}H NMR spectrum for EBC-46 recorded in $CDCl_3$.

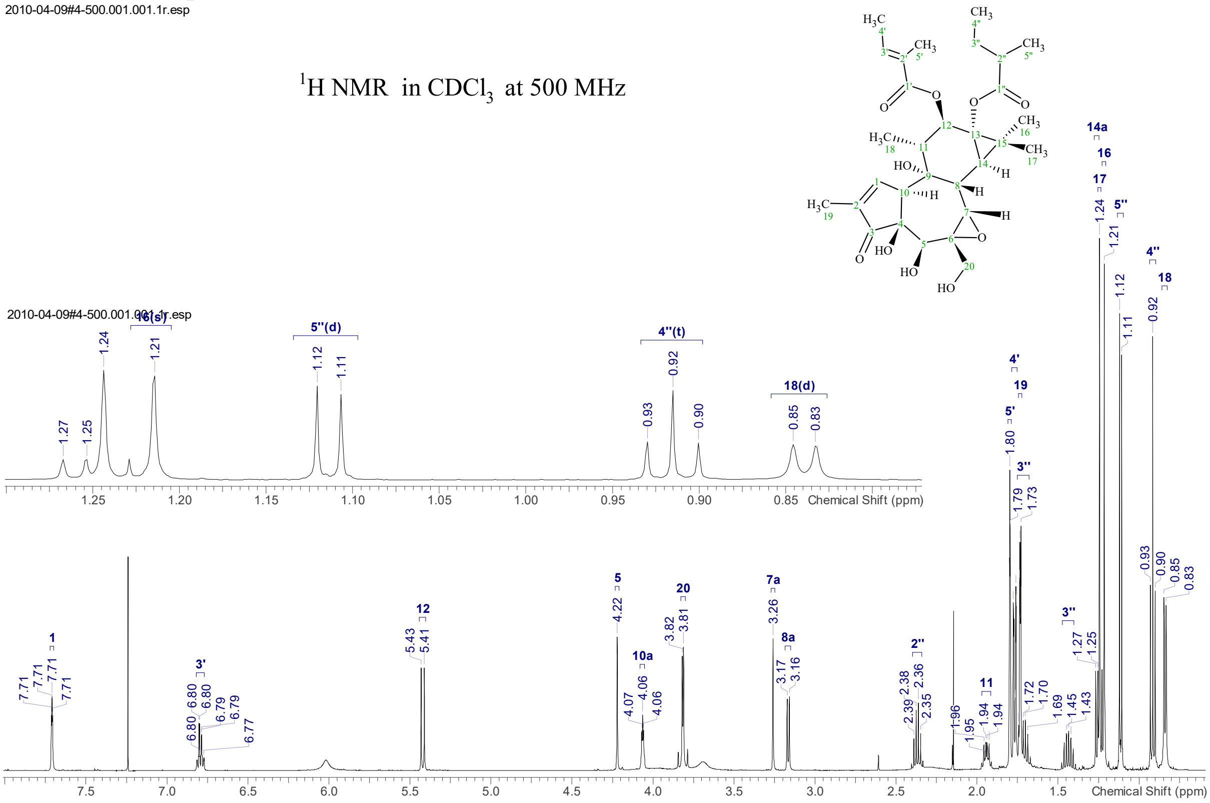

S3.2 ^{13}C NMR spectrum for EBC-46 recorded in $CDCl_3$.

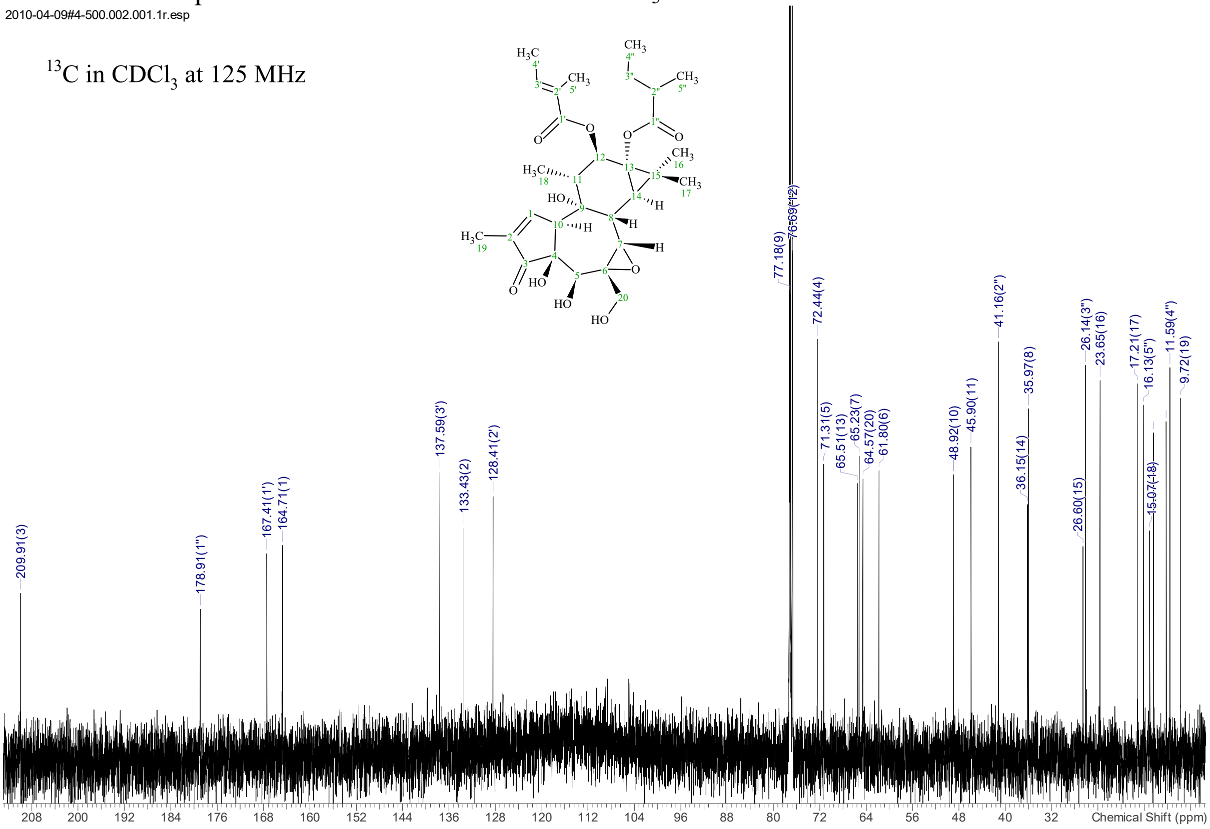

S3.3 ^{13}C NMR spectrum 5-80 ppm expansion for EBC-46 recorded in $CDCl_3$.

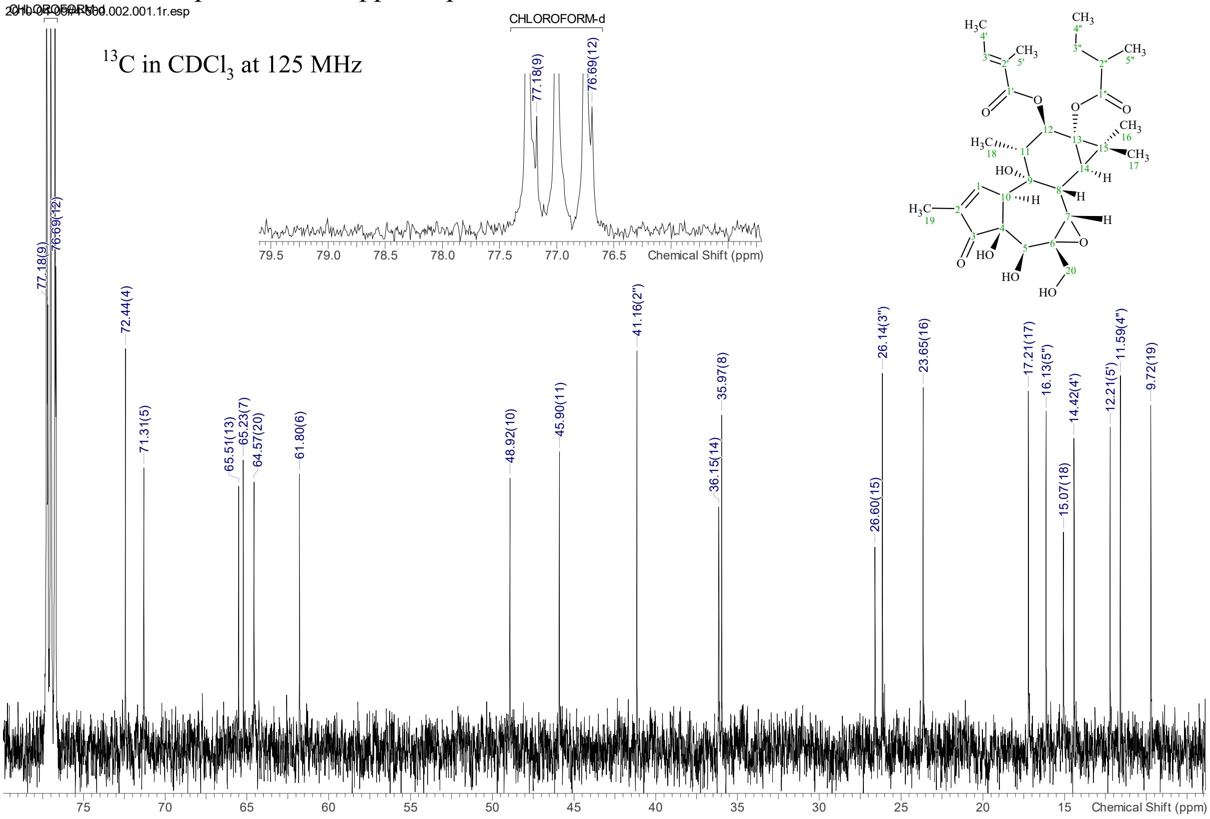

S3.4 DEPT spectrum for EBC-46 recorded in $CDCl_3$.

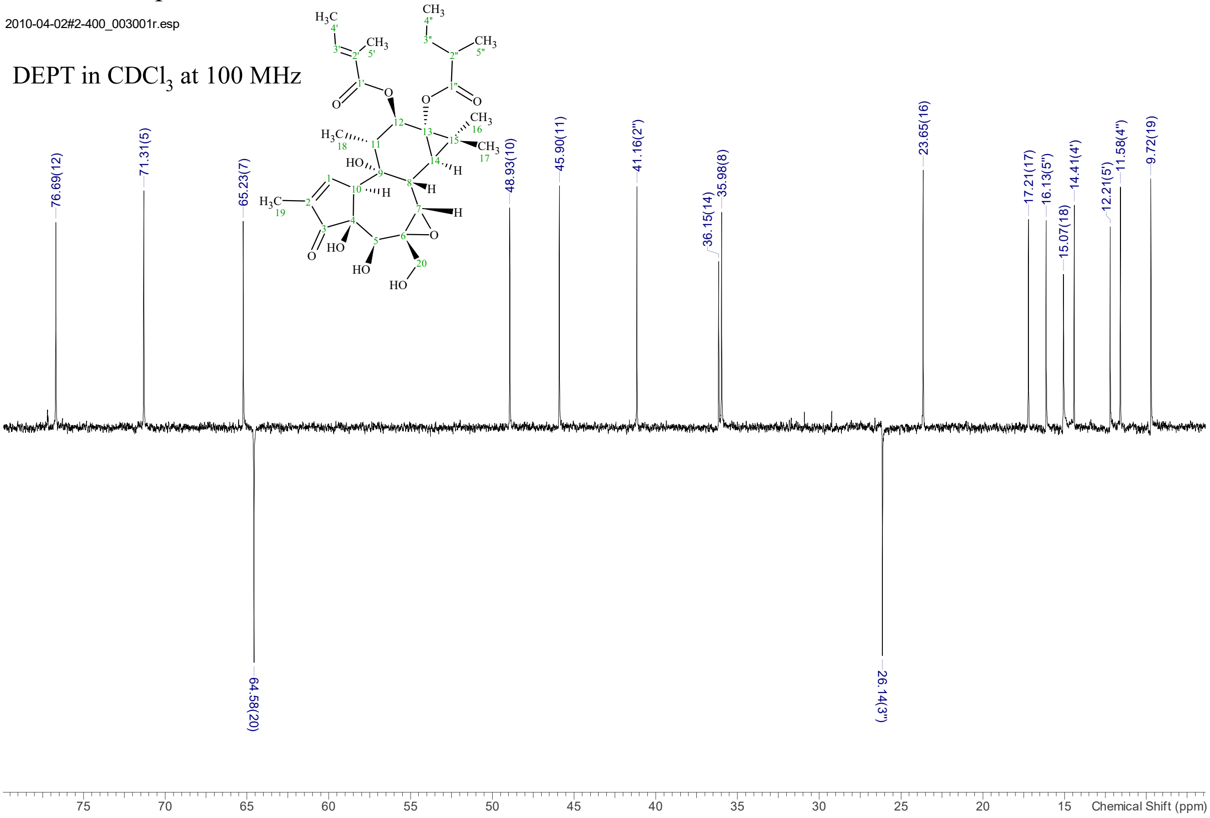

S3.5 DEPT spectrum 5-80 ppm expansion for EBC-46 recorded in $CDCl_3$.

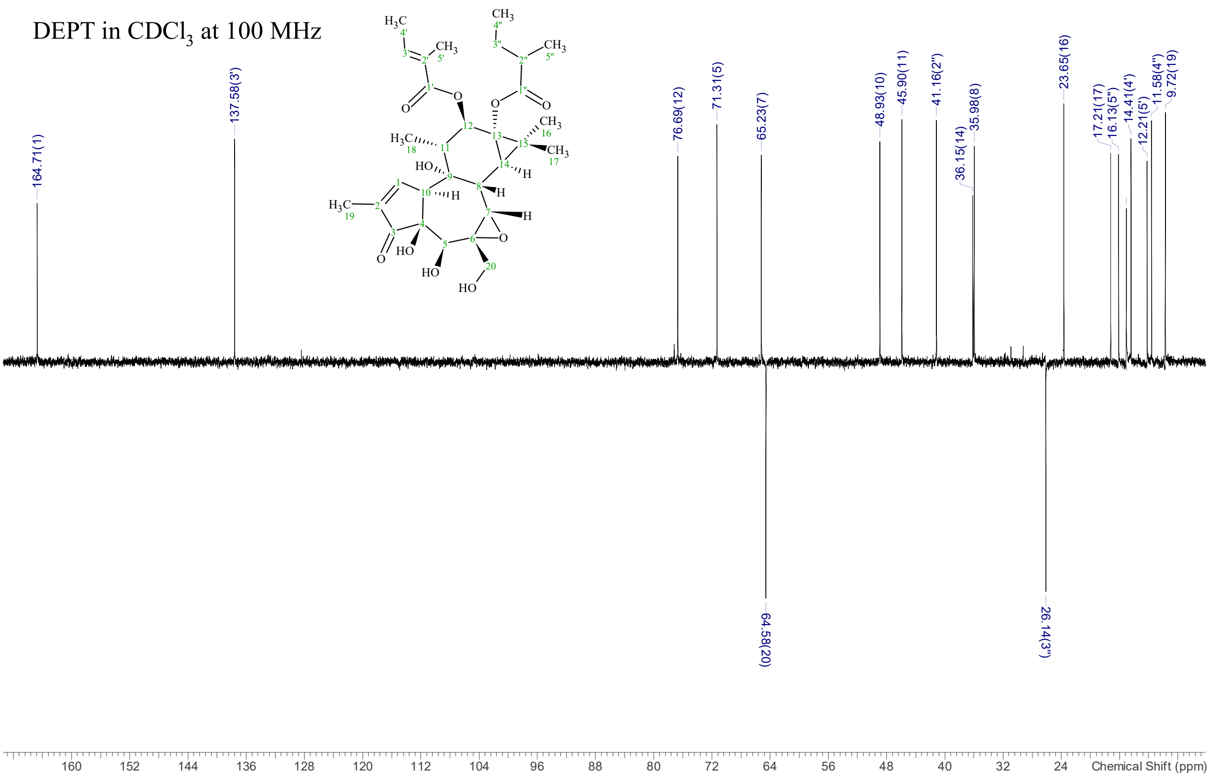

S3.6 COSY spectrum for EBC-46 recorded in $CDCl_3$.

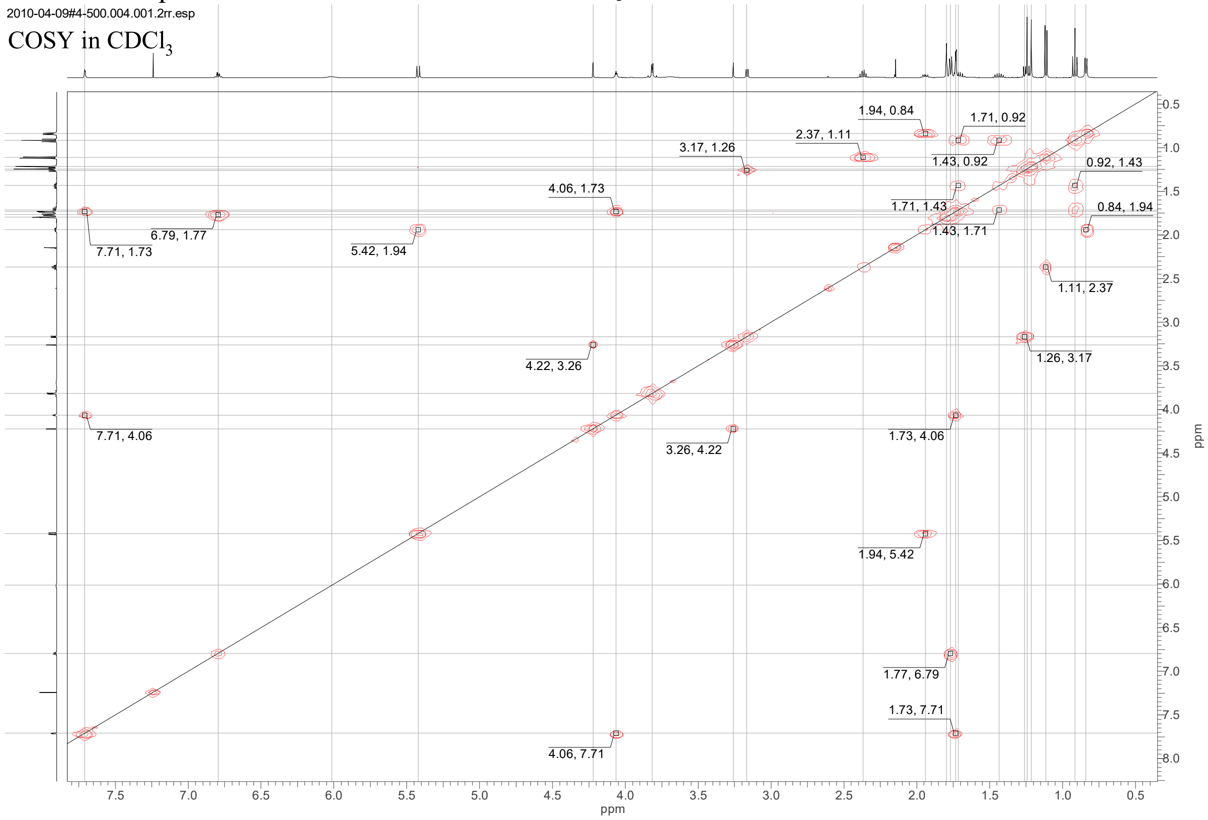

S3.7 COSY correlation diagrams for EBC-46 for spectra recorded in $CDCl_3$.

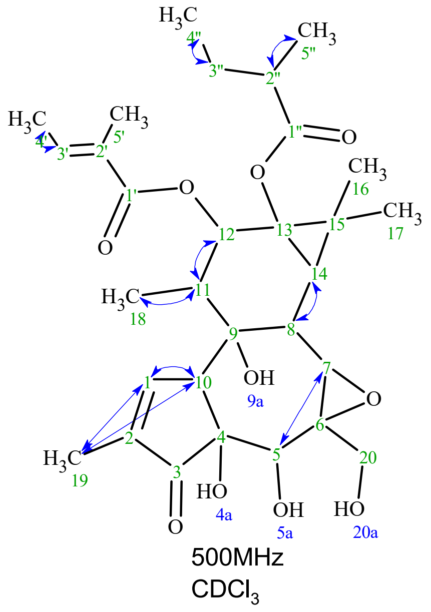

S3.8 HSQC spectrum for EBC-46 recorded in $CDCl_3$.

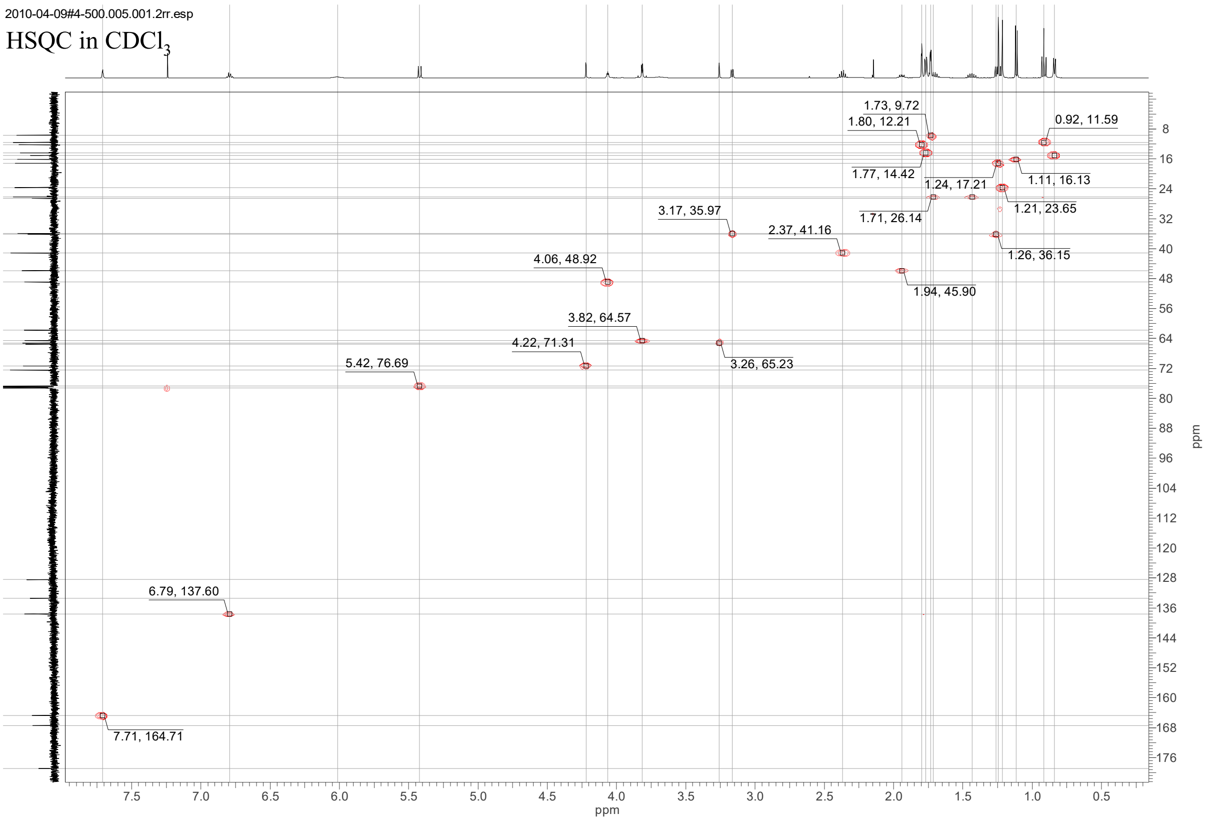

S3.9 HMBC spectrum and 0.7-1.9×5-185 ppm expansion for EBC-46 recorded in $CDCl_3$.

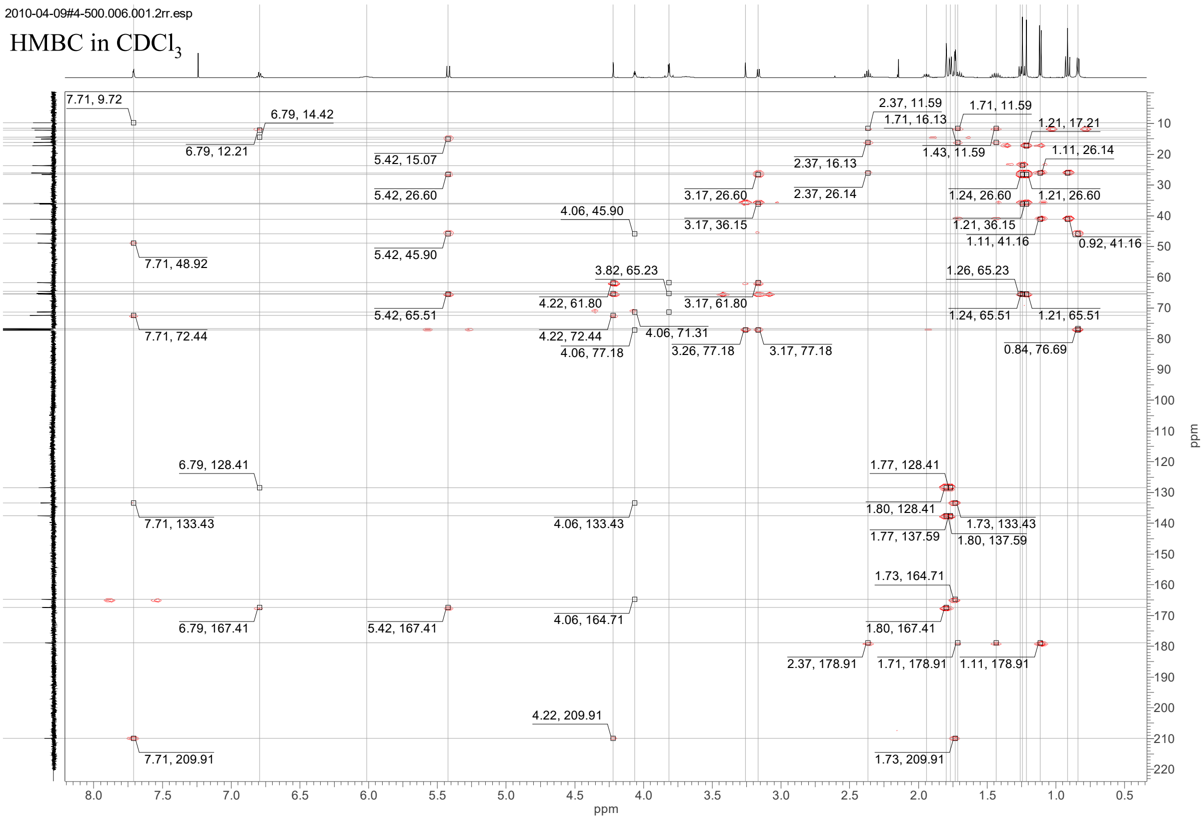

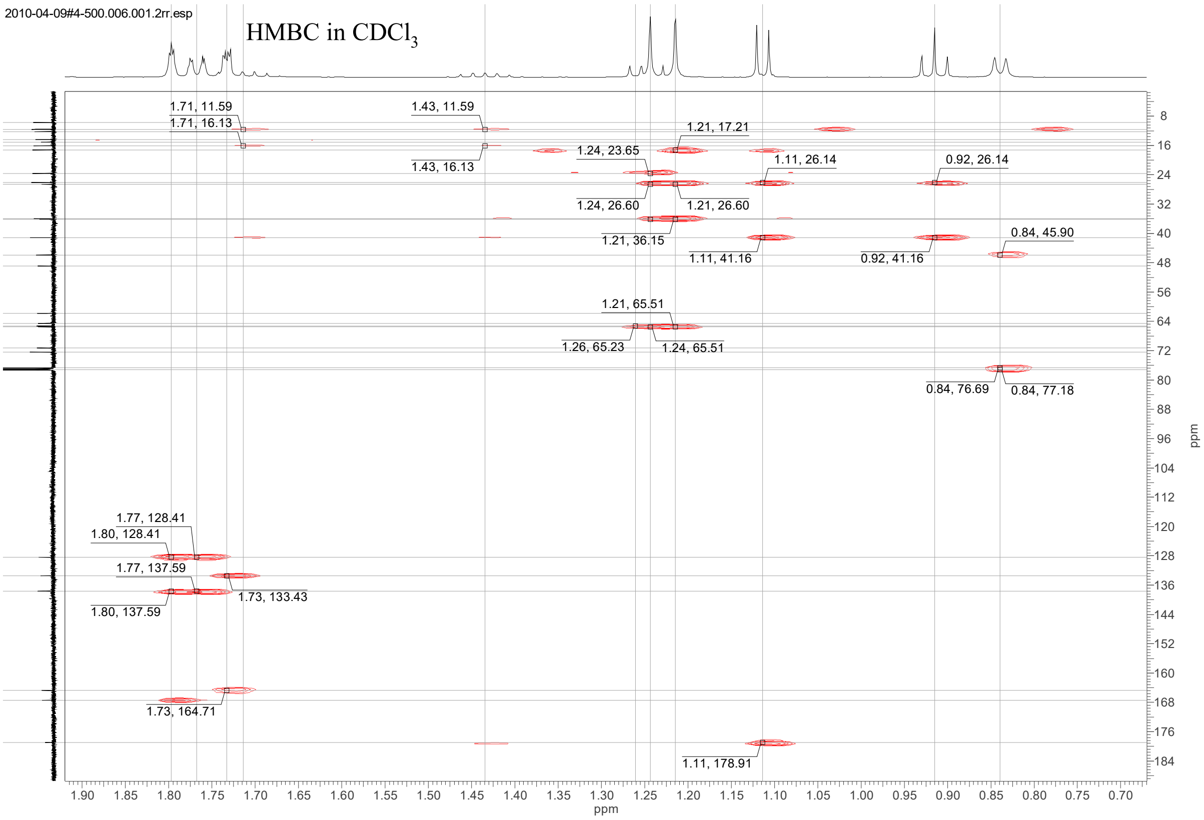

S3.10 HMBC correlation diagrams for EBC-46 for spectra recorded in $CDCl_3$.

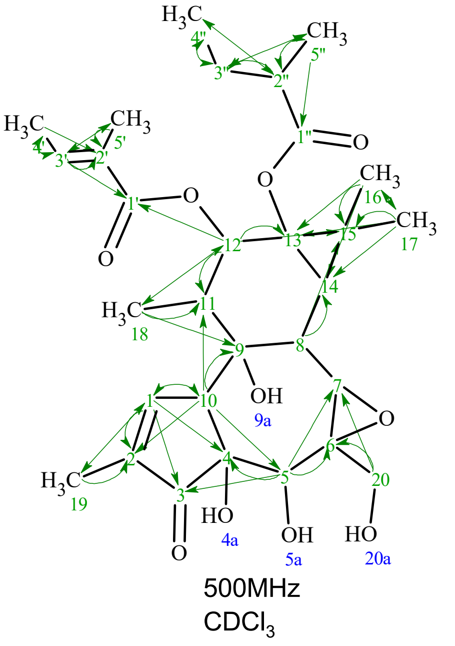

S3.11 NOE difference spectra for EBC-46 recorded in $CDCl_3$.

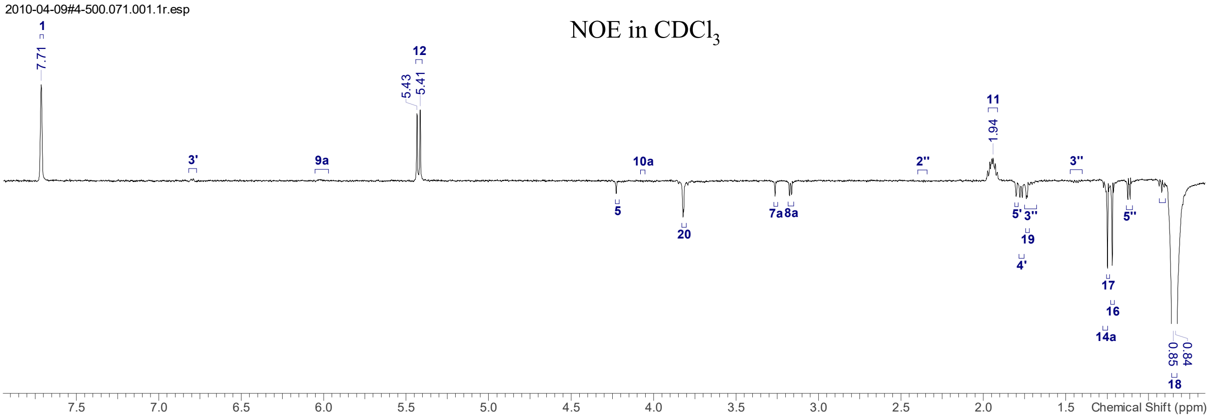

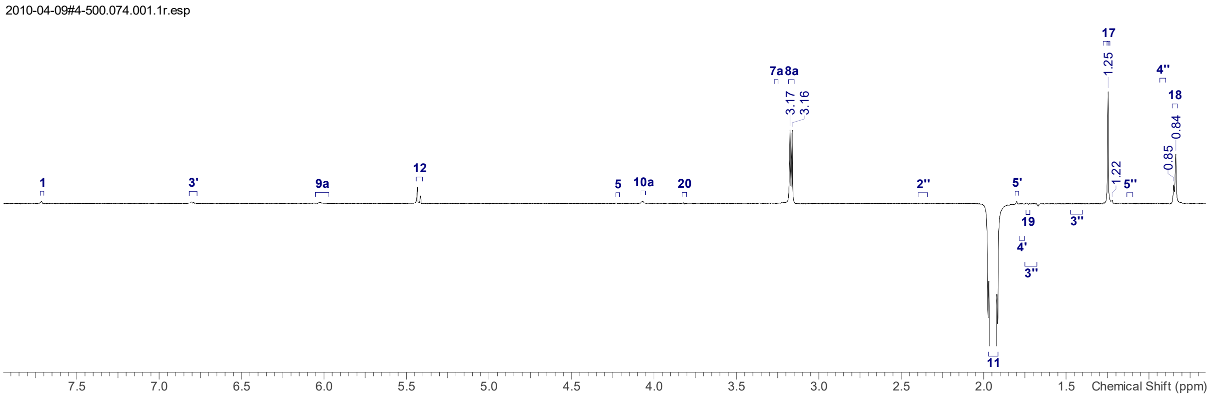

2010-04-09#4-500.076.001.1r.esp

NOE in $CDCl_3$

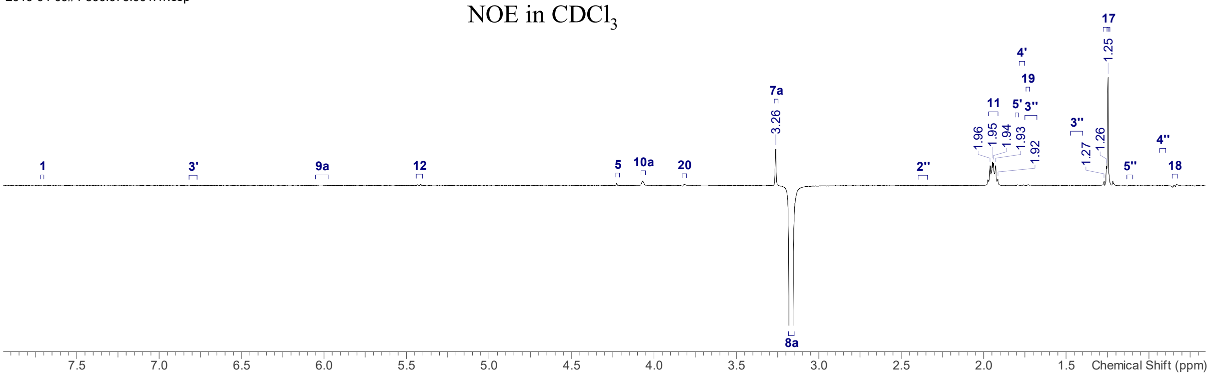

2010-04-09#4-500.077.001.1r.esp

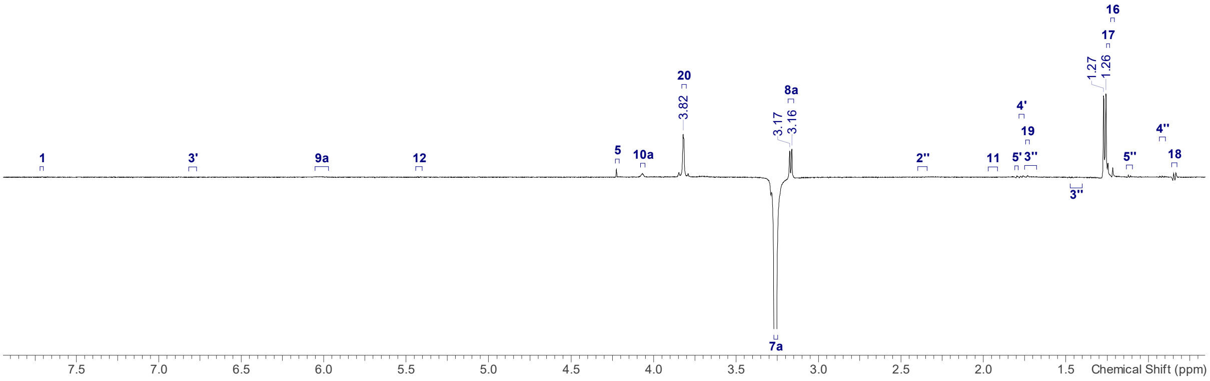

2010-04-09#4-500.078.001.1r.esp

NOE in $CDCl_3$

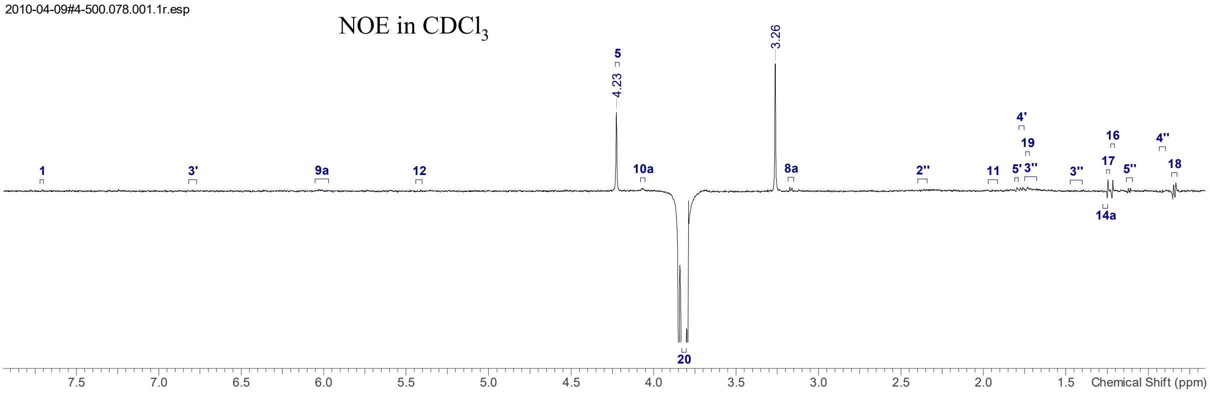

2010-04-09#4-500.079.001.1r.esp

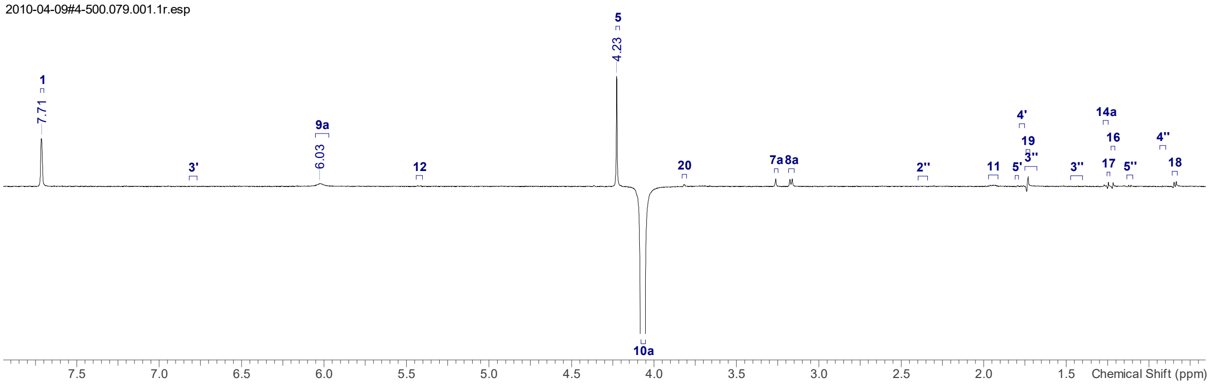

2010-04-09#4-500.080.001.1r.esp

NOE in $CDCl_3$

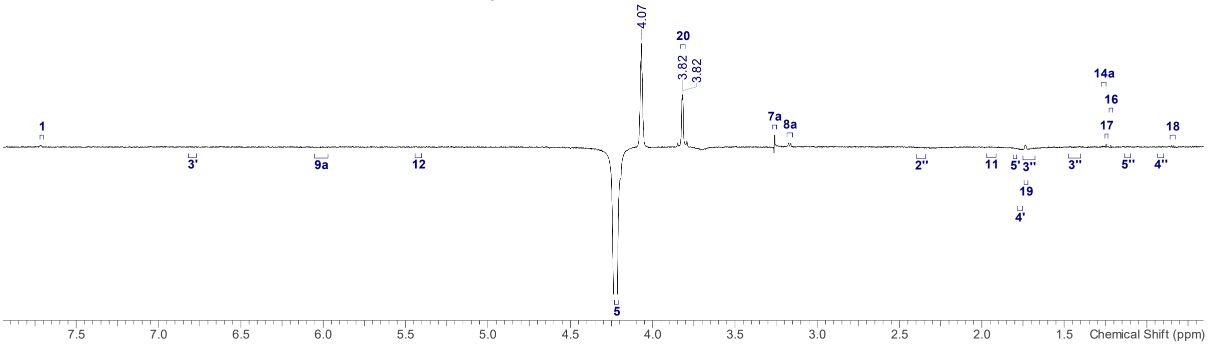

2010-04-09#4-500.081.001.1r.esp

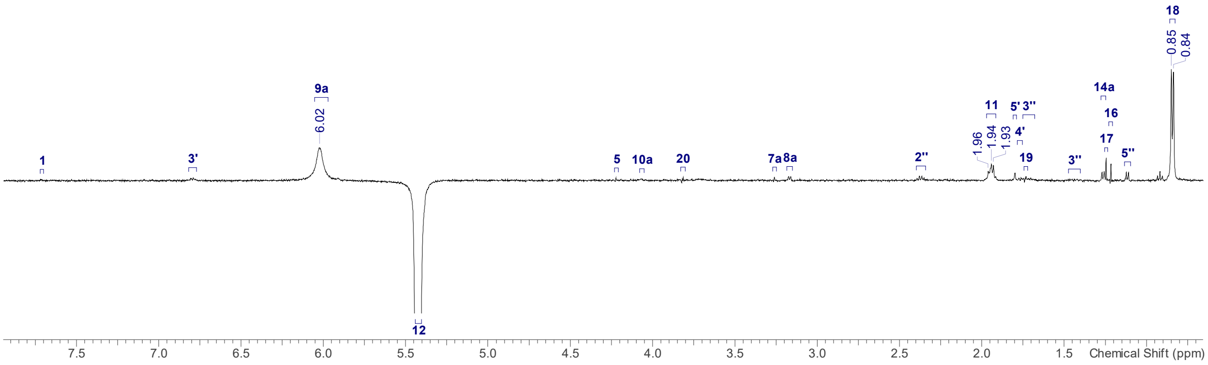

2010-04-09#4-500.083.001.1r.esp

NOE in $CDCl_3$

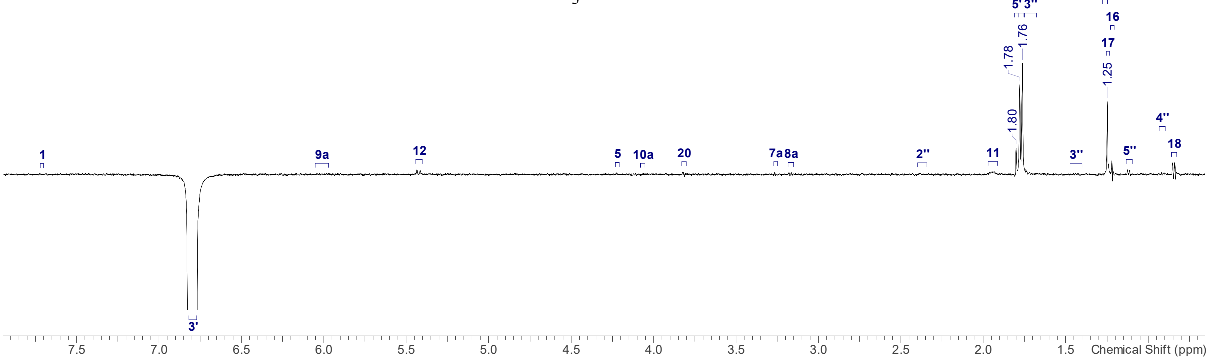

2010-04-09#4-500.084.001.1r.esp

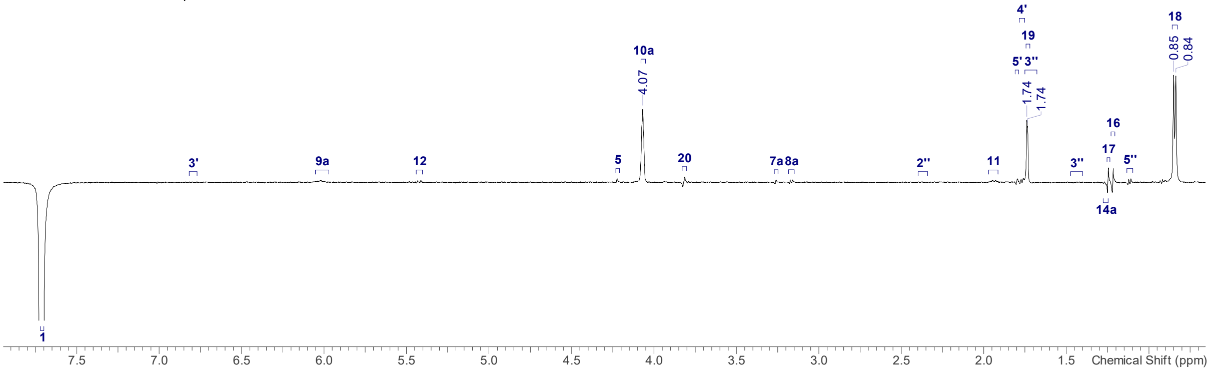

S3.12 NOESY spectrum and its 0.7-3.7×0.5-4.1 ppm expansion for EBC-46 recorded in $CDCl_3$

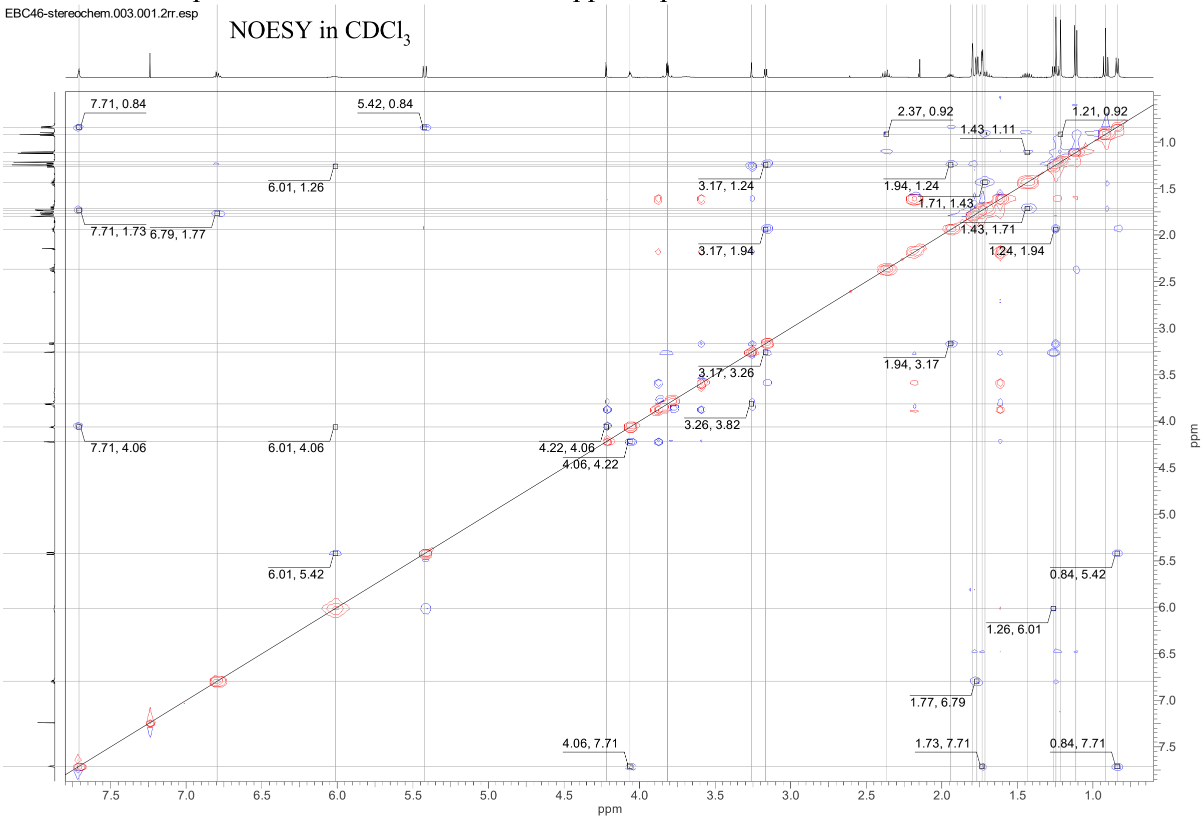

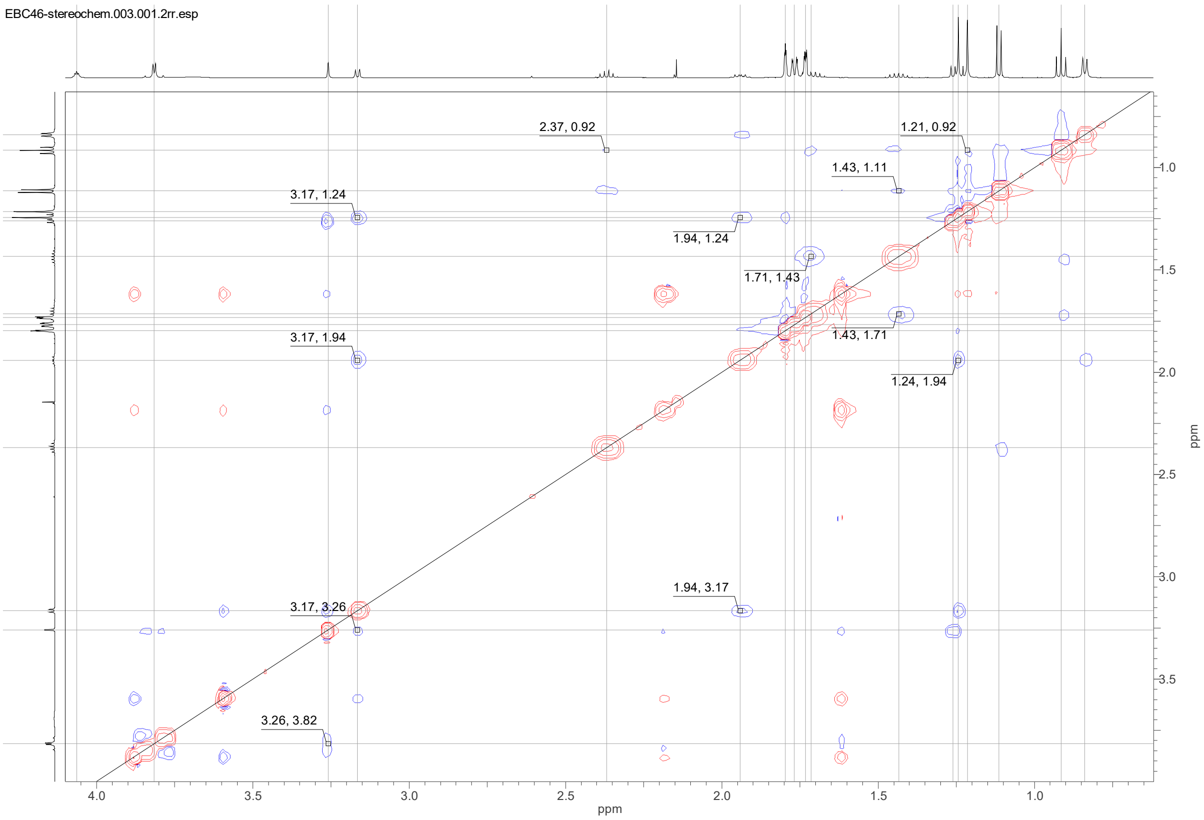

S3.13 NOESY correlation diagrams for EBC-46 from spectrum recorded in $CDCl_3$

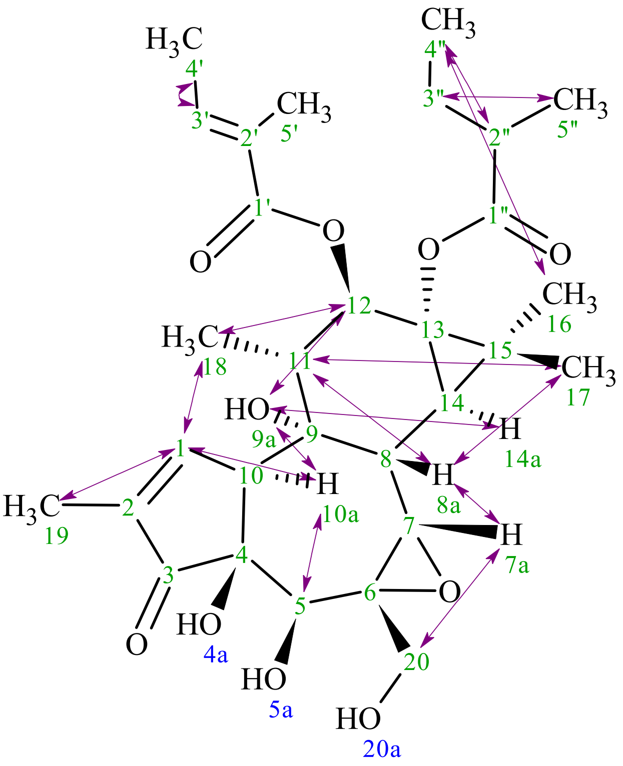

S3.14 IR spectrum for EBC-46.

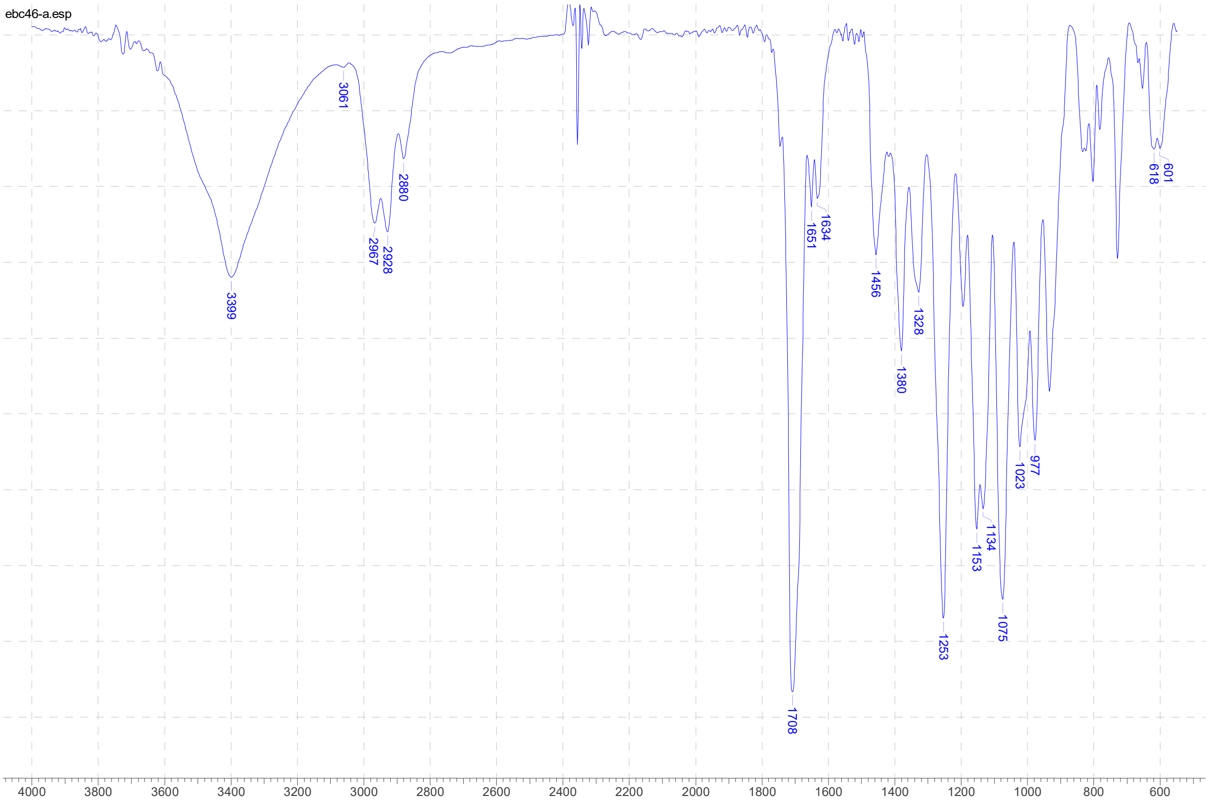

S3.15 ^{1}H NMR spectrum and 0.9-2.5 ppm expansion for EBC-47 recorded in $CDCl_3$.

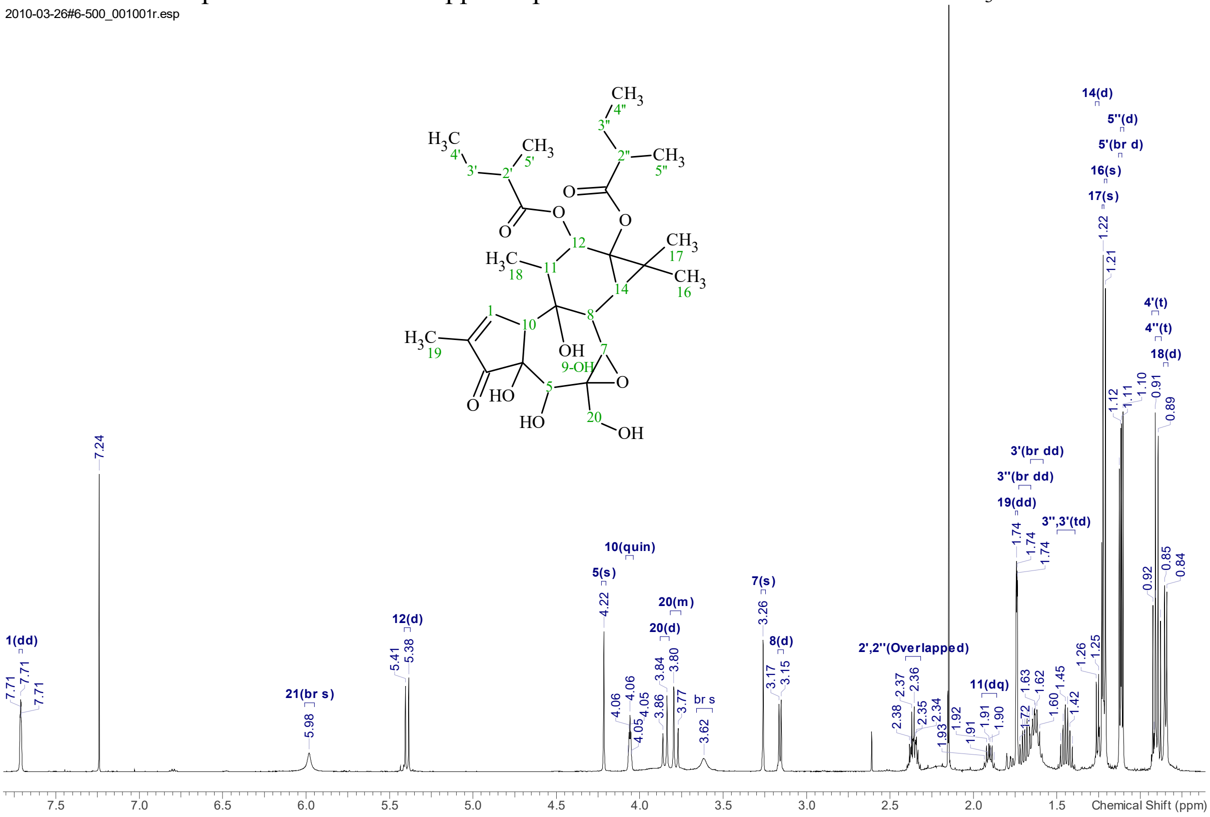

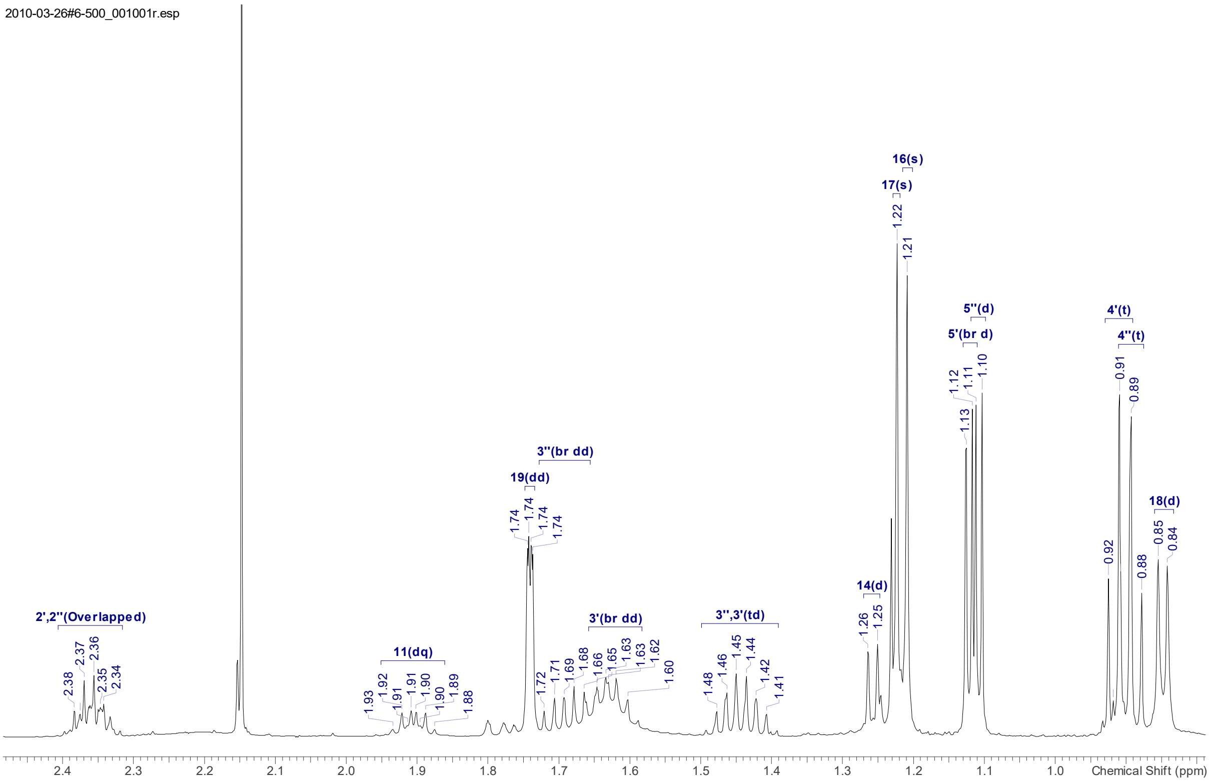

S3.16 ^{13}C NMR spectrum and 10-80 ppm expansion for EBC-47 recorded in $CDCl_3$.

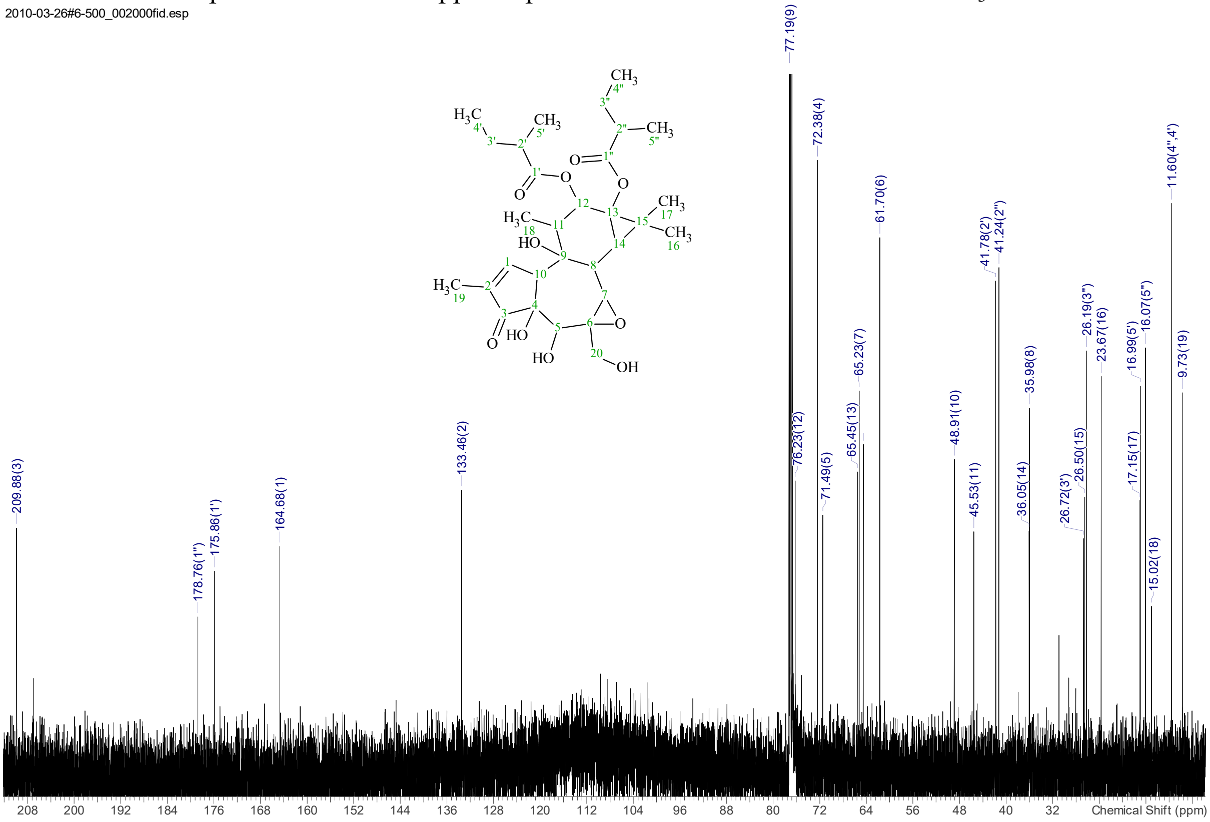

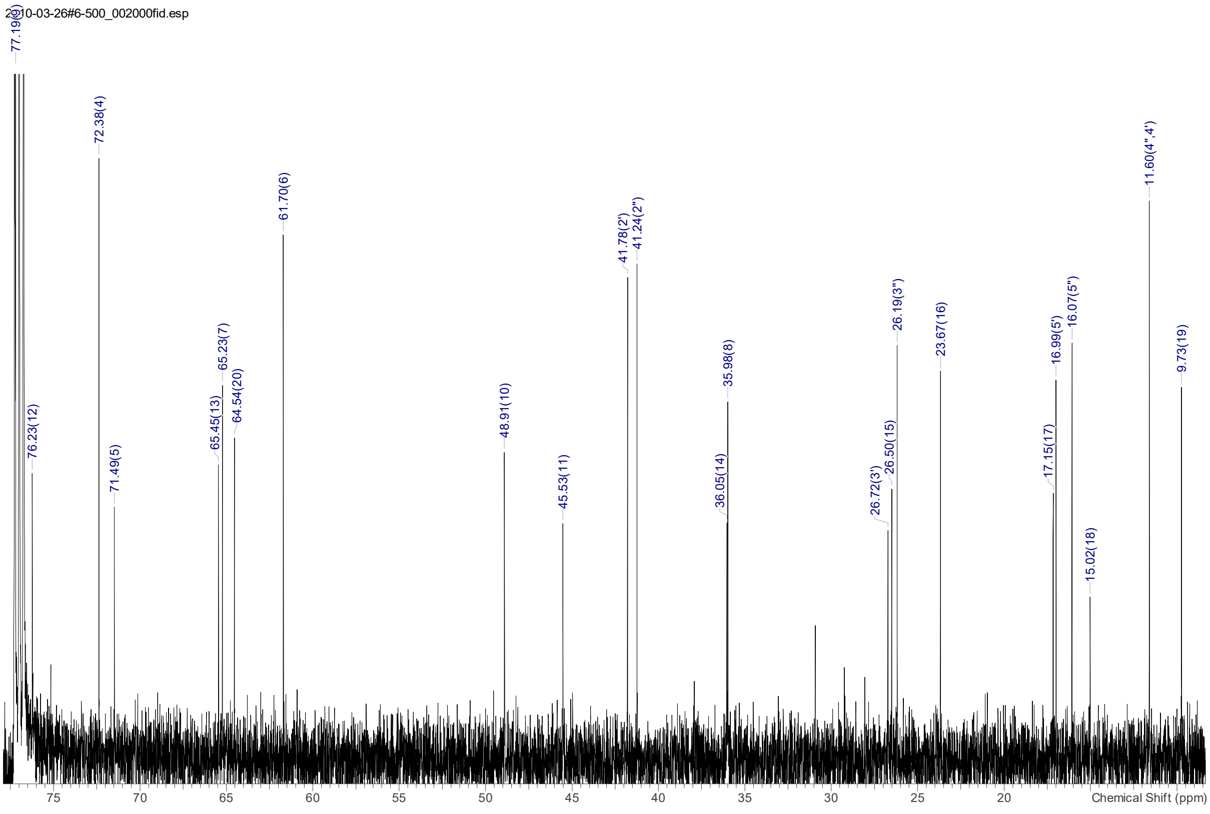

S3.17 1H NMR spectrum and 0.7-2.5 ppm expansion for EBC-59 recorded in $CDCl_3$.

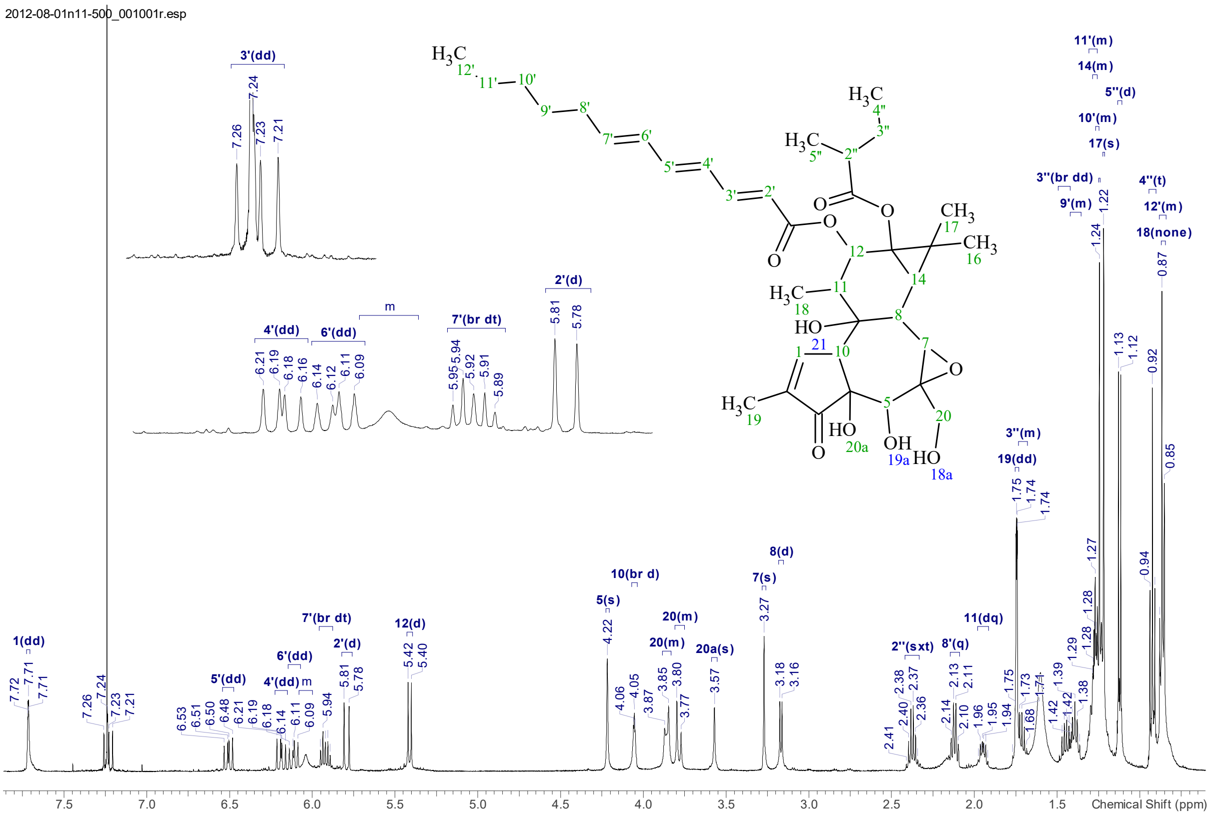

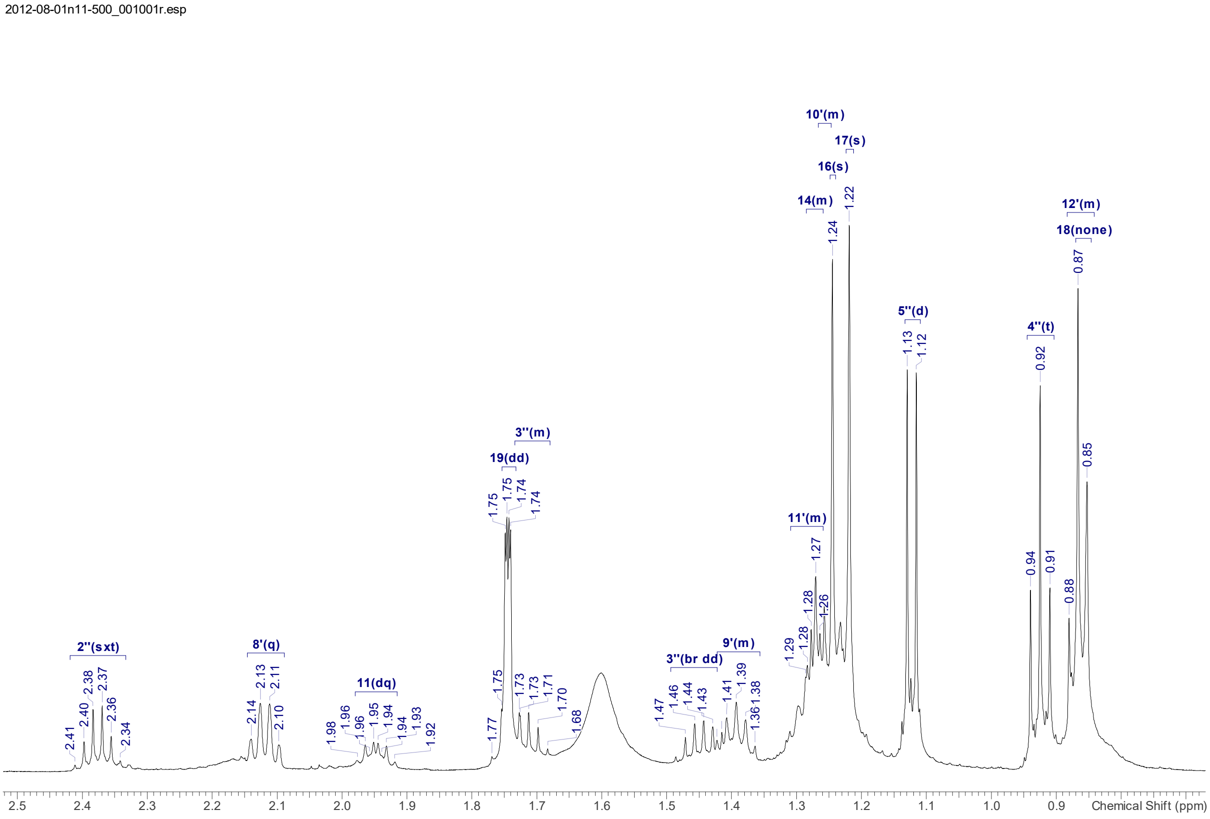

S3.18 ^{13}C NMR spectrum and 5-80 ppm expansion for EBC-59 recorded in $CDCl_3$.

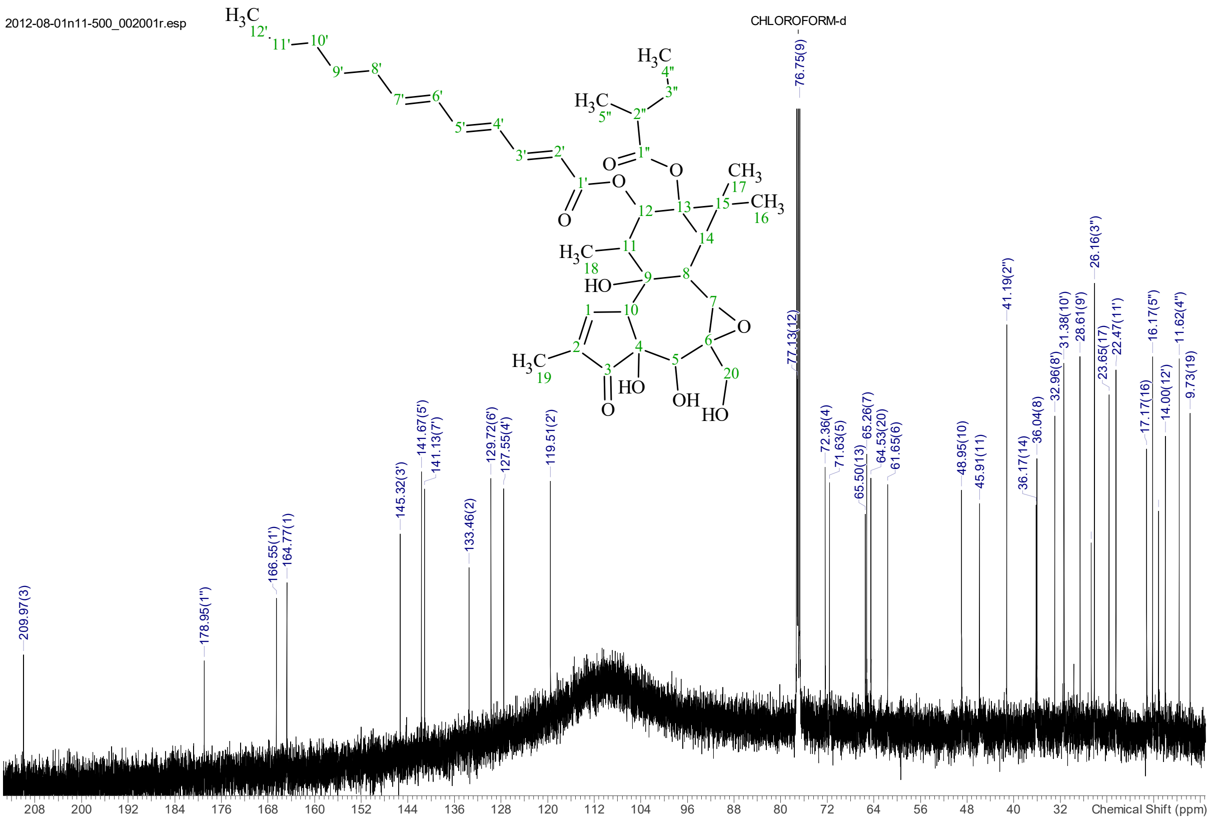

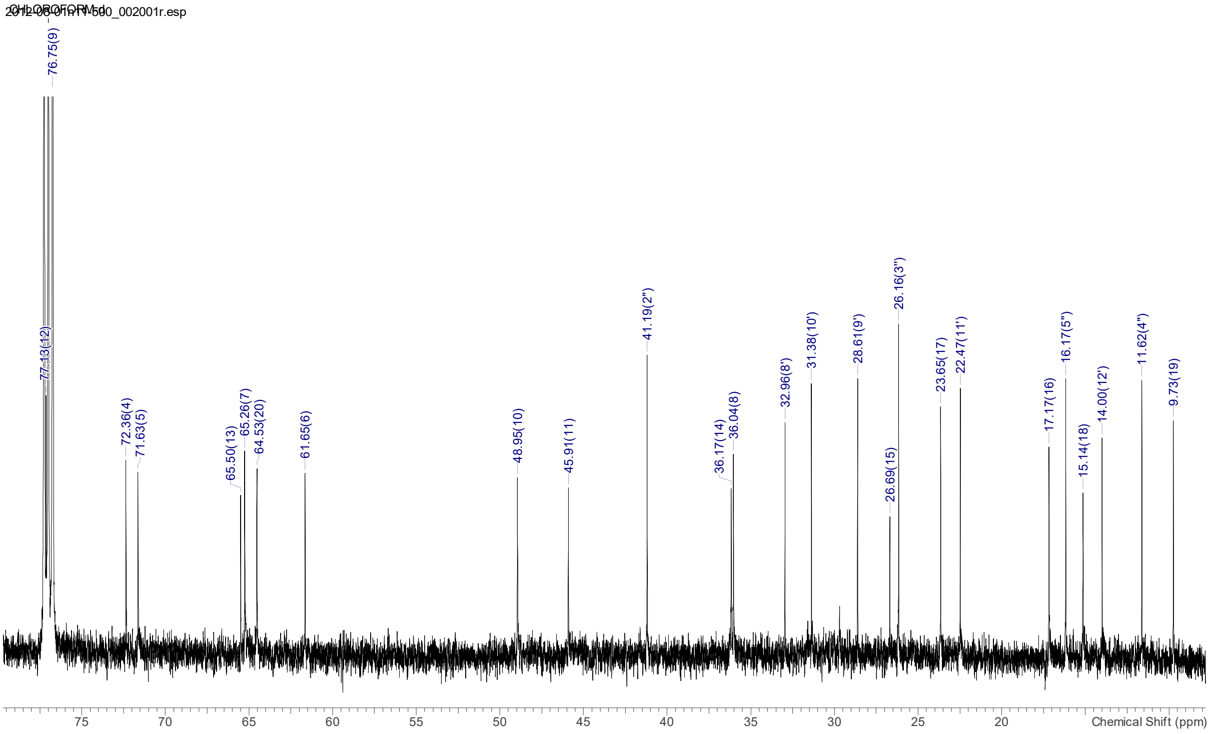

S3.19 NOE correlations diagram for EBC-59. On the diagram selected correlations are drawn.

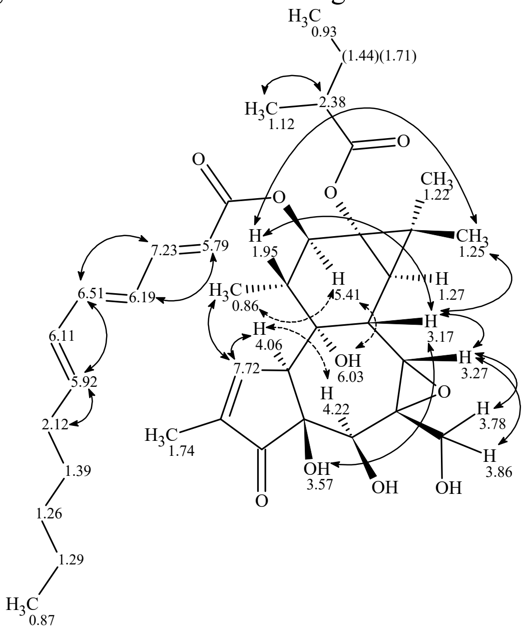

S3.20 1D NOE difference spectra for EBC-59 recorded in $CDCl_3$.

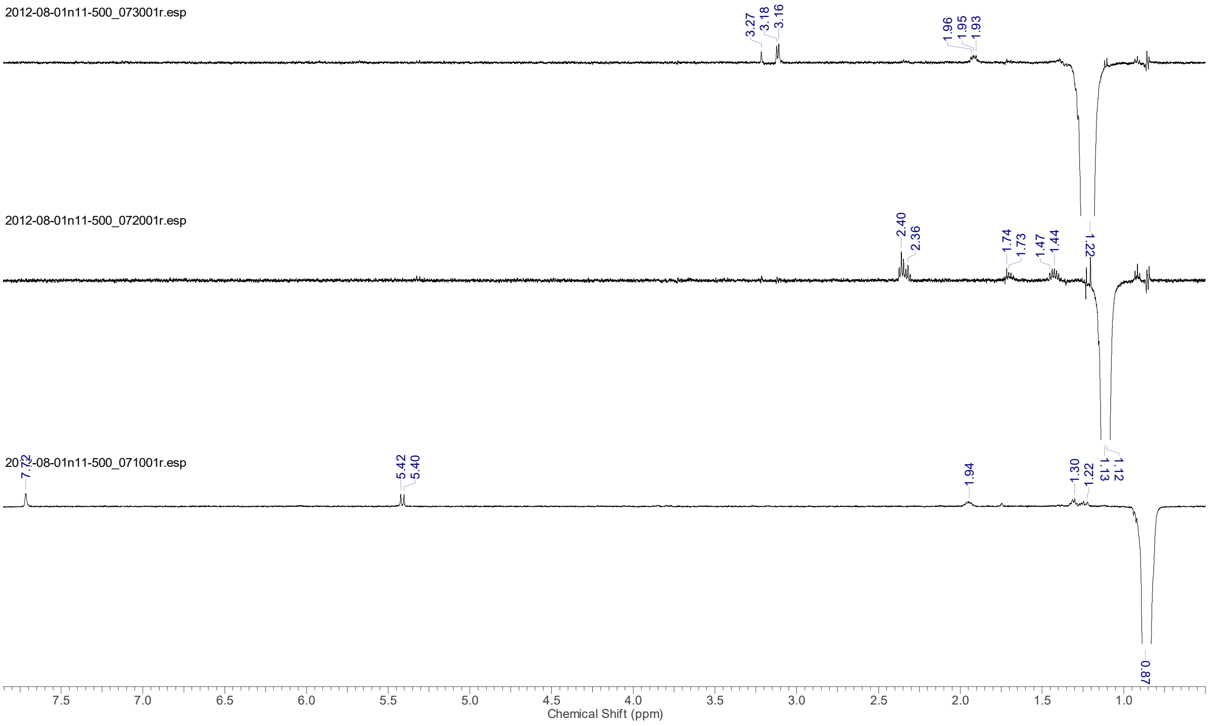

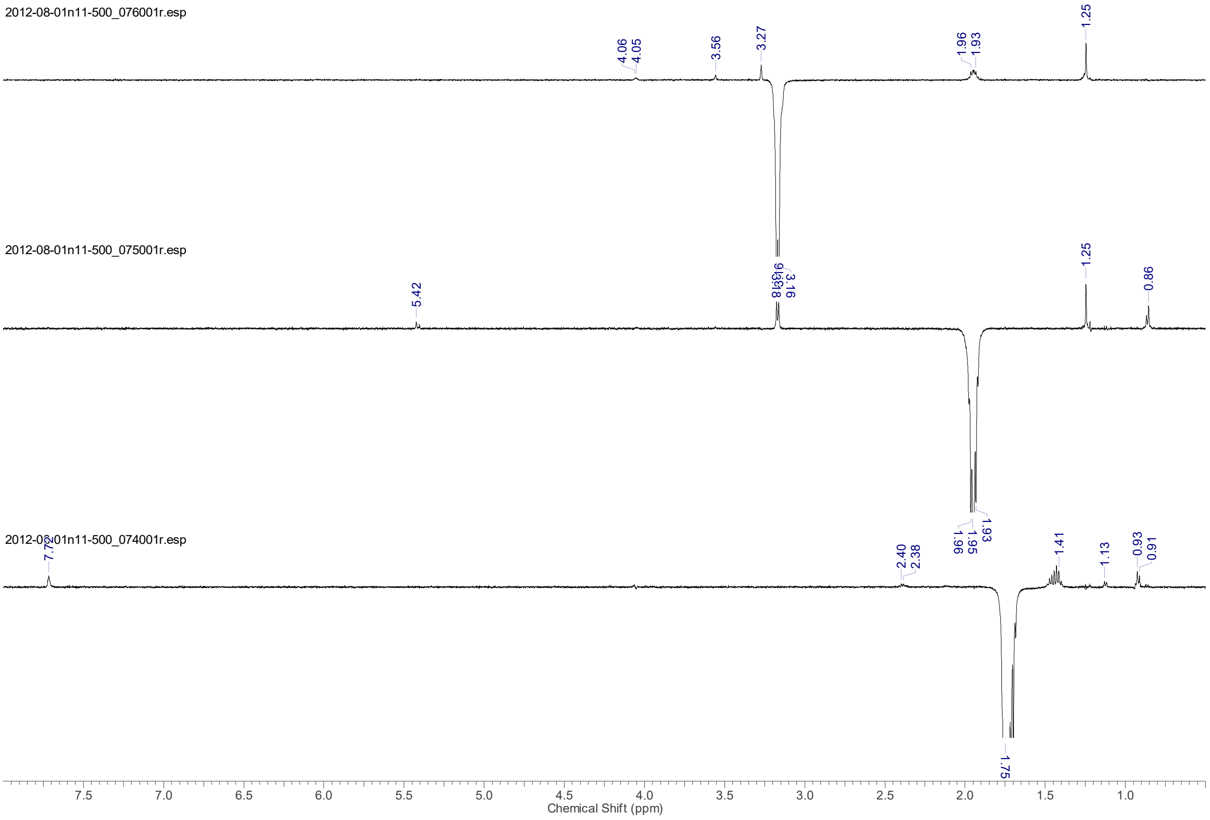
2012-08-01n11-500_076001r.esp
4.06
4.05
3.56
3.27
1.96
1.93
1.25
2012-08-01n11-500_075001r.esp
5.42
3.16
1.25
0.86
1.96
1.95
1.93
2012-08-01n11-500_074001r.esp
7.72
2.40
2.38
1.41
1.13
0.93
0.91
1.75
7.5 7.0 6.5 6.0 5.5 5.0 4.5 4.0 3.5 3.0 2.5 2.0 1.5 1.0
Chemical Shift (ppm)

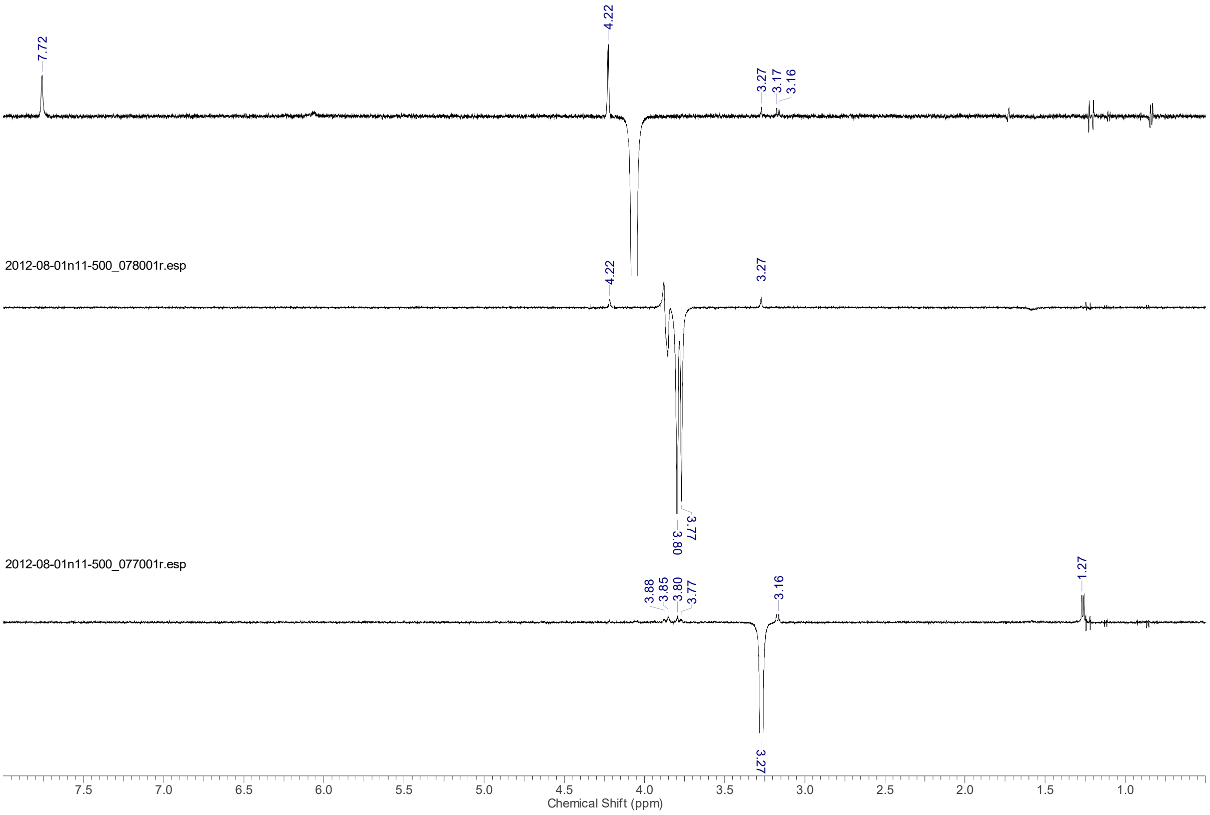
7.72
4.22
3.27
3.17
3.16
2012-08-01n11-500_078001r.esp
4.22
3.27
3.80
3.77
2012-08-01n11-500_077001r.esp
3.88
3.85
3.80
3.77
3.16
1.27
3.27
7.5 7.0 6.5 6.0 5.5 5.0 4.5 4.0 3.5 3.0 2.5 2.0 1.5 1.0
Chemical Shift (ppm)

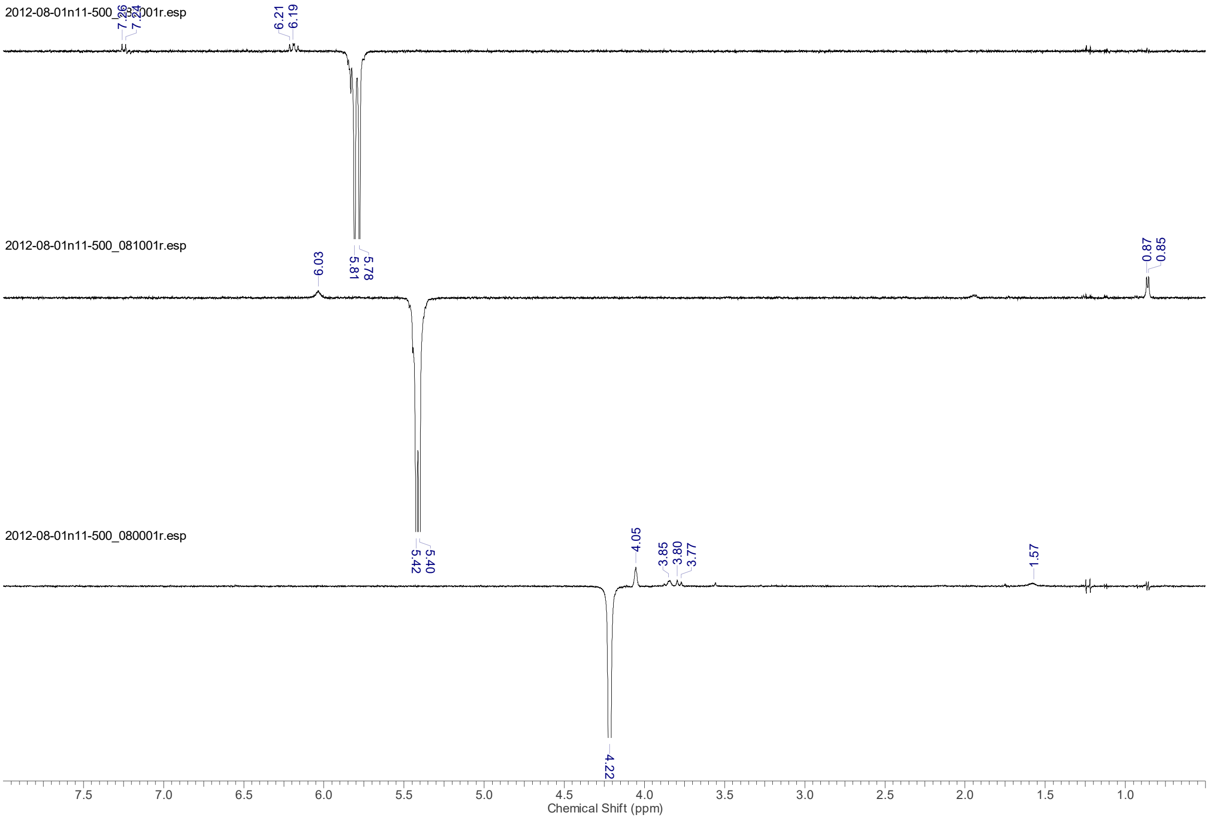
2012-08-01n11-500_081001r.esp
2012-08-01n11-500_080001r.esp
Chemical Shift (ppm)

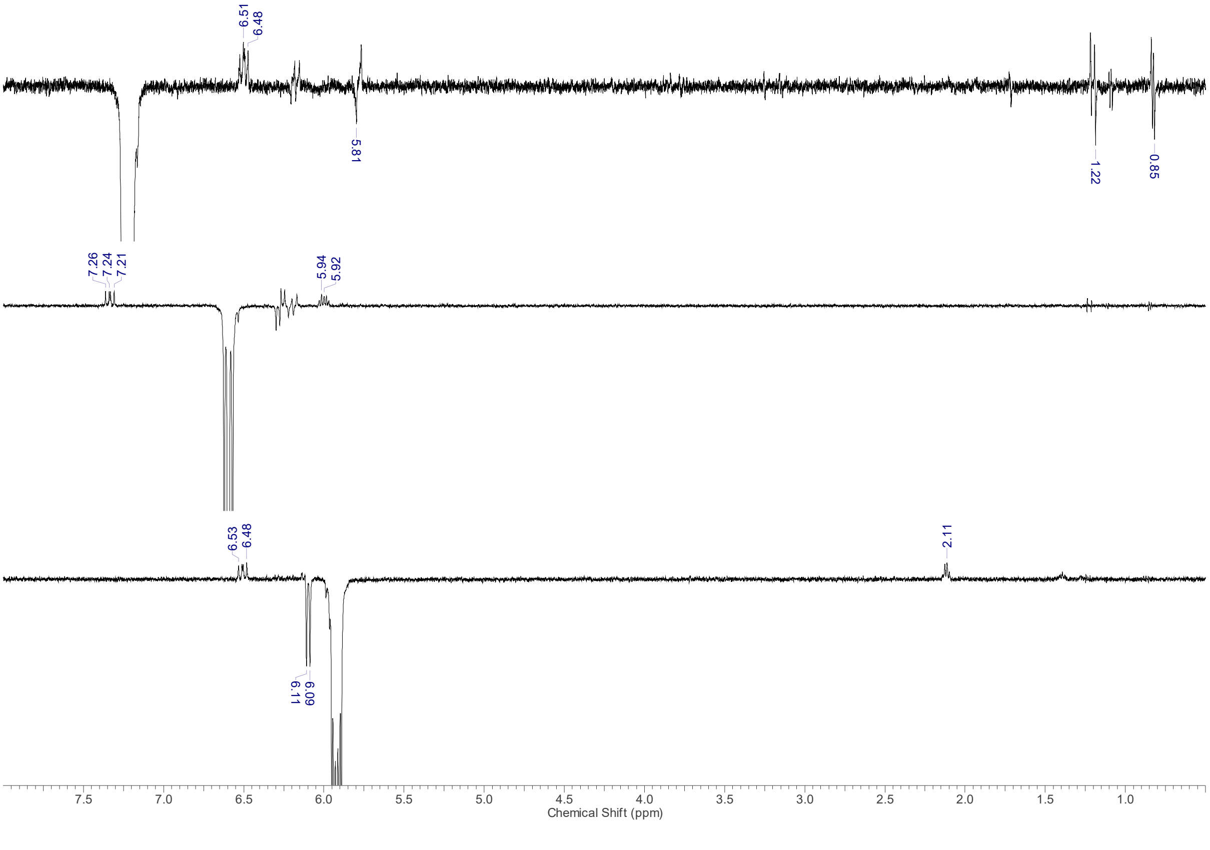
Chemical Shift (ppm)

S3.21 ^{1}H NMR spectrum for EBC-83 recorded in $CDCl_3$.

eb610_eb_32-19_001001r.esp

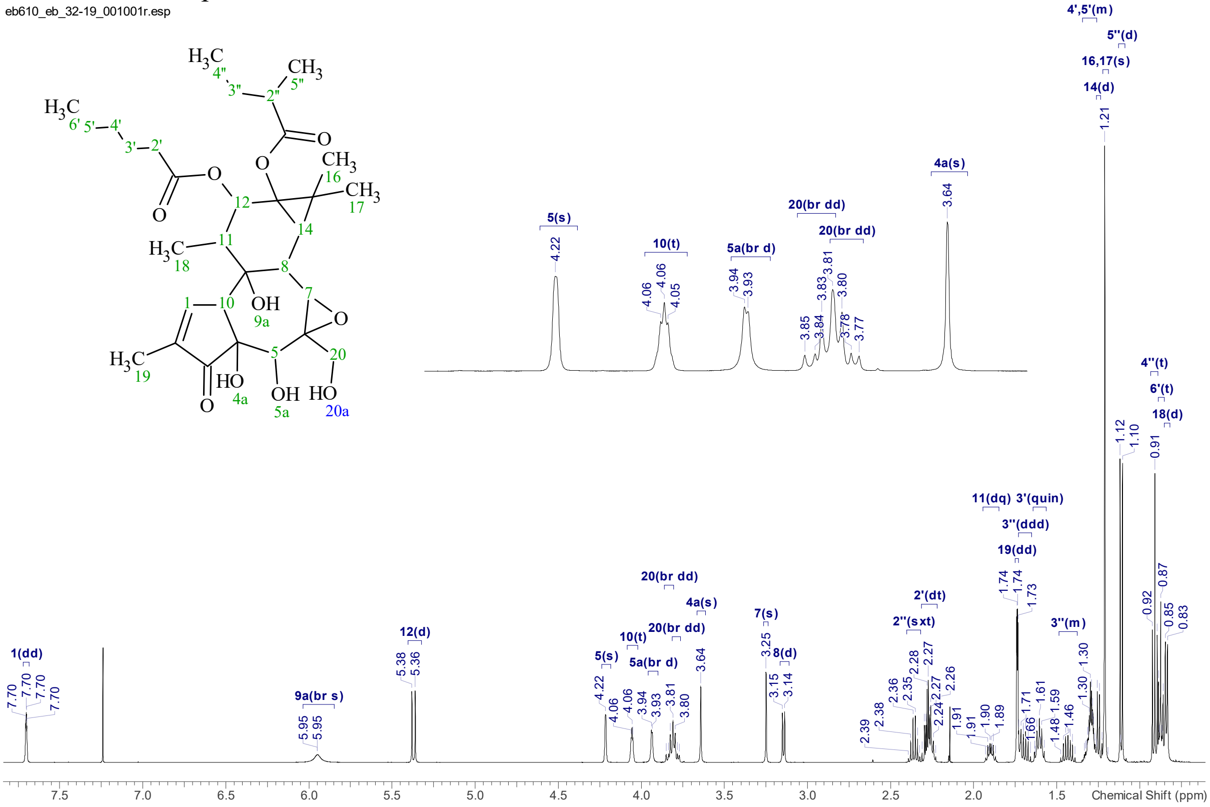

eb610_eb_32-19_001001r.esp

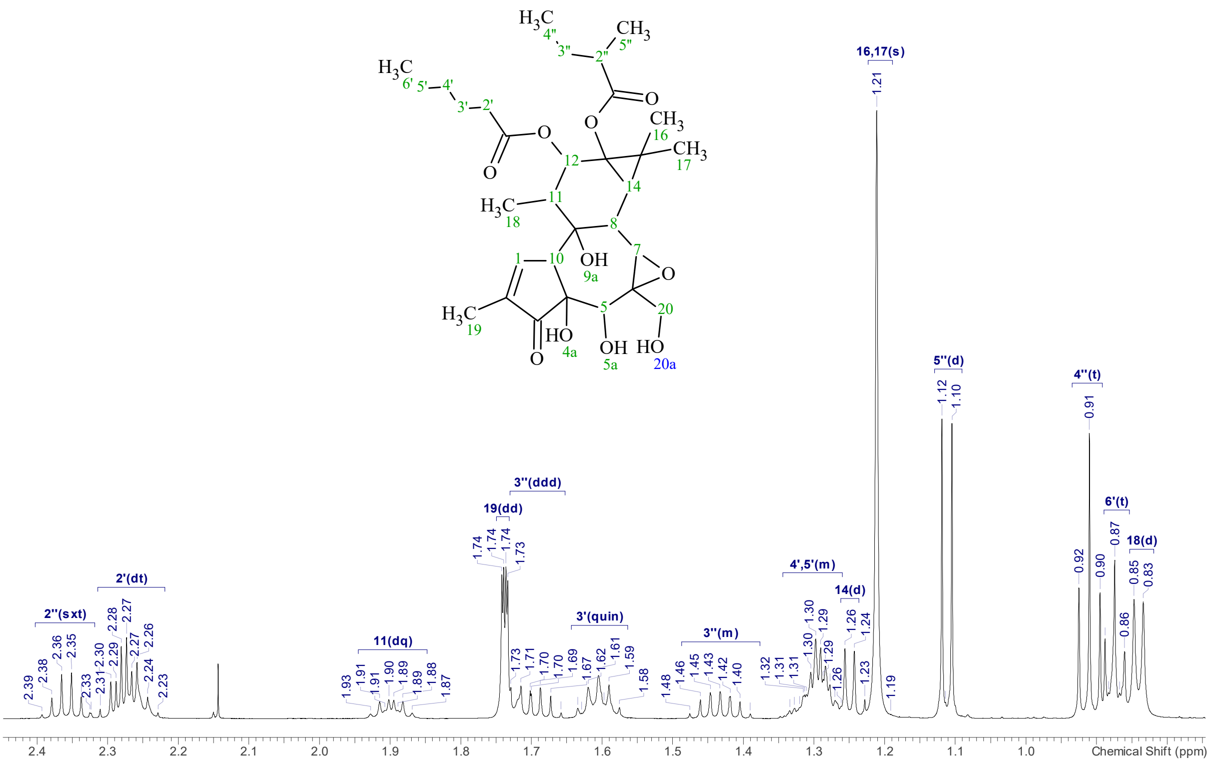

S3.22 ^{13}C NMR spectrum for EBC-83 recorded in $CDCl_3$.

eb610_eb_32-19_002001r.esp

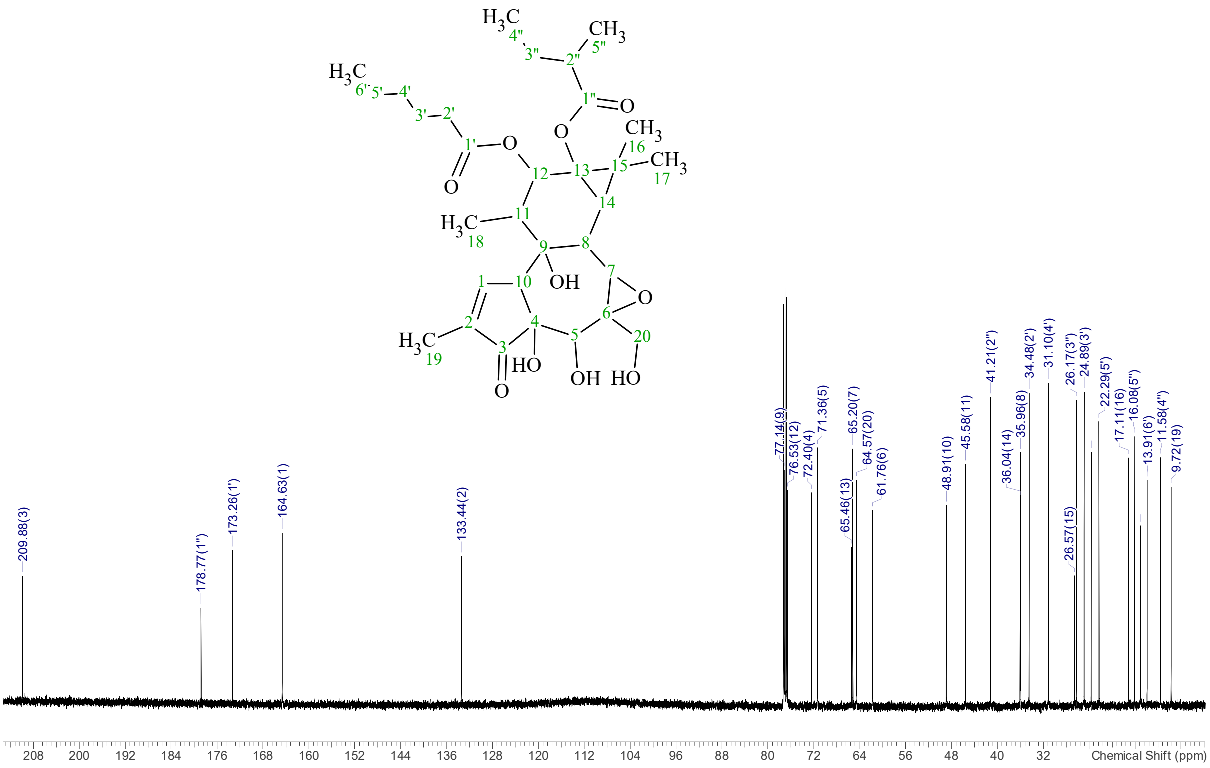

eb610_eb_32-19_002001r.esp

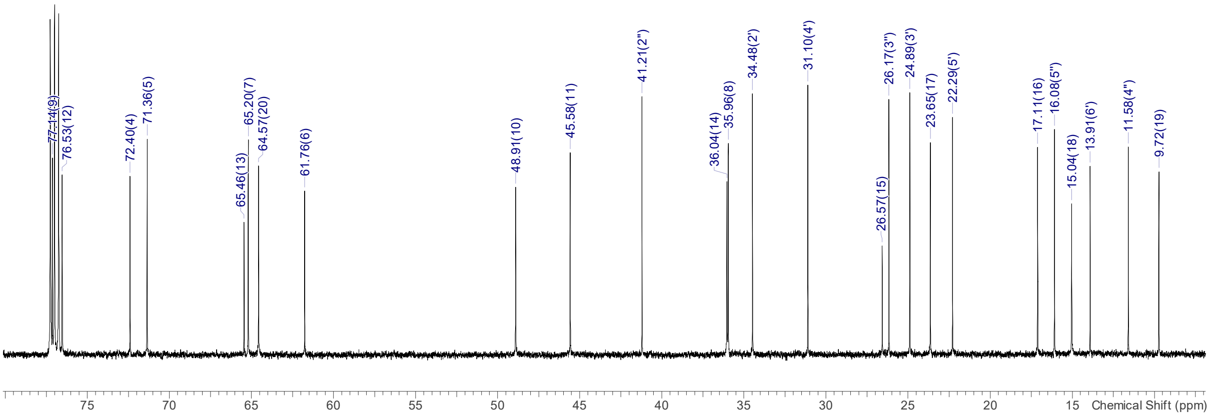

S3.23 ^{1}H NMR spectrum and expansion for EBC-146 recorded in $CDCl_3$.

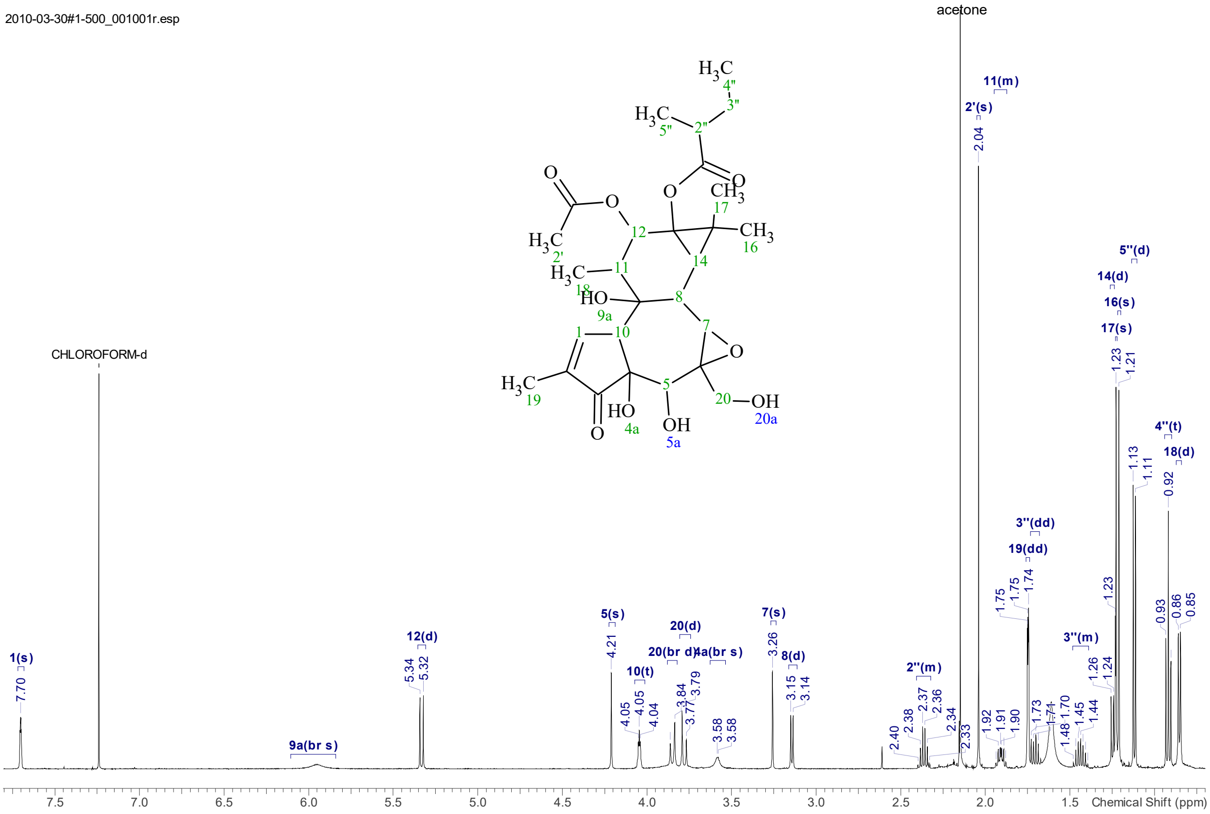

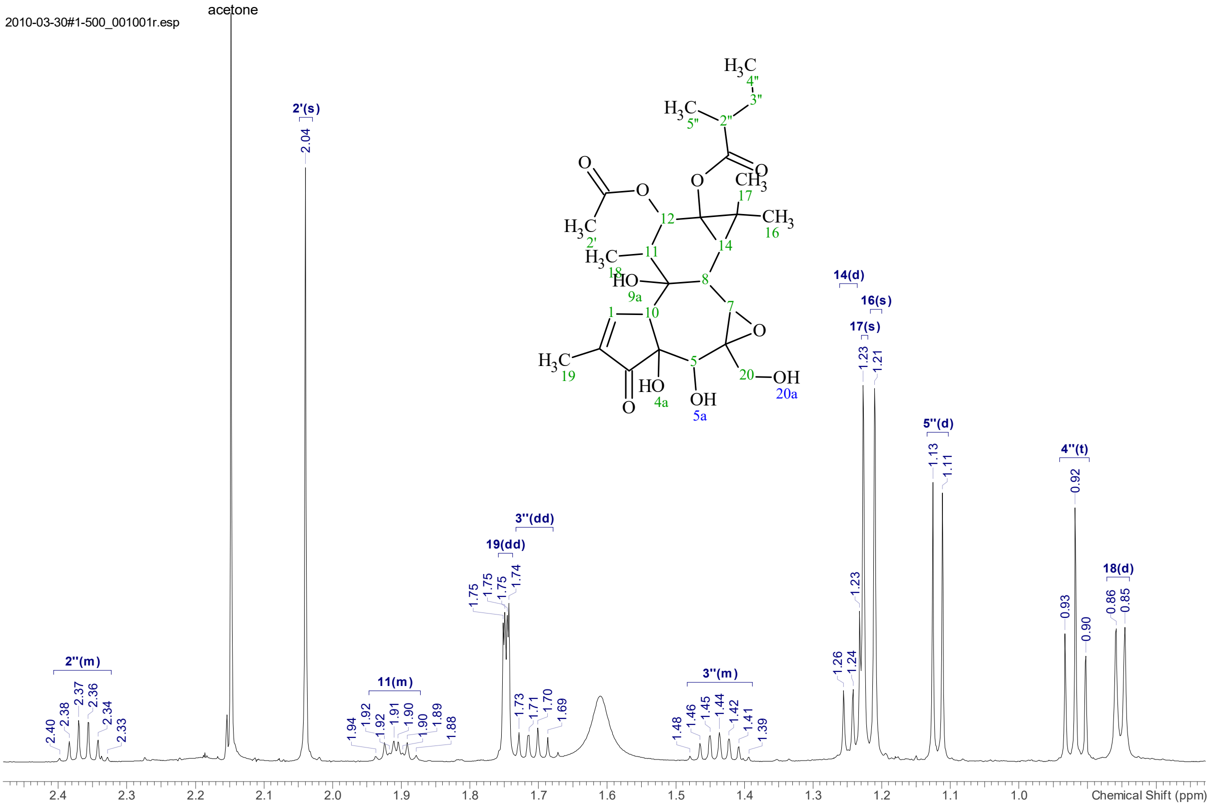

S3.24 ^{13}C NMR spectrum and expansion for EBC-146 recorded in $CDCl_3$.

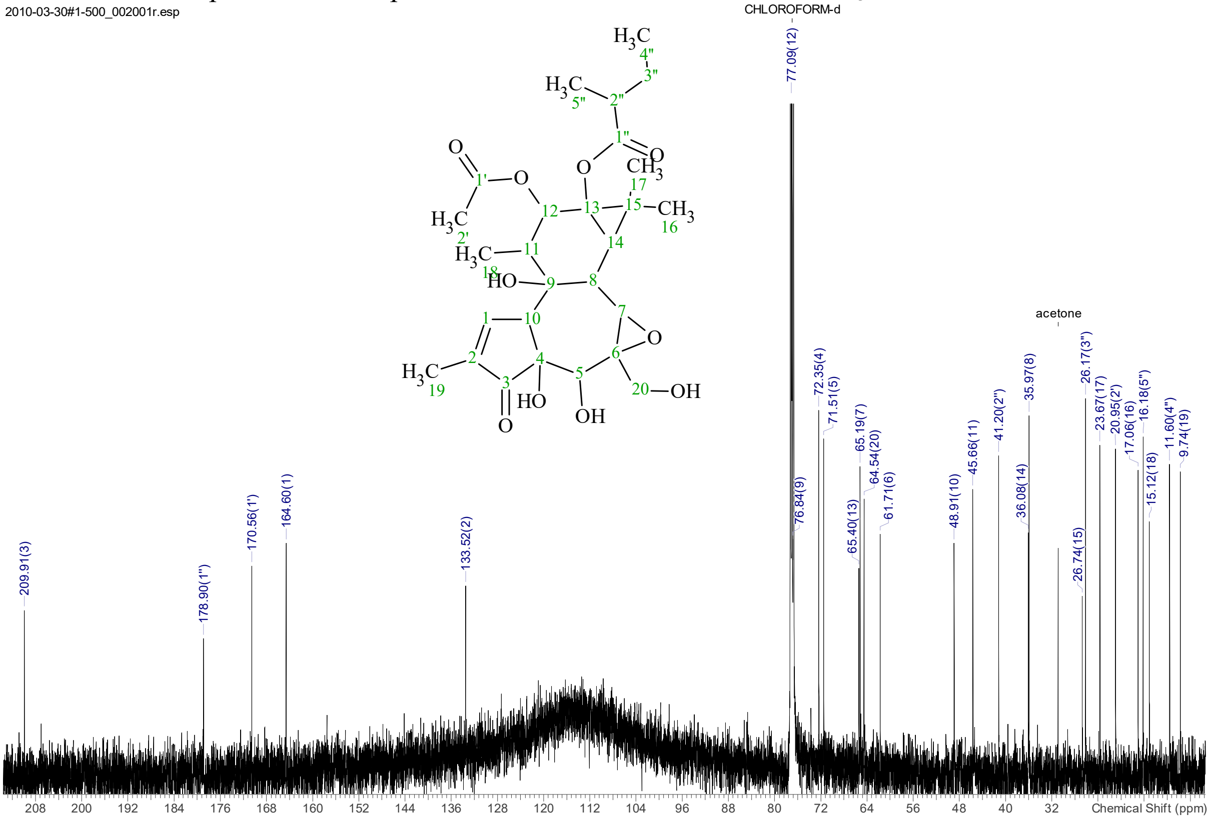

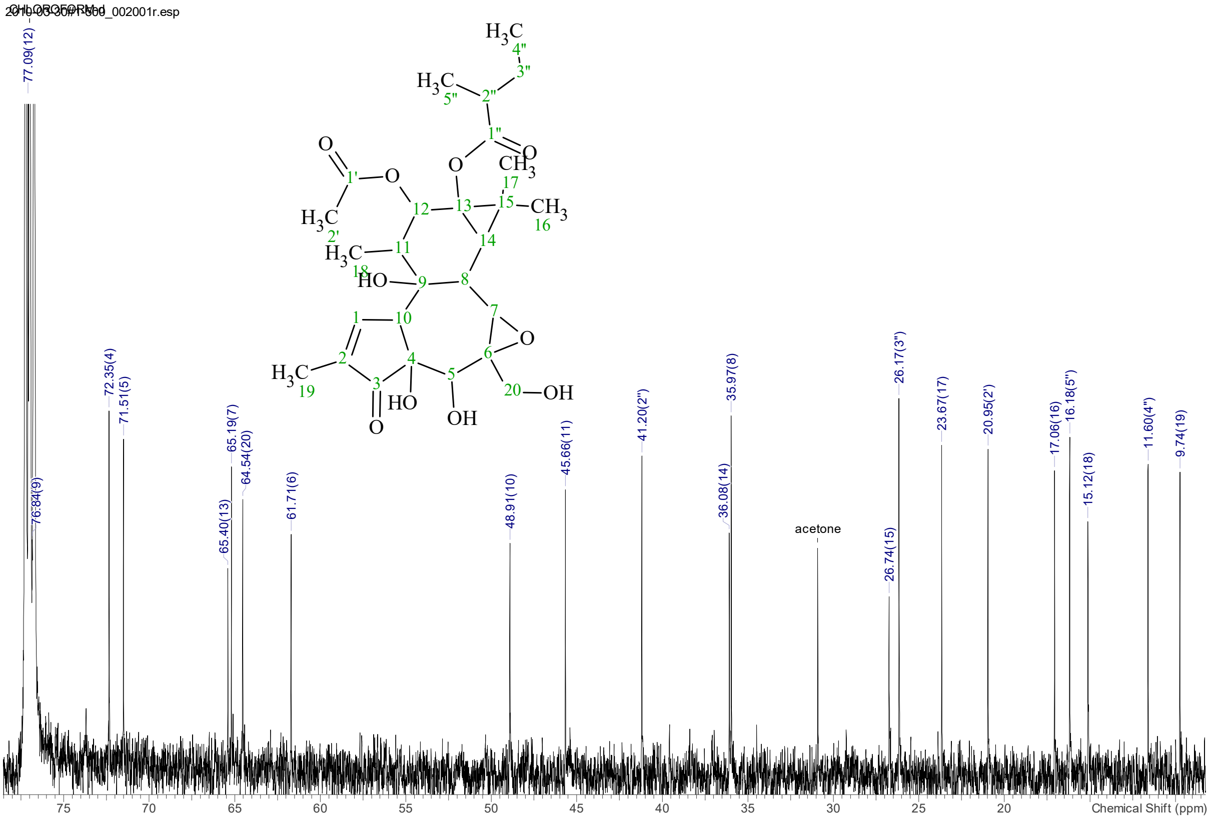

S3.25 ^{1}H NMR spectrum and expansion for EBC-147 recorded in $CDCl_3$.

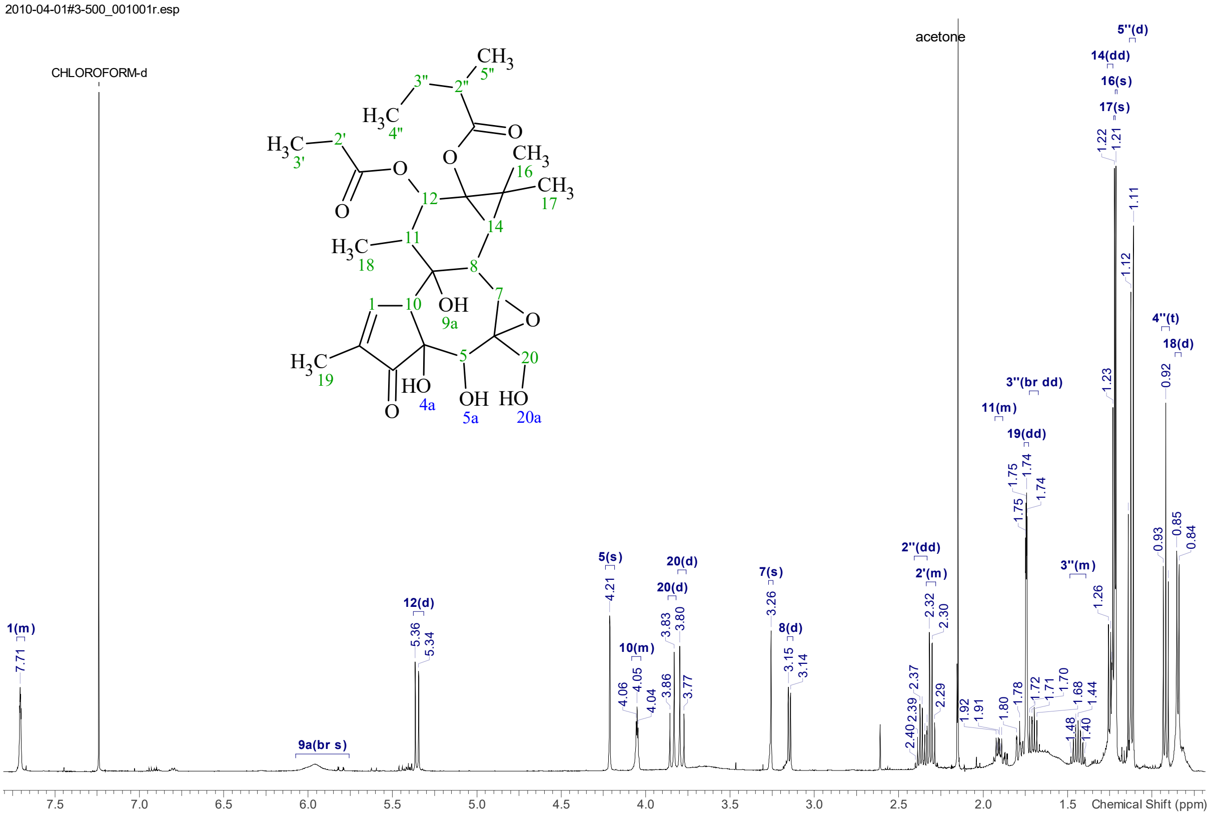

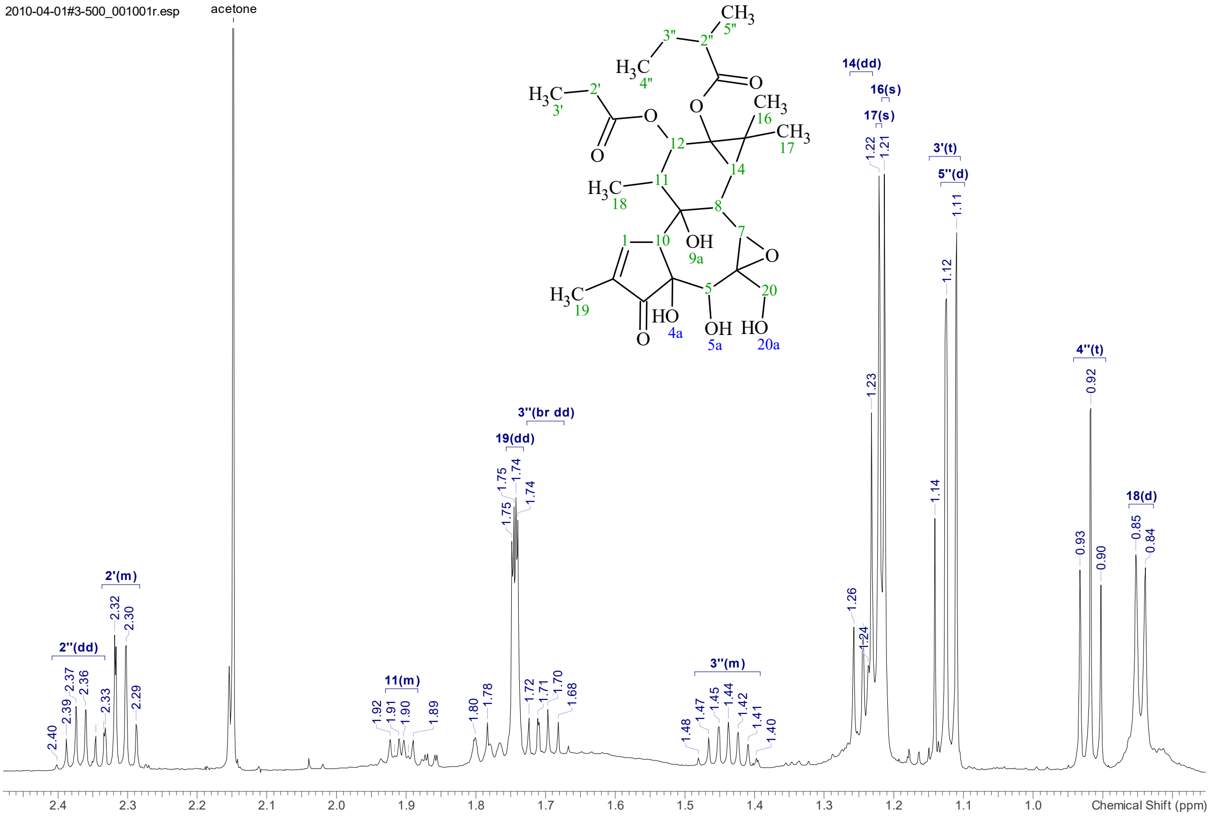

S3.26 ^{13}C NMR spectrum and expansions for EBC-147 recorded in $CDCl_3$.

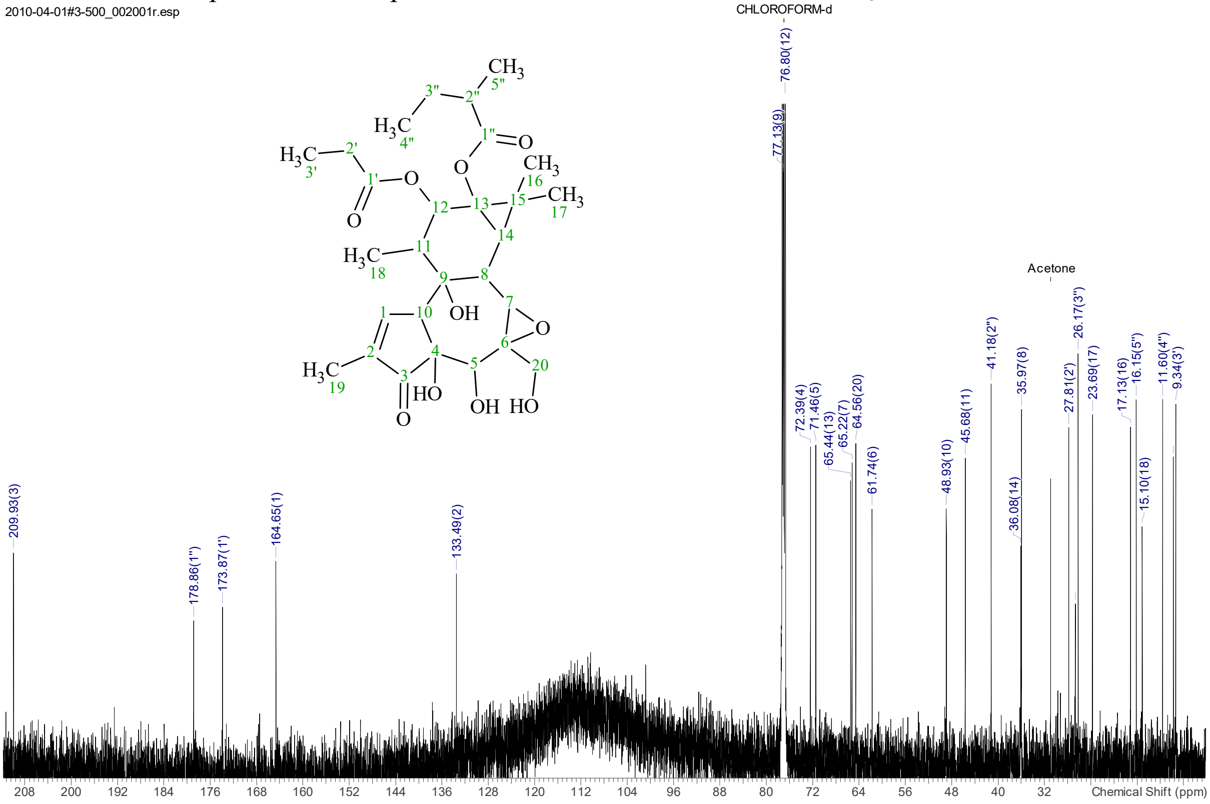

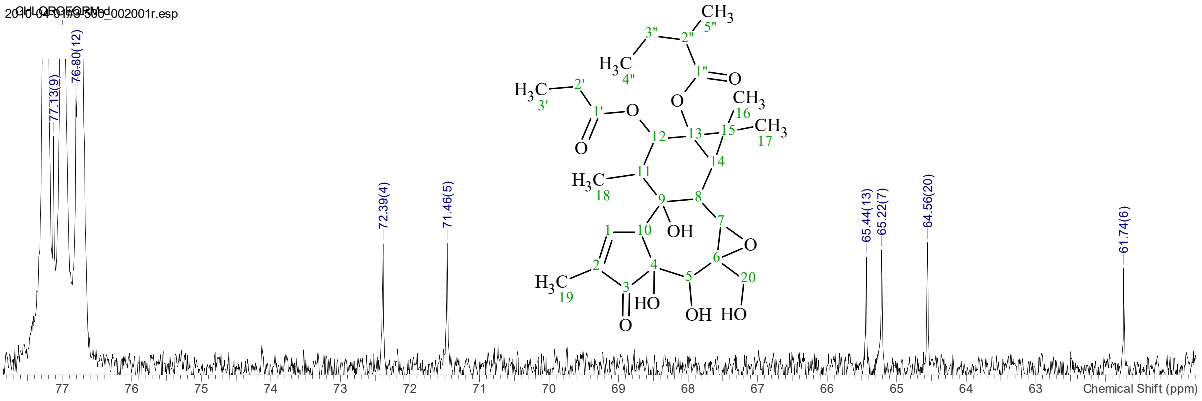

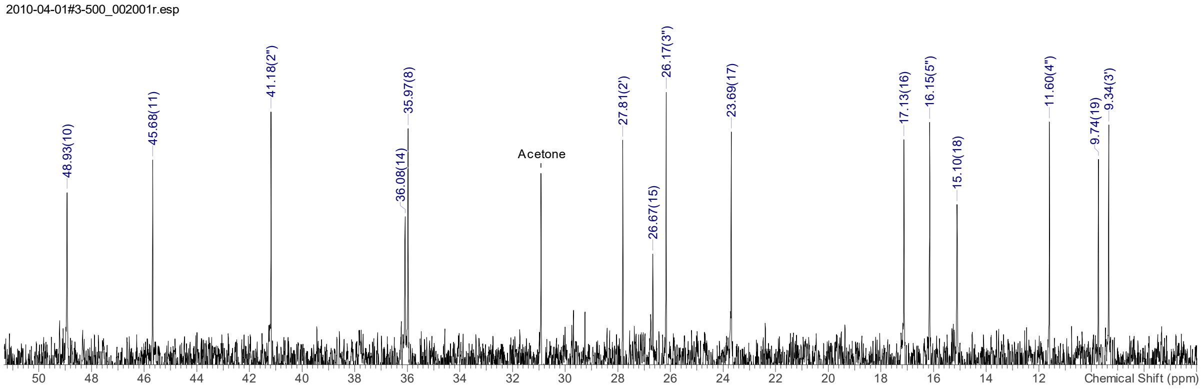

S3.27 ^{1}H NMR spectrum for EBC-148 recorded in $CDCl_3$.

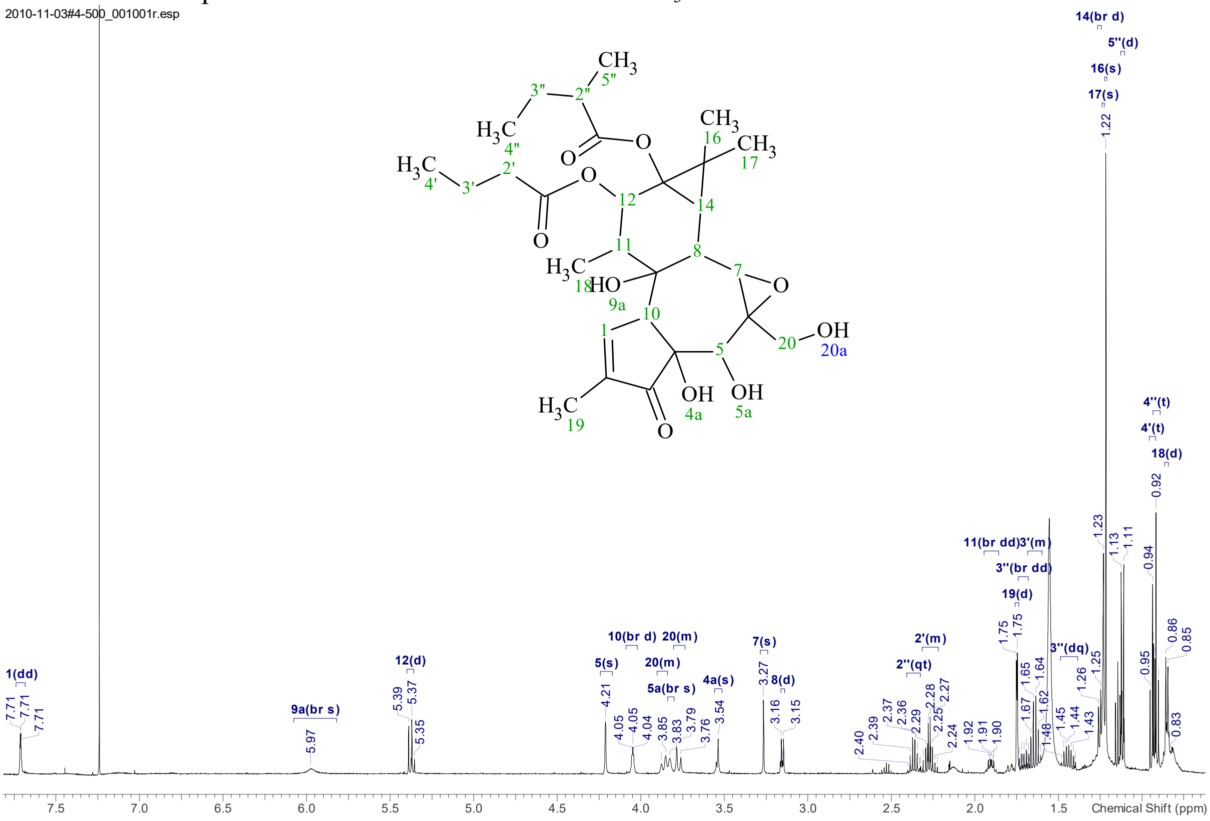

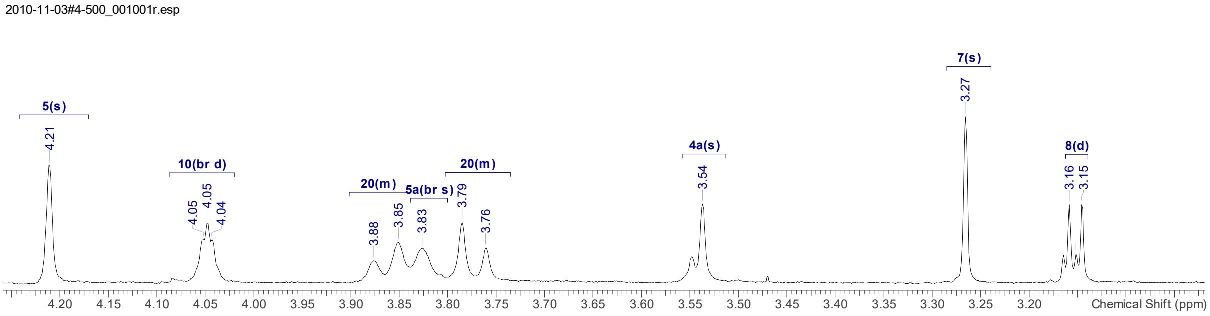

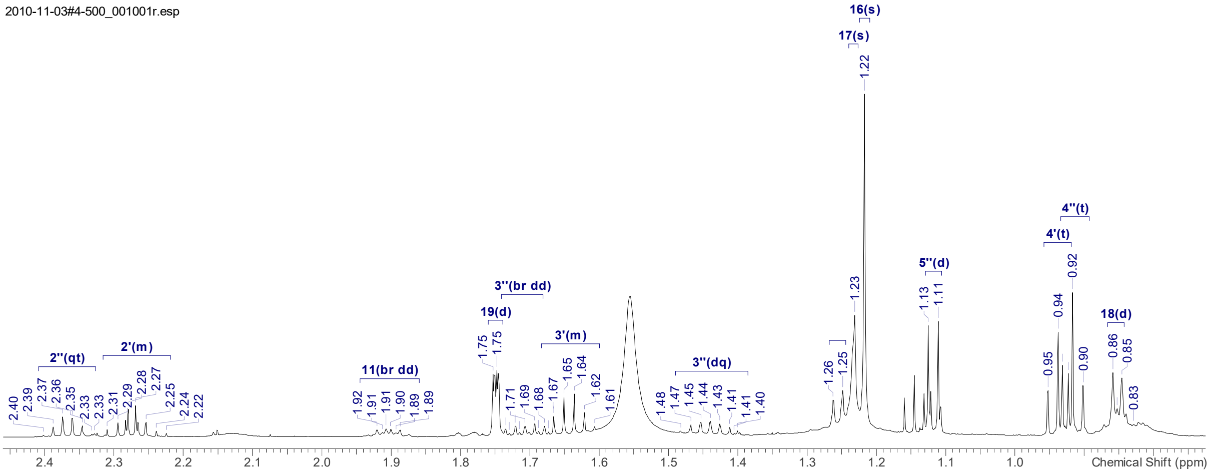

S3.28 ^{13}C NMR spectrum for EBC-148 recorded in $CDCl_3$.

2010-04-25#1-400_002001r.esp

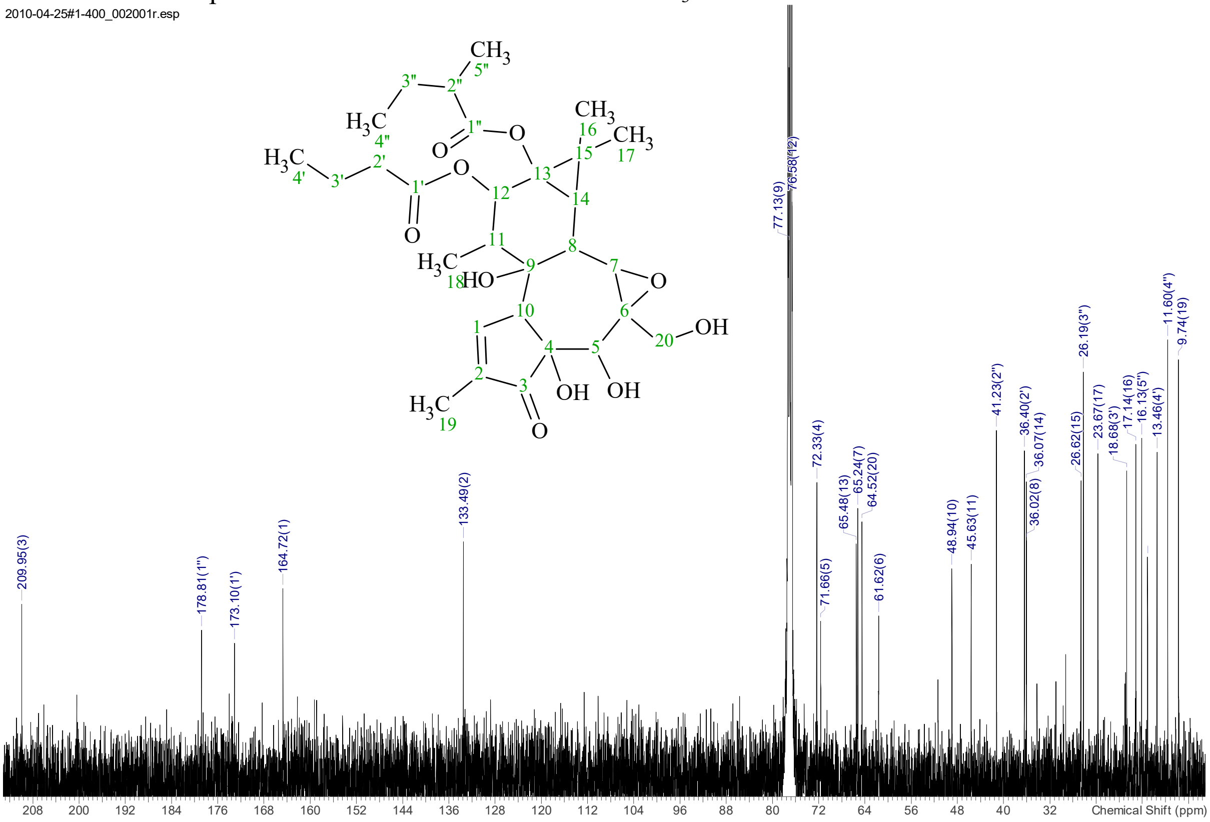

2010-04-25#1-400_002001r.esp

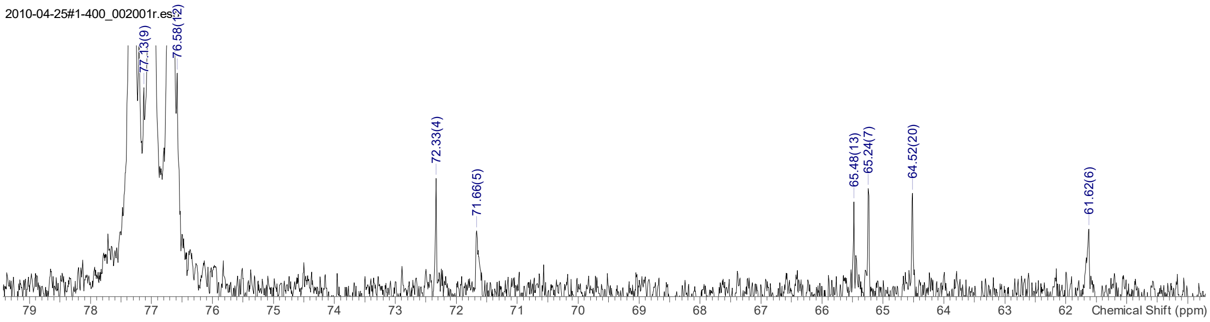

2010-04-25#1-400_002001r.esp

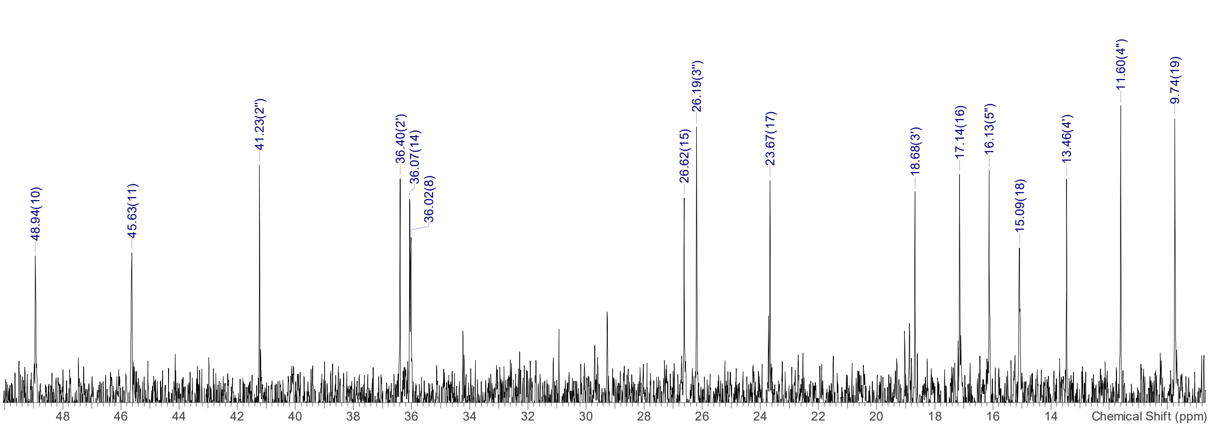

S3.29 ^{1}H NMR spectrum for EBC-158 recorded in $CDCl_3$.

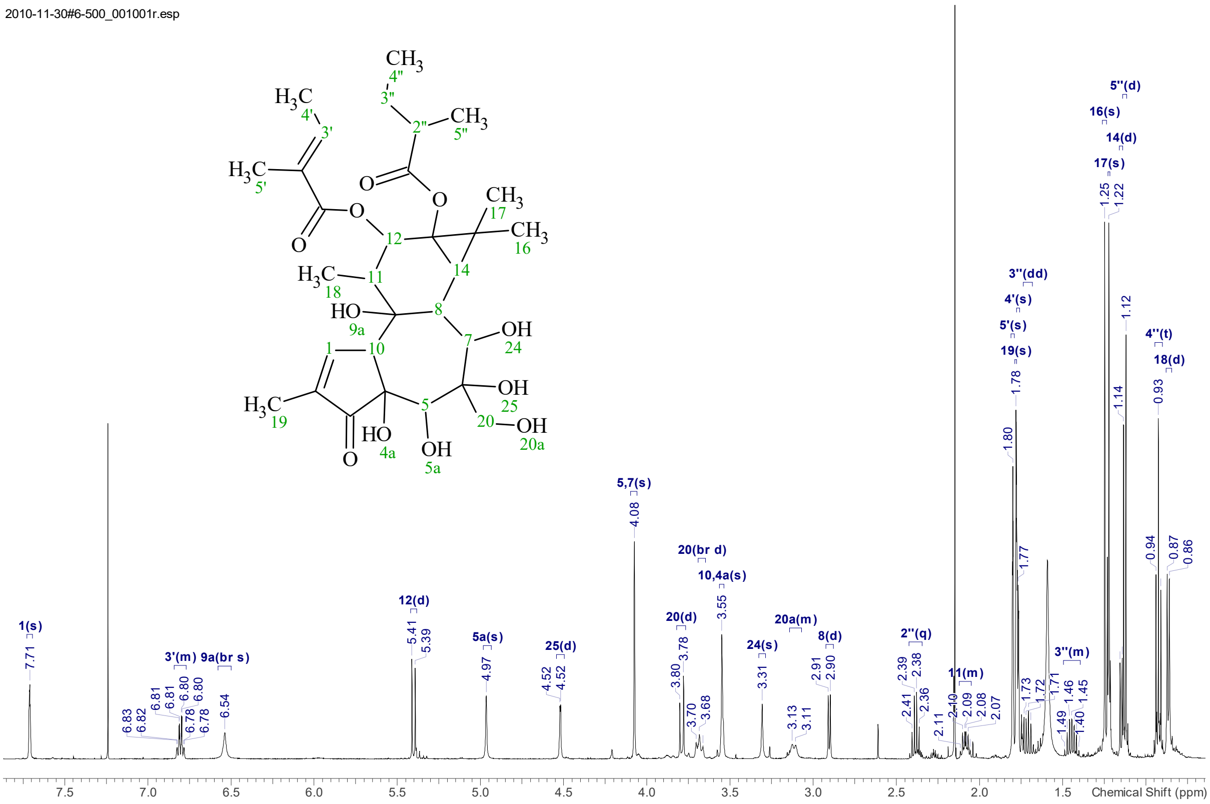

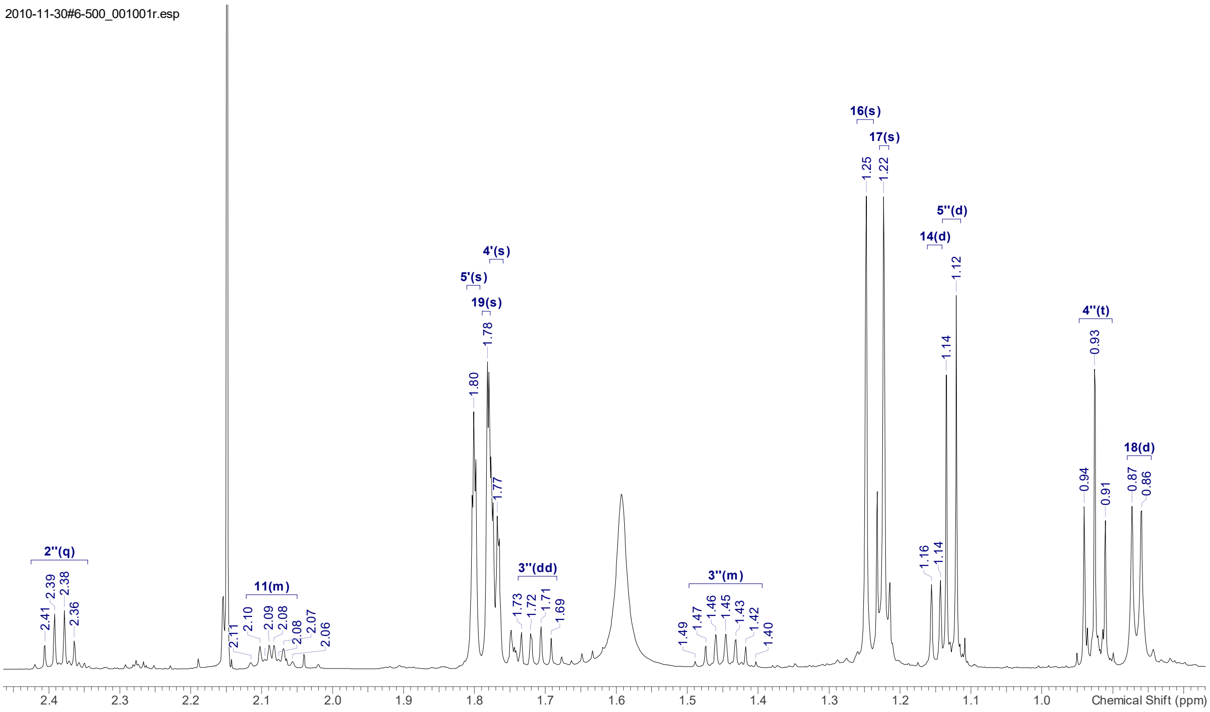

S3.30 ^{13}C NMR spectrum for EBC-158 recorded in $CDCl_3$.

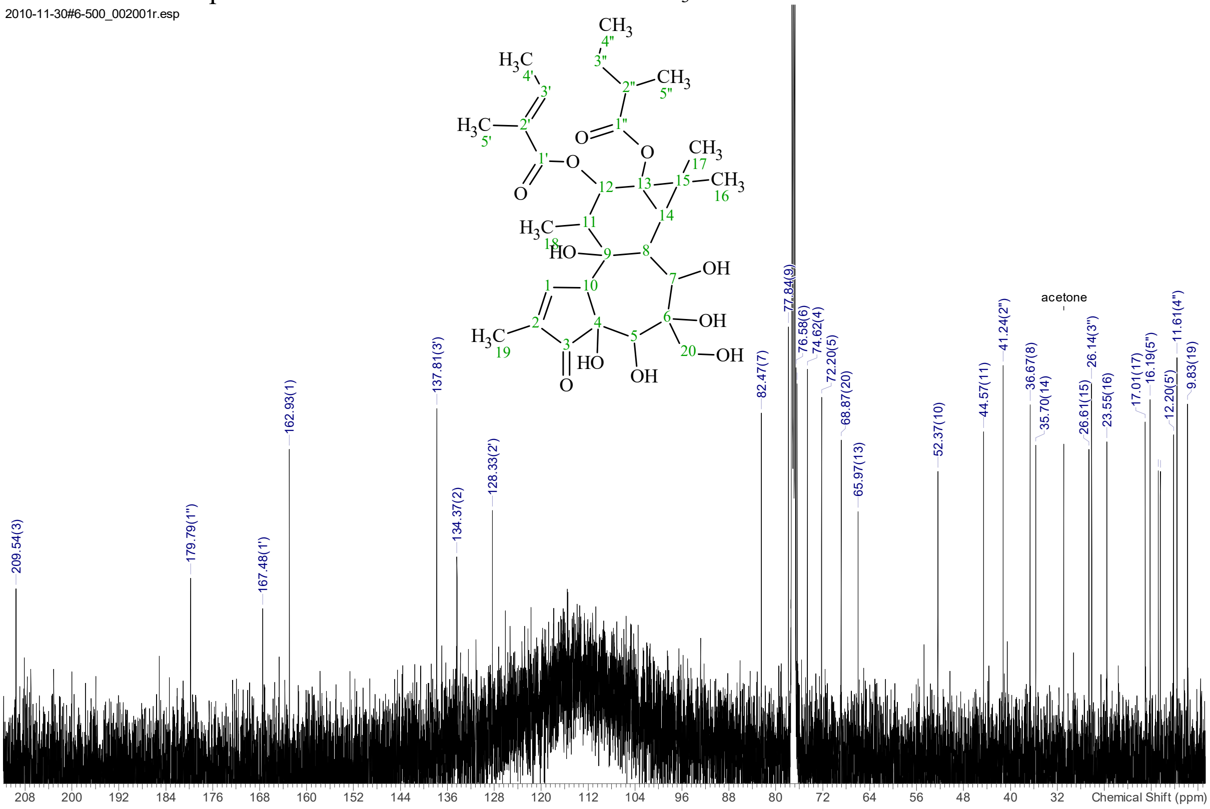

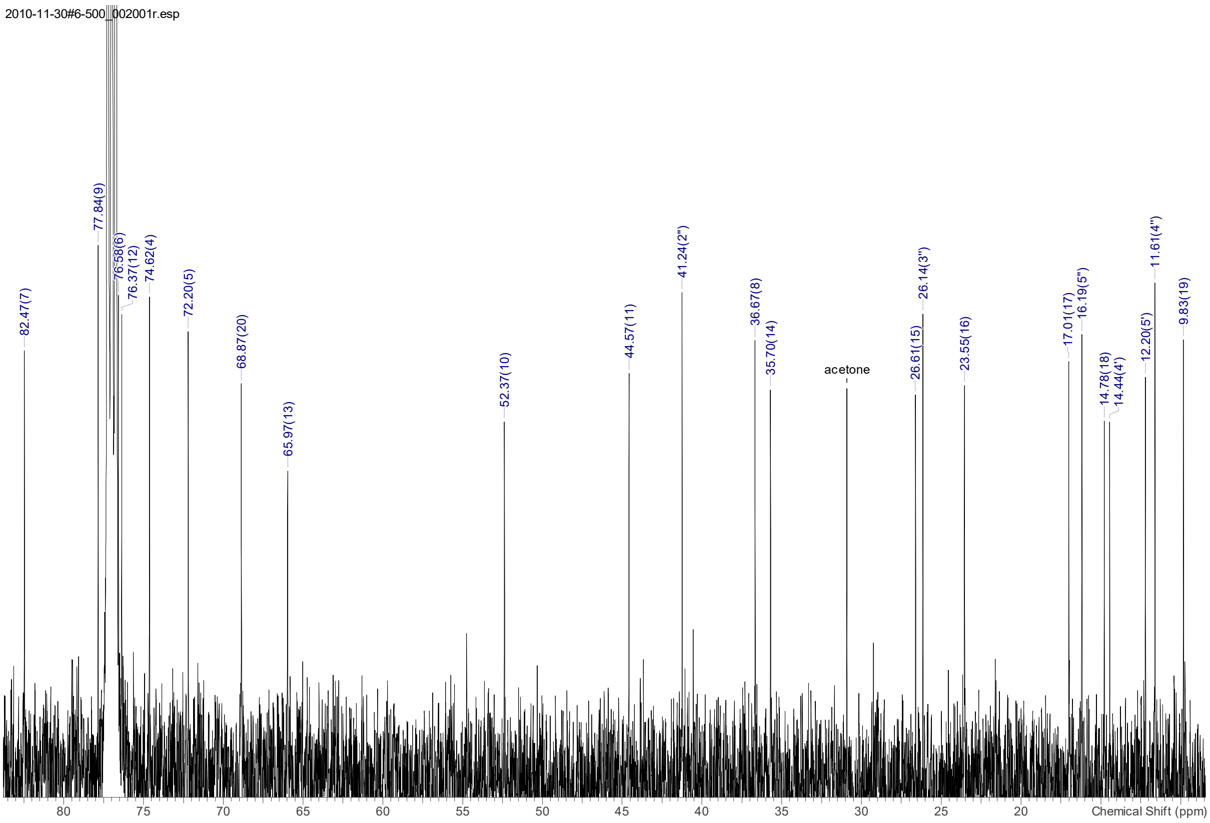

S3.31 ^{1}H NMR spectrum for EBC-161 recorded in $CDCl_3$.

2011-02-04n3-500_001001r.esp

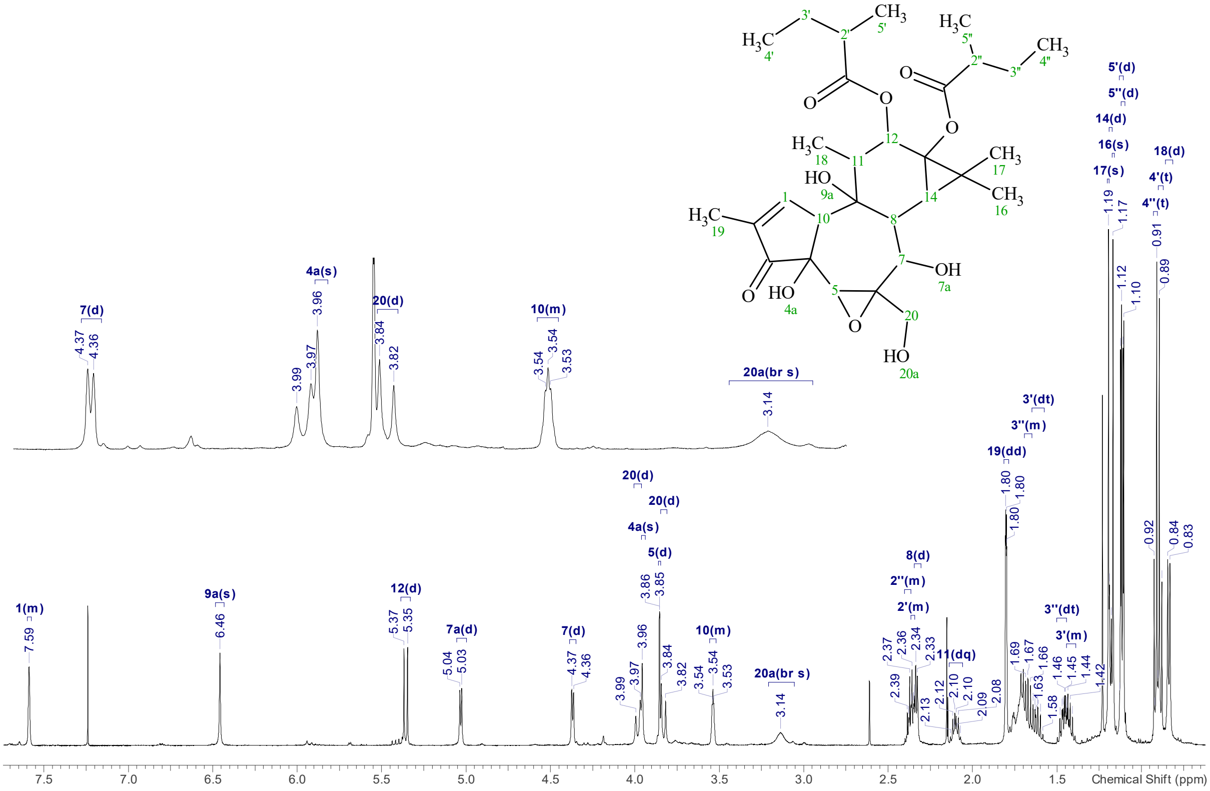

2011-02-04n3-500_001001r.esp

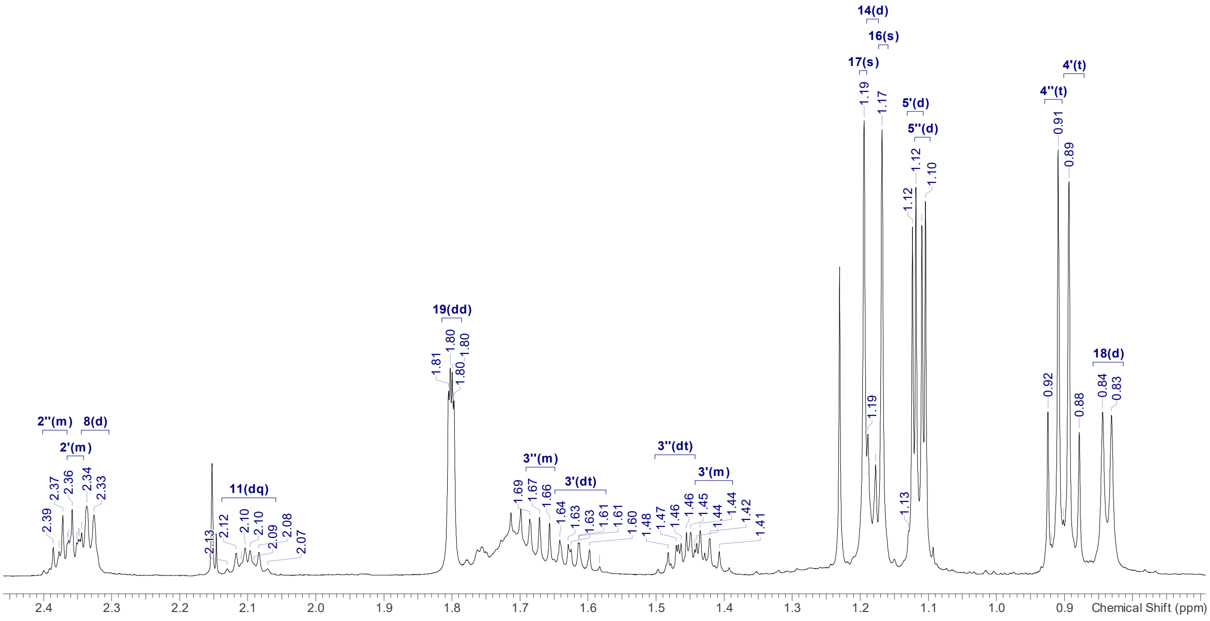

S3.32 ^{13}C NMR spectrum for EBC-161 recorded in $CDCl_3$.

2011-02-04n3-500_002001r.esp

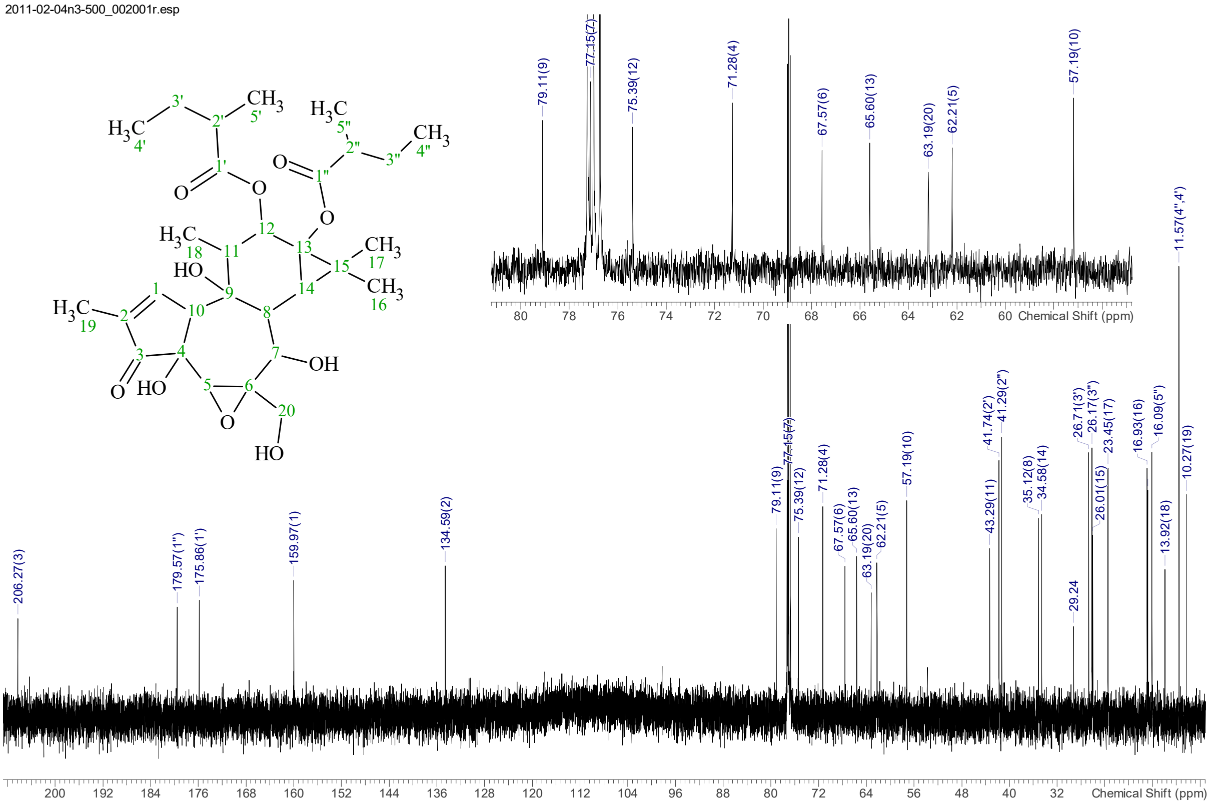

2011-02-04n3-500_002001r.esp

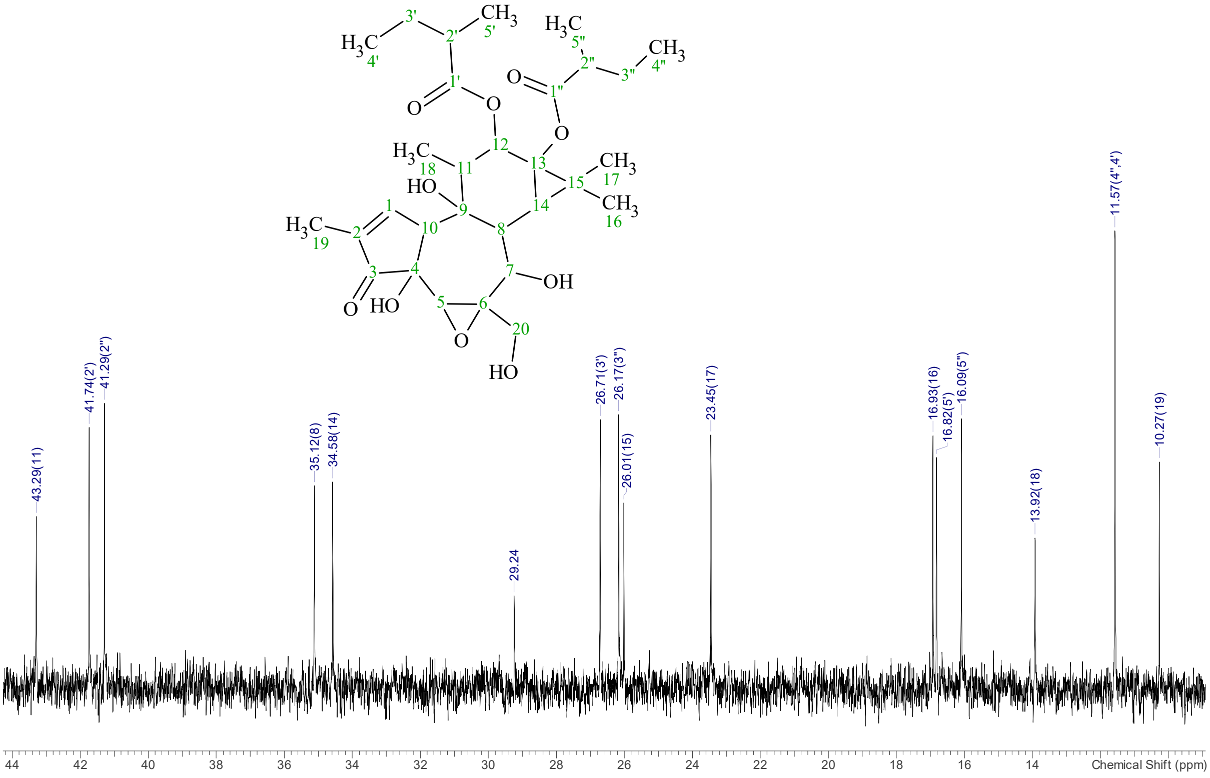

S3.33 ^{1}H NMR spectrum for EBC-167 recorded in $CDCl_3$.

2011-02-05n4-500_001001r.esp

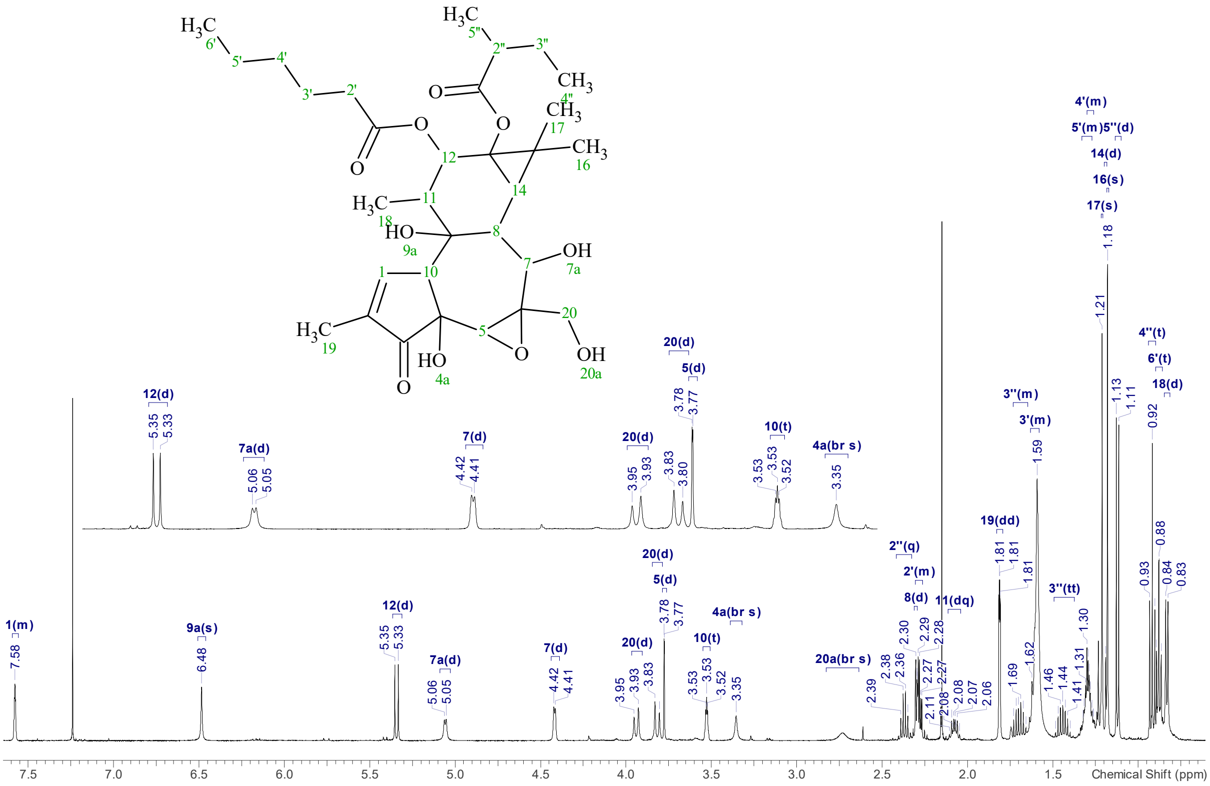

2011-02-05n4-500_001001r.esp

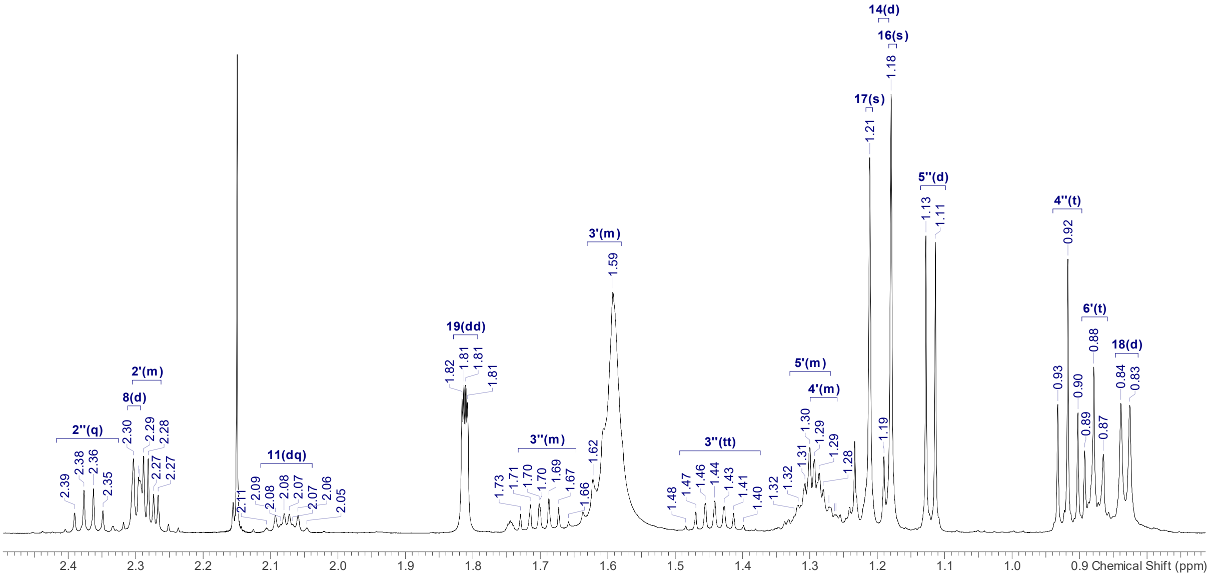

S3.34 ^{13}C NMR spectrum for EBC-167 recorded in $CDCl_3$.

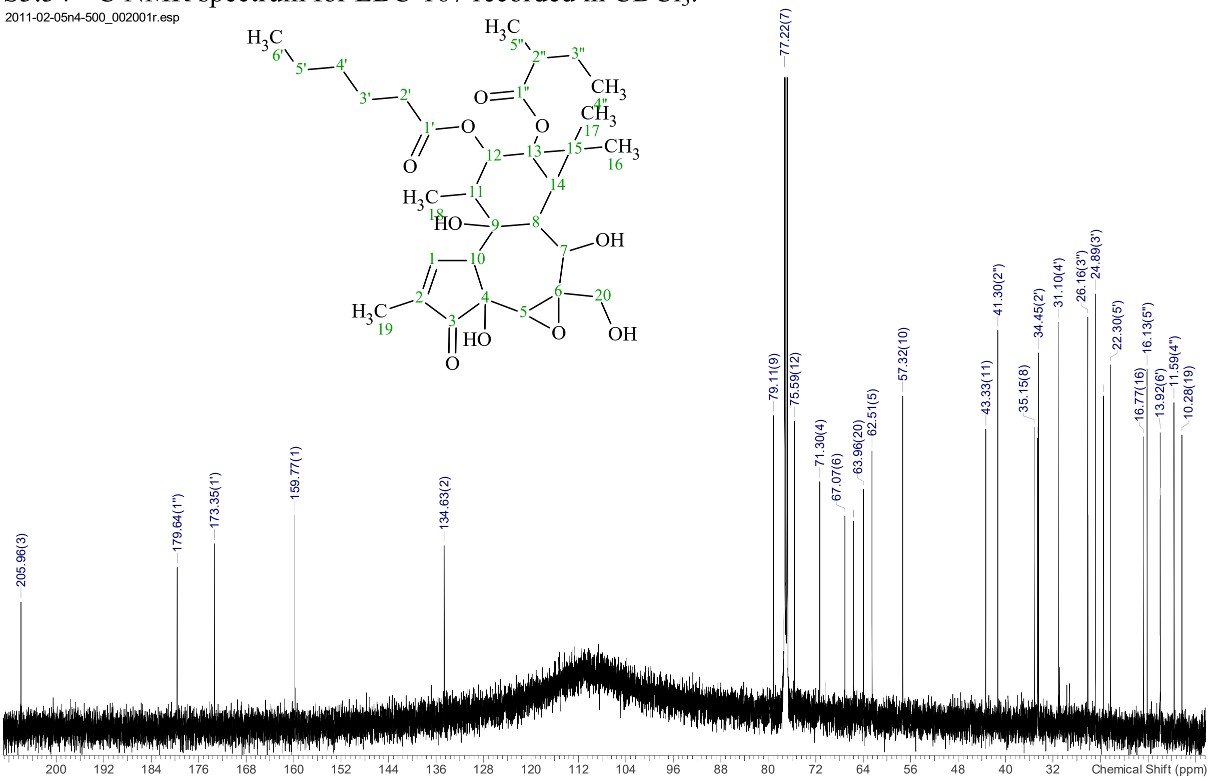

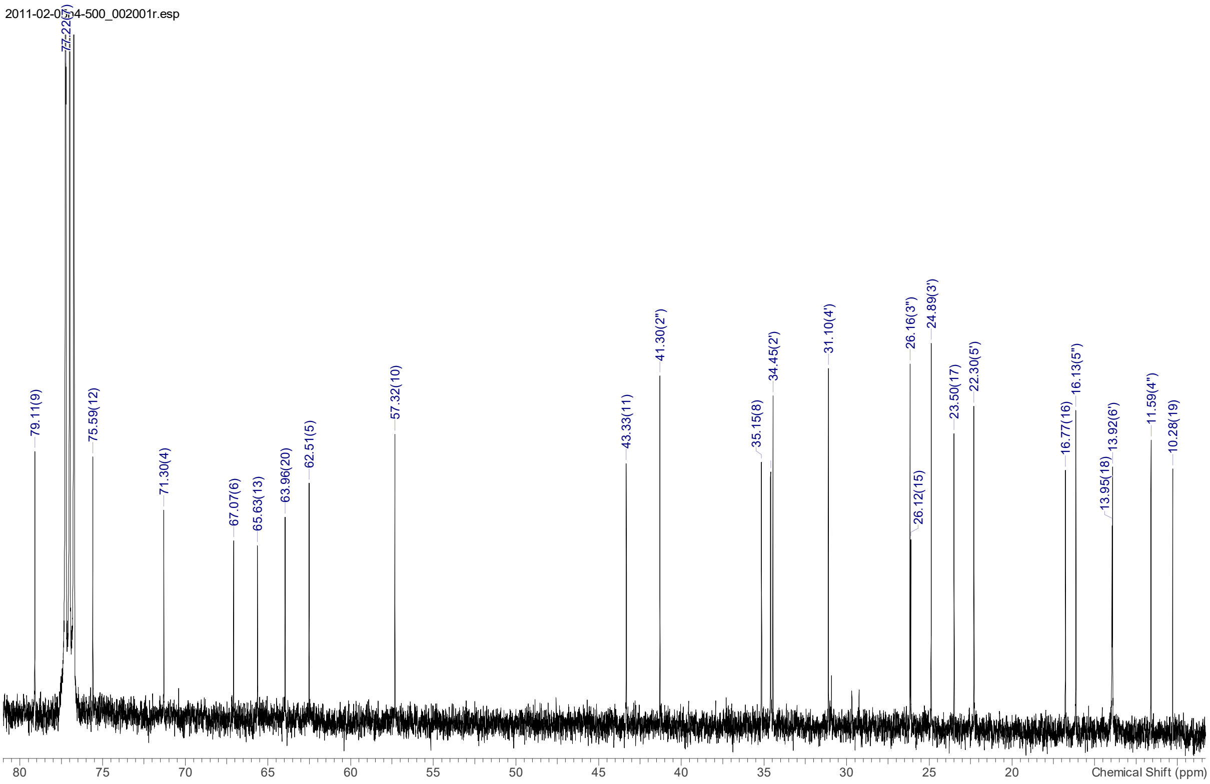

S3.35 ^{1}H NMR spectrum for EBC-170 recorded in $CDCl_3$.

2011-02-10n8-500_013001r.esp

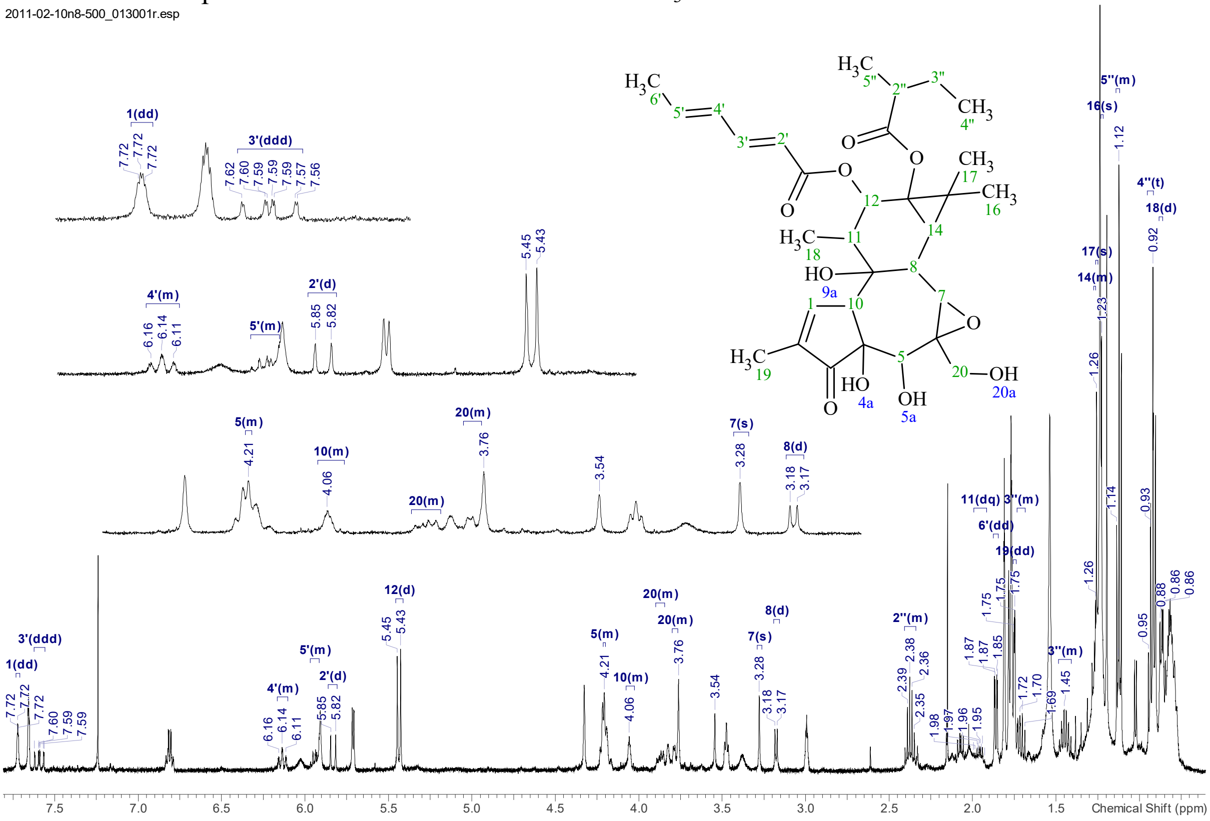

2011-02-10n8-500_013001r.esp

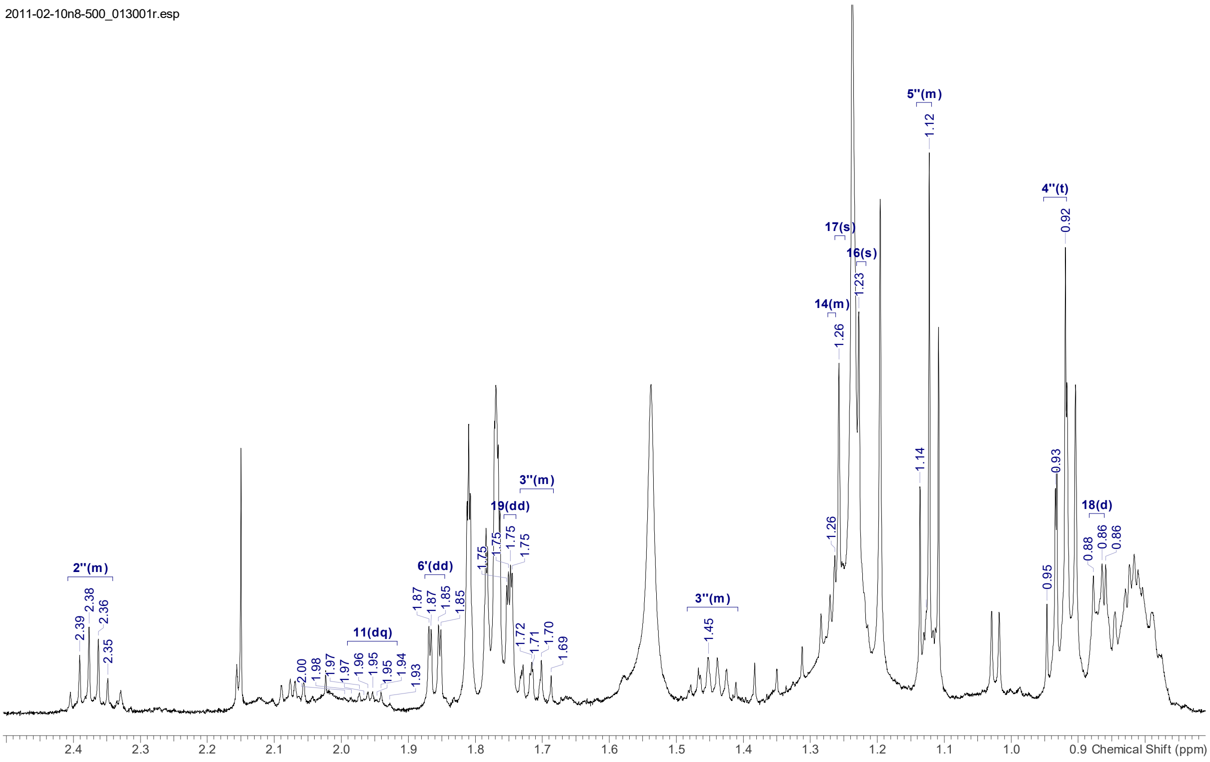

S3.36 ^{13}C NMR spectrum for EBC-170 recorded in $CDCl_3$.

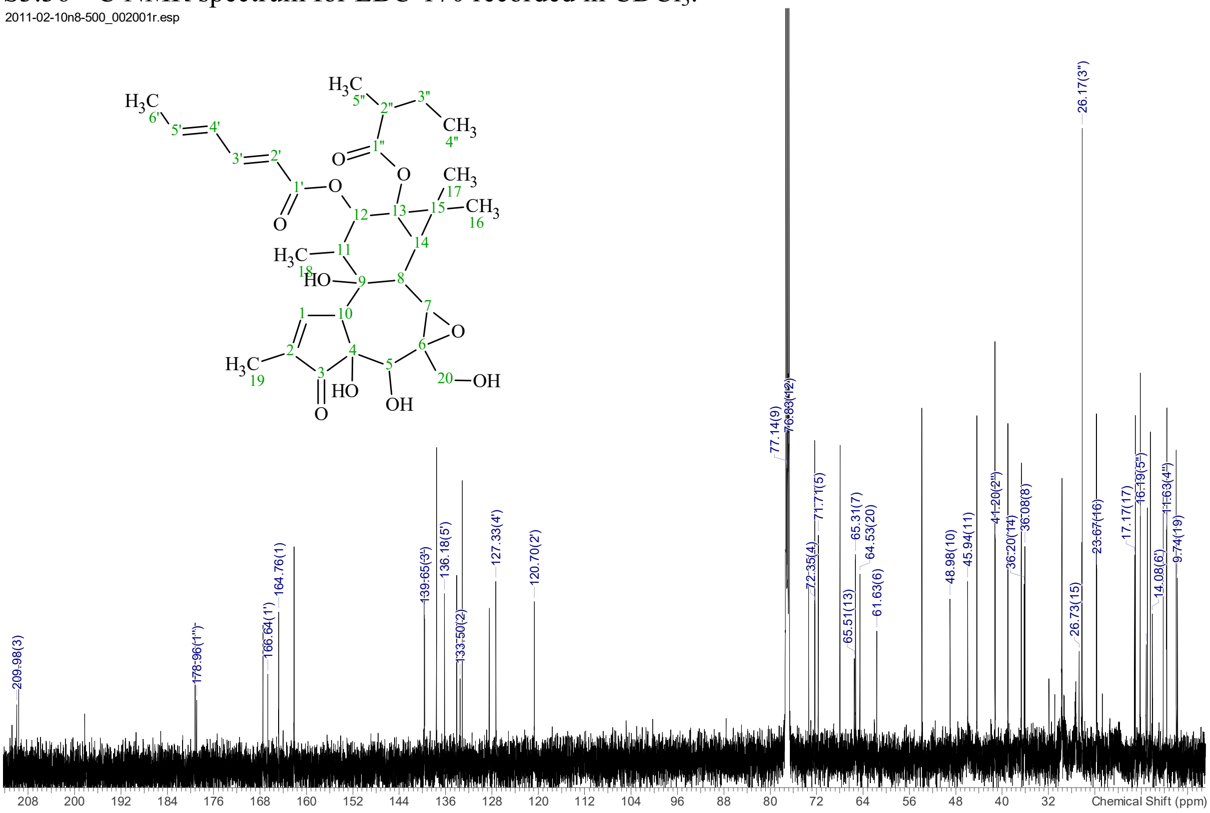

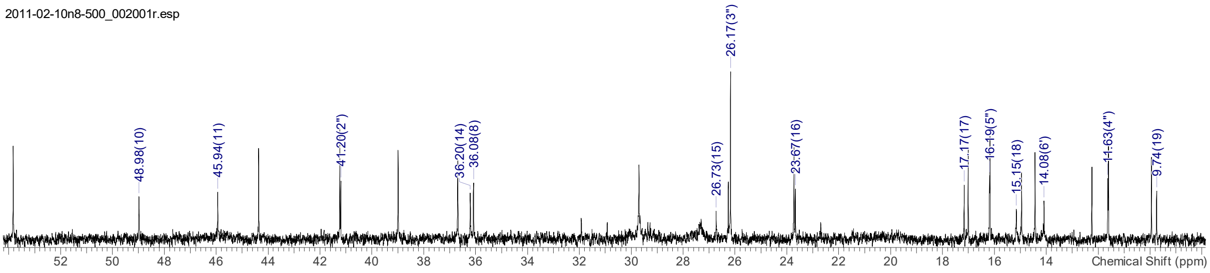

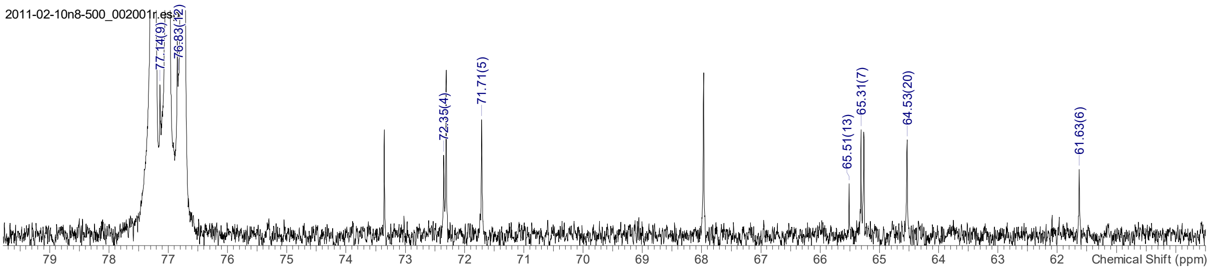

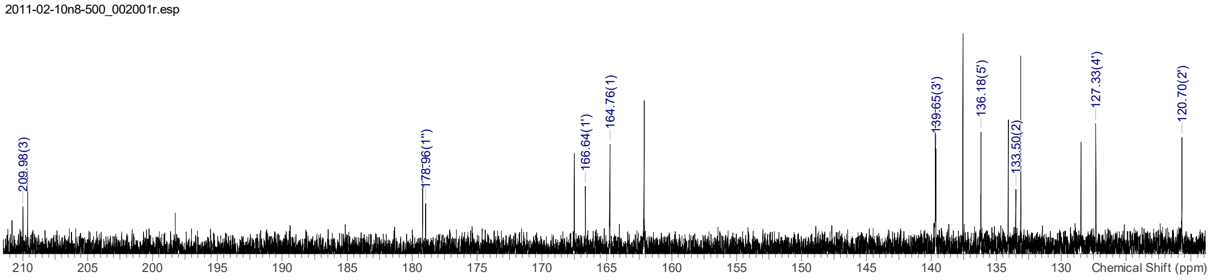

S3.37 ^{1}H NMR spectrum for EBC-172 recorded in $CDCl_3$.

2011-03-03#8-500_001001r.esp

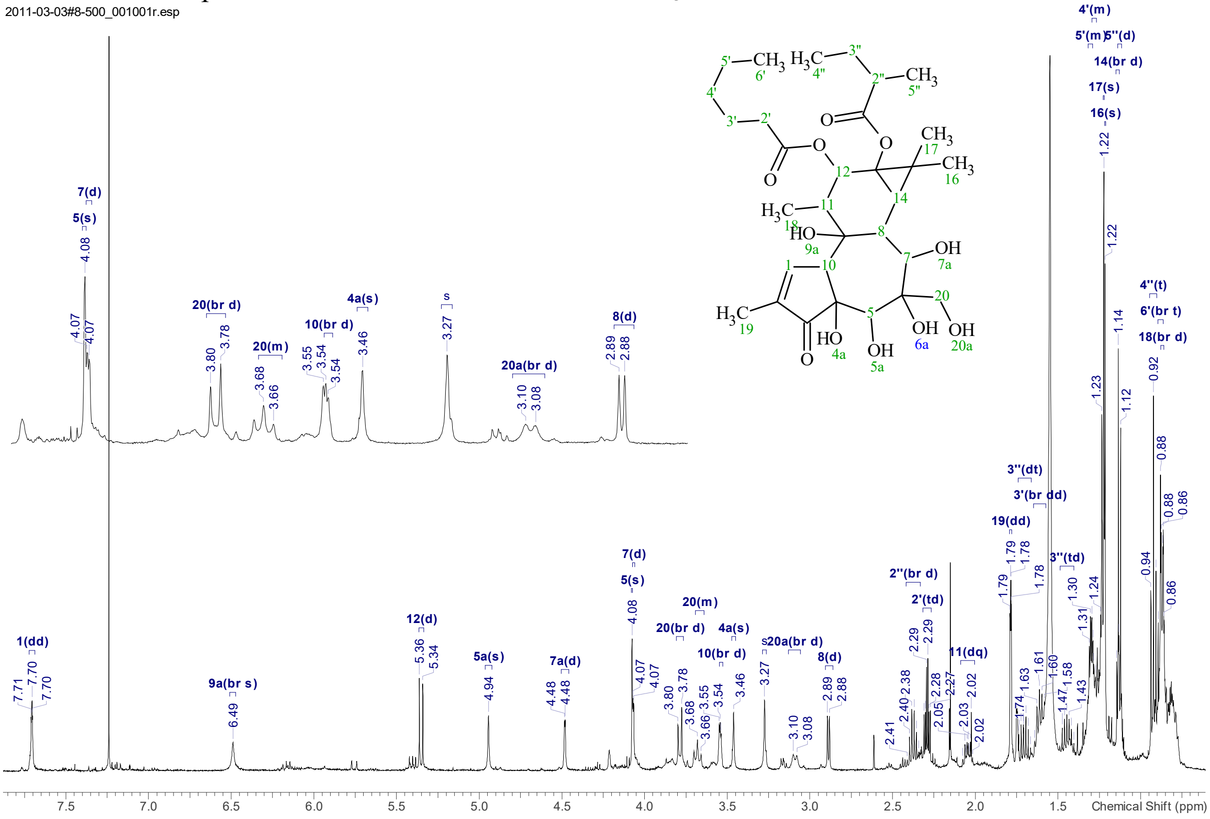

2011-03-03#8-500_001001r.esp

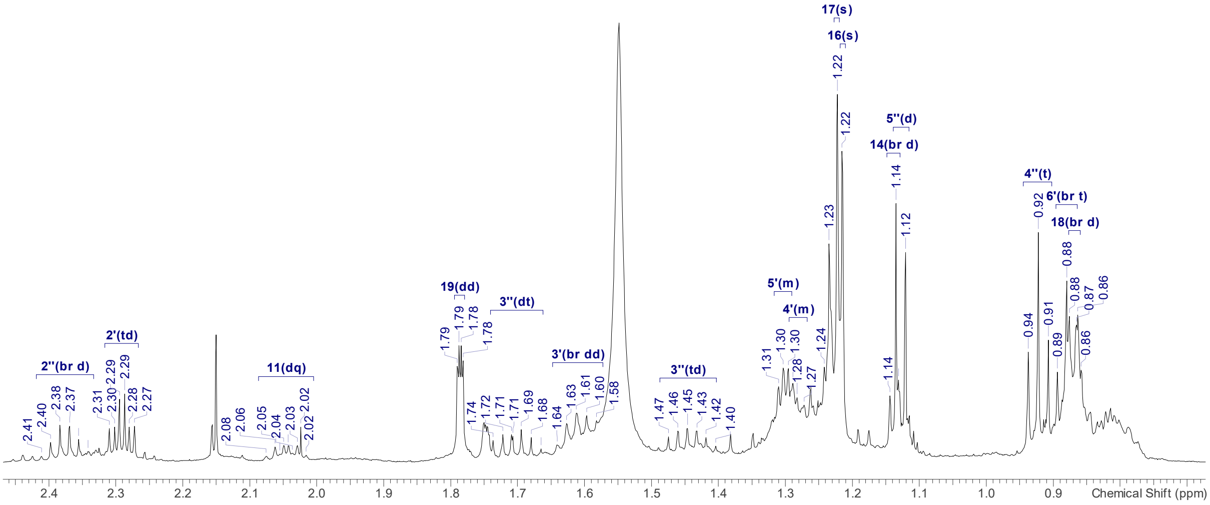

S3.38 ^{13}C NMR spectrum for EBC-172 recorded in $CDCl_3$.

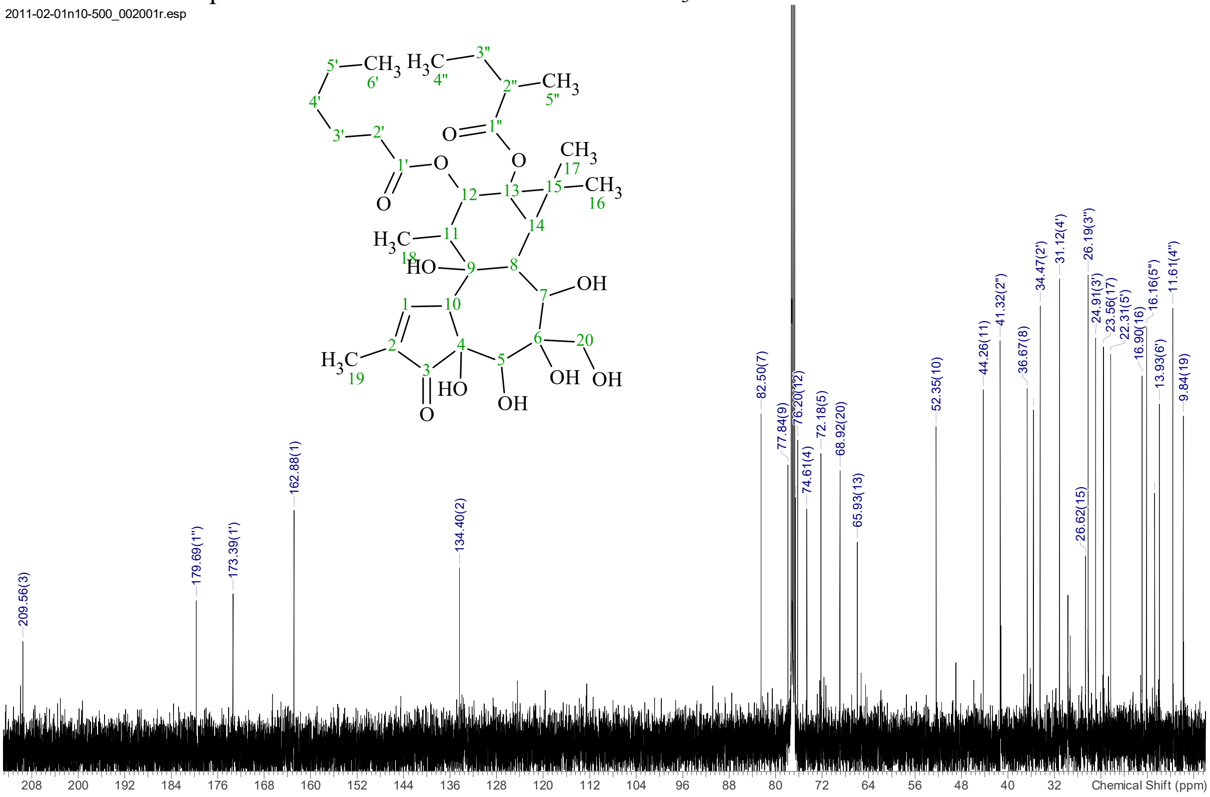

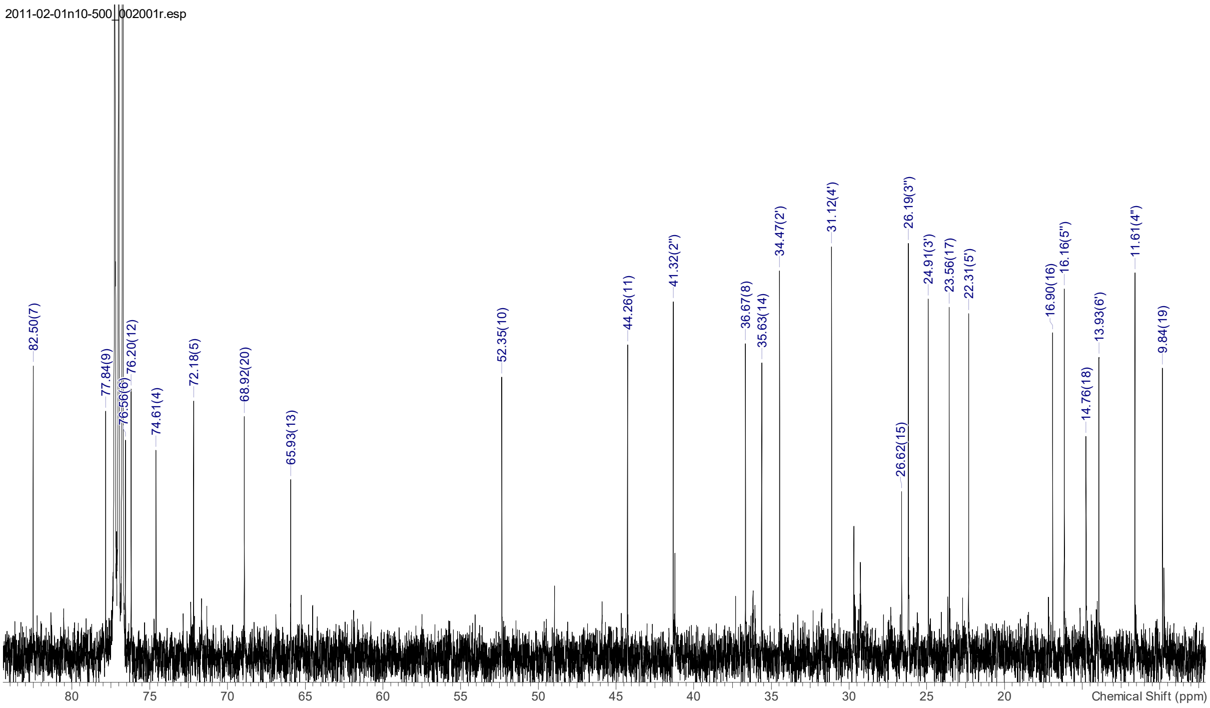

S3.39 ^{1}H NMR spectrum for EBC-177 recorded in $CDCl_3$.

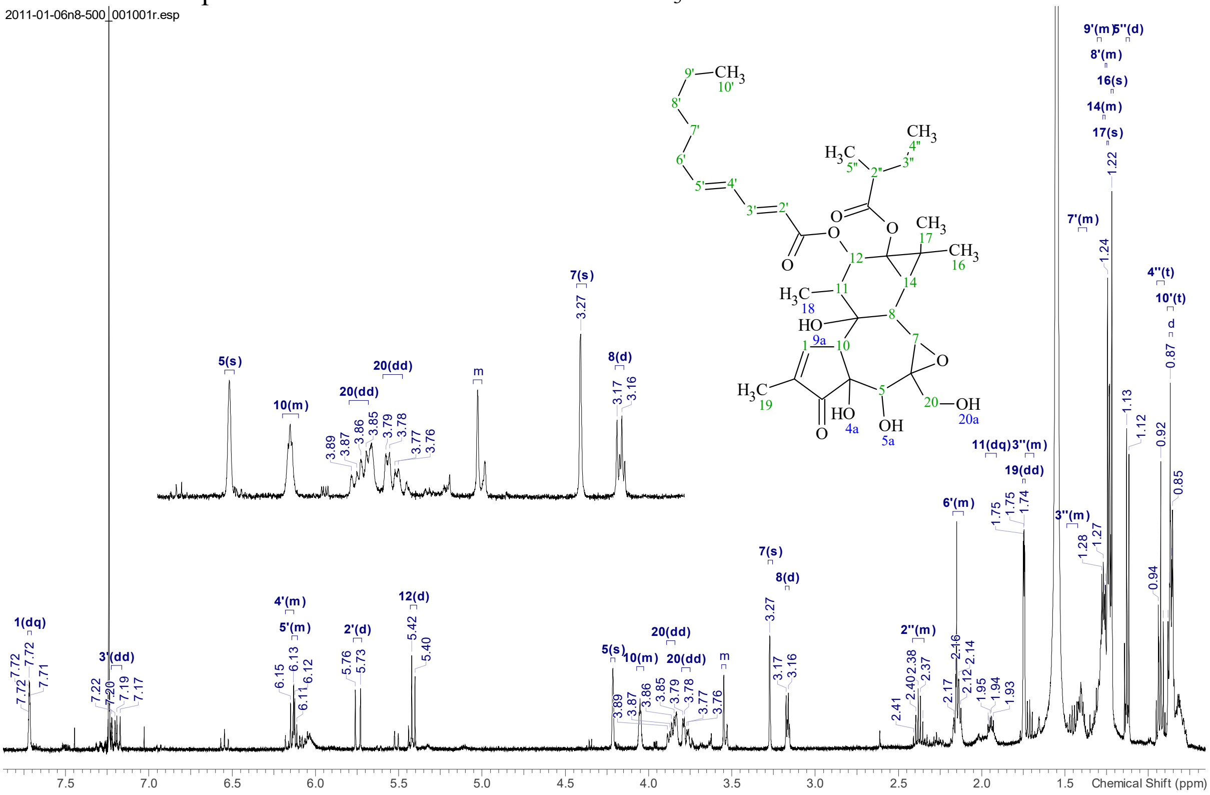

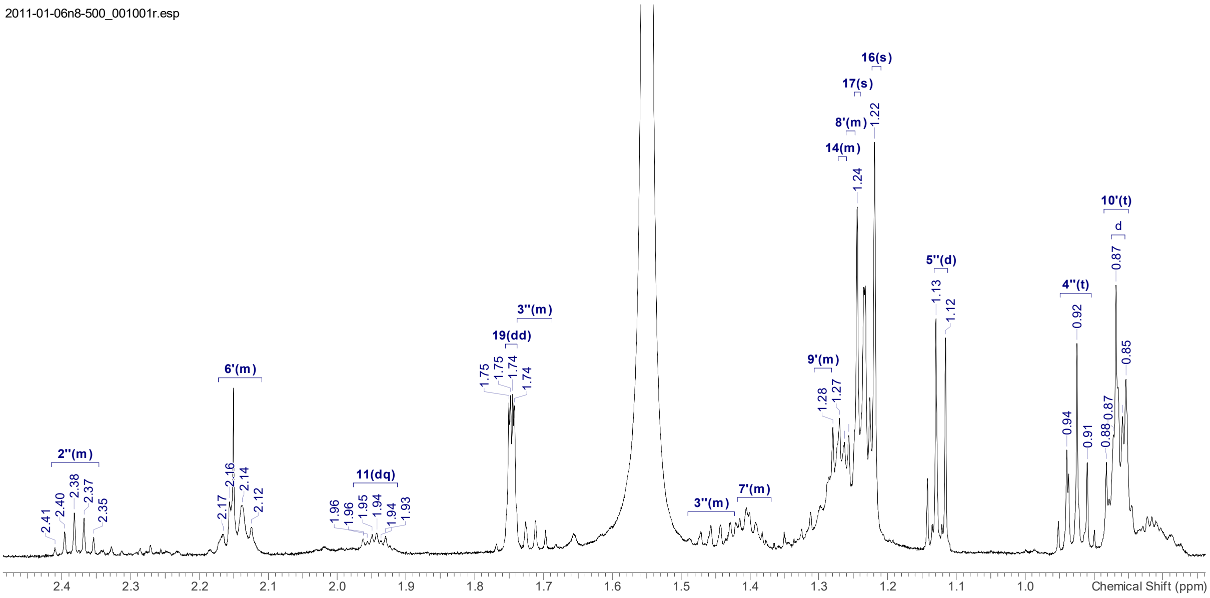

S3.40 ^{13}C NMR spectrum for EBC-177 recorded in $CDCl_3$.

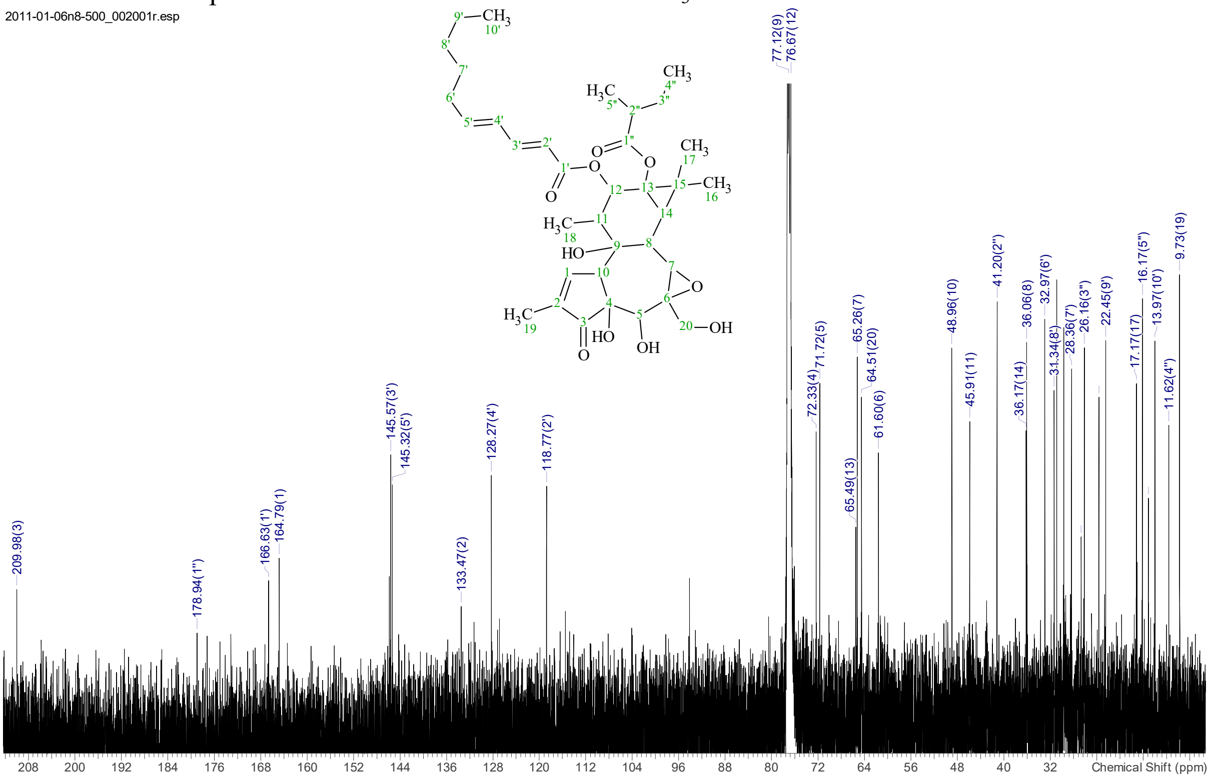

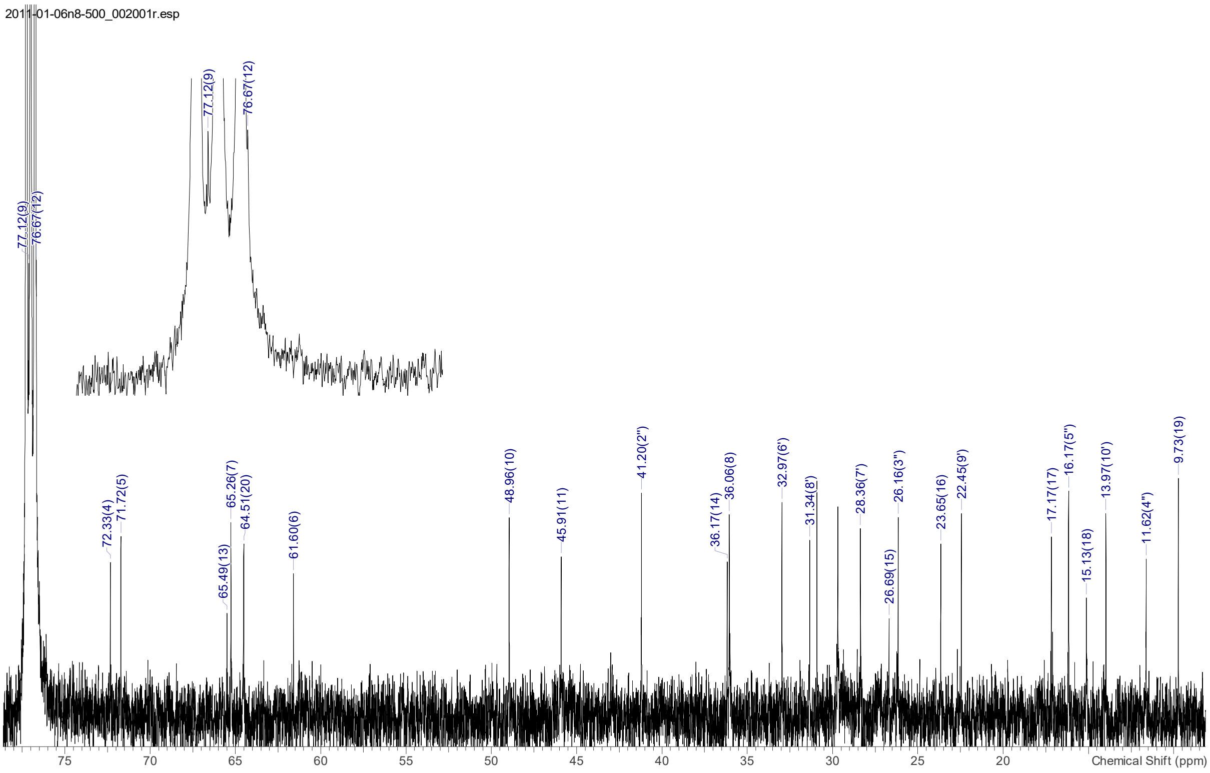

S3.41 ^{1}H NMR spectrum for EBC-186 recorded in $CDCl_3$.

2011-04-11#1-500_001001r.esp

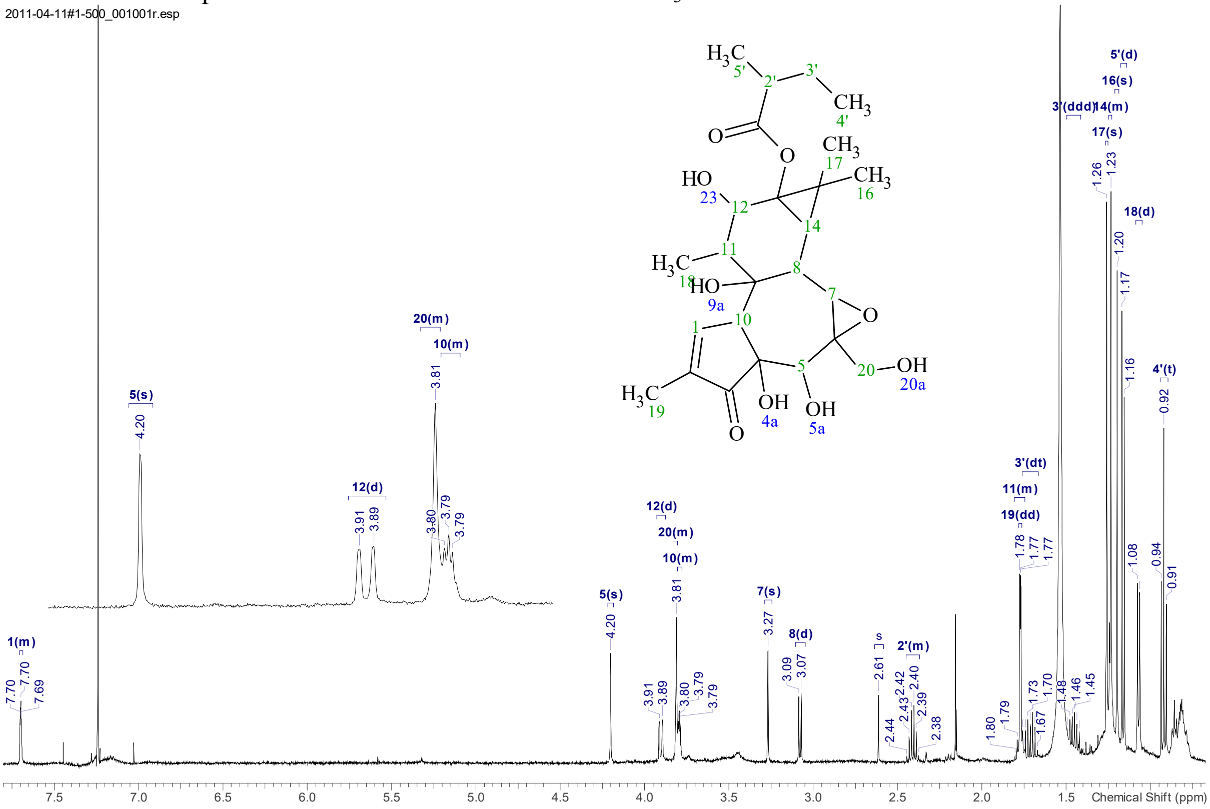

2011-04-11#1-500_001001r.esp

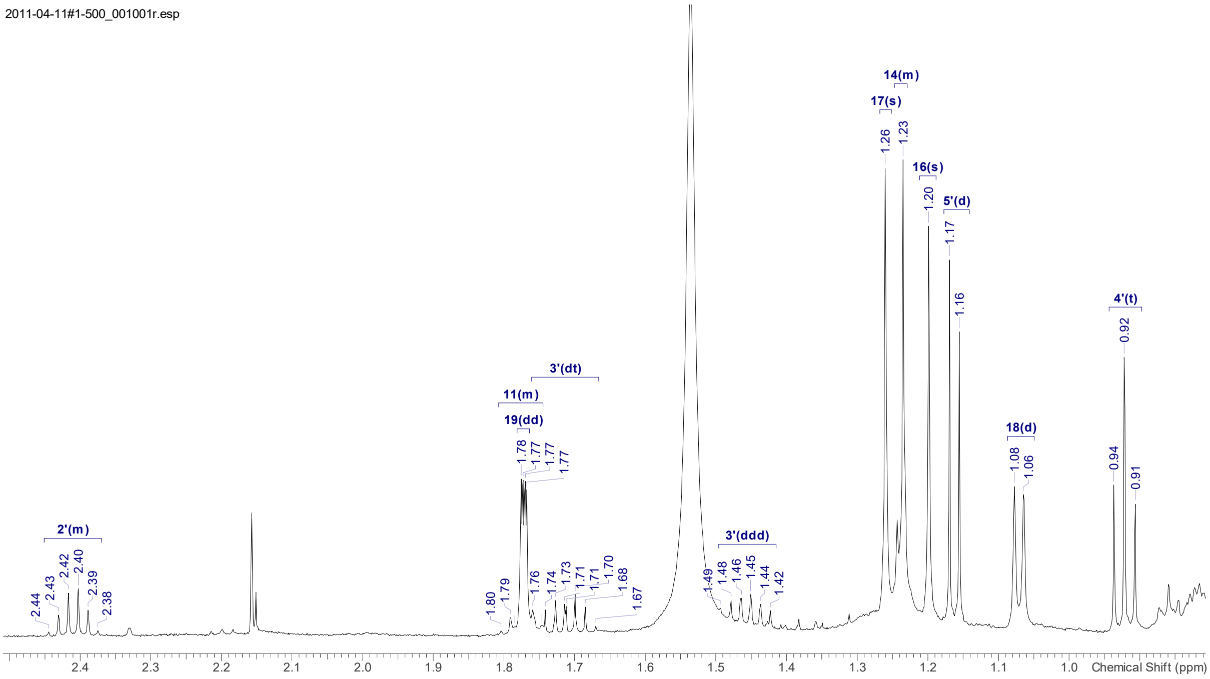

S3.42 ^{13}C NMR spectrum for EBC-186 recorded in $CDCl_3$.

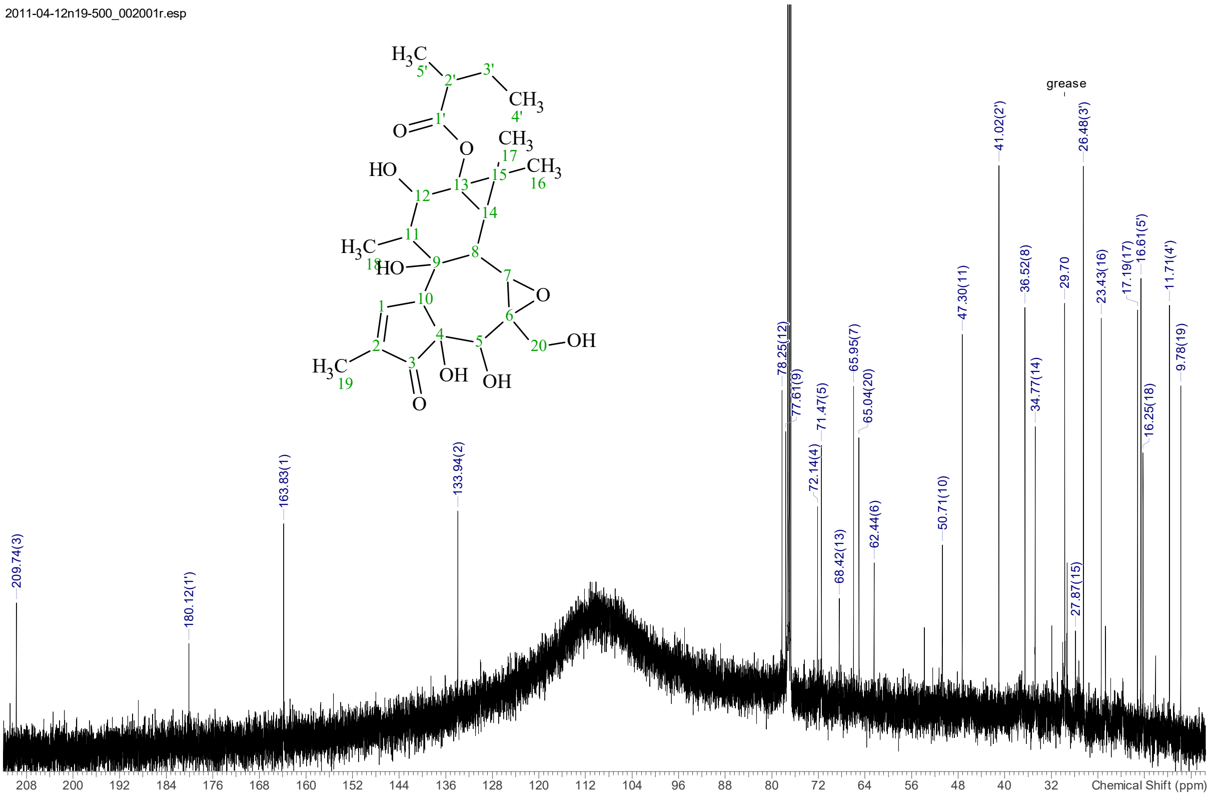

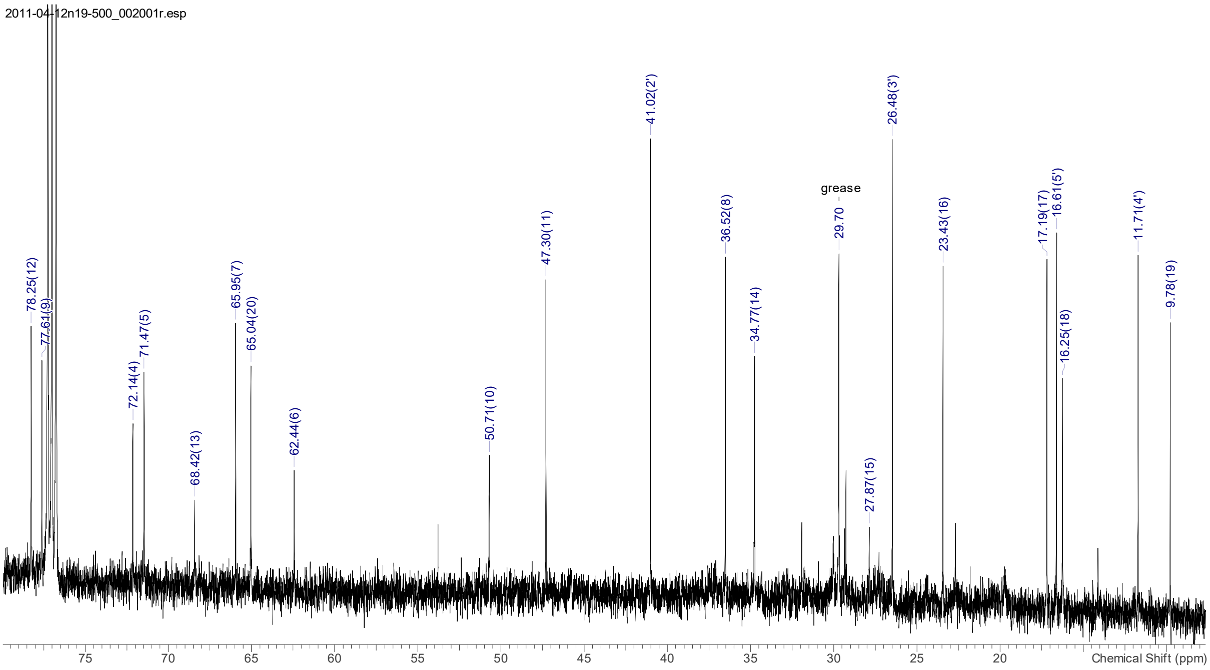

S3.43 ^{1}H NMR spectrum for EBC-188 recorded in $CDCl_3$.

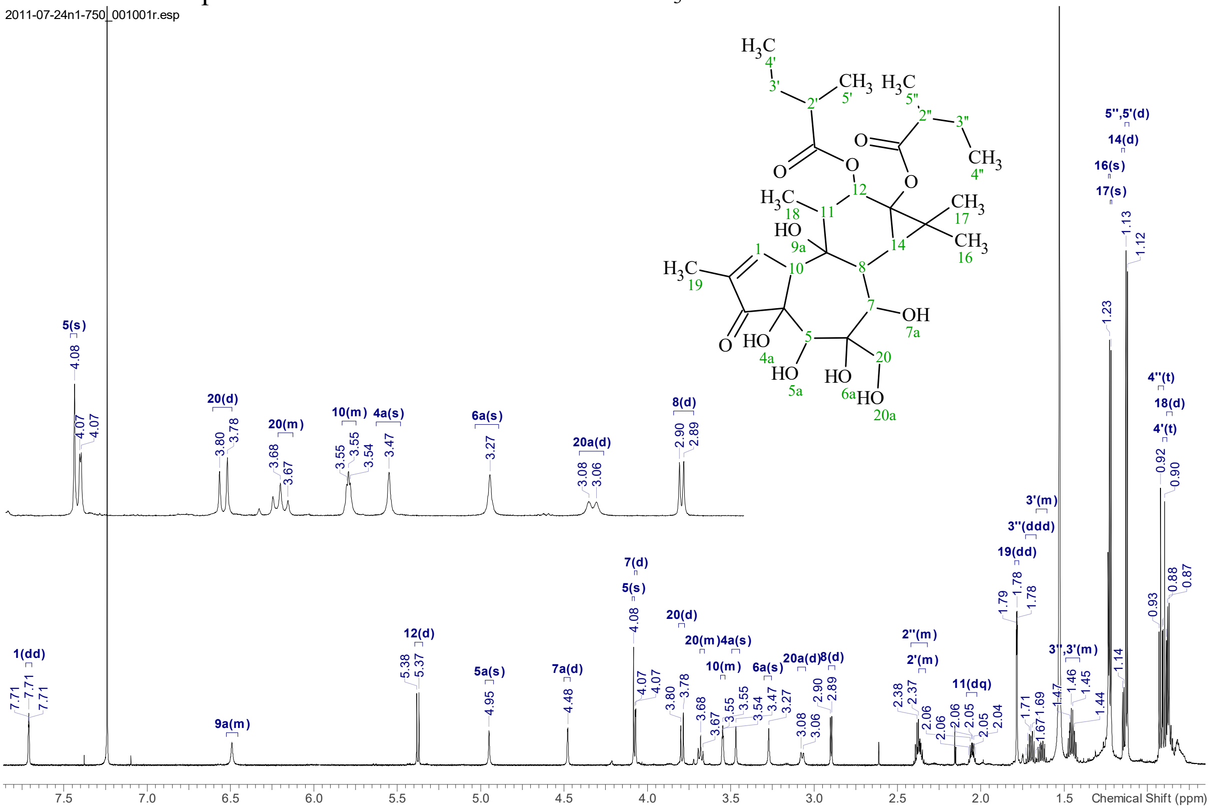

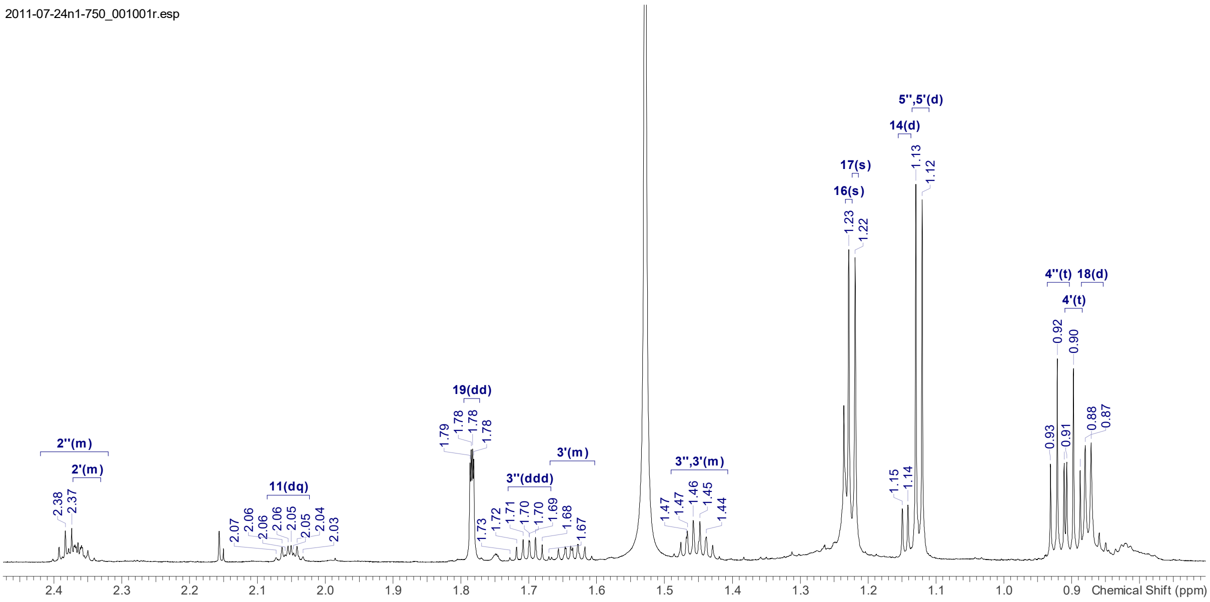

S3.44 ^{13}C NMR spectrum for EBC-188 recorded in $CDCl_3$.

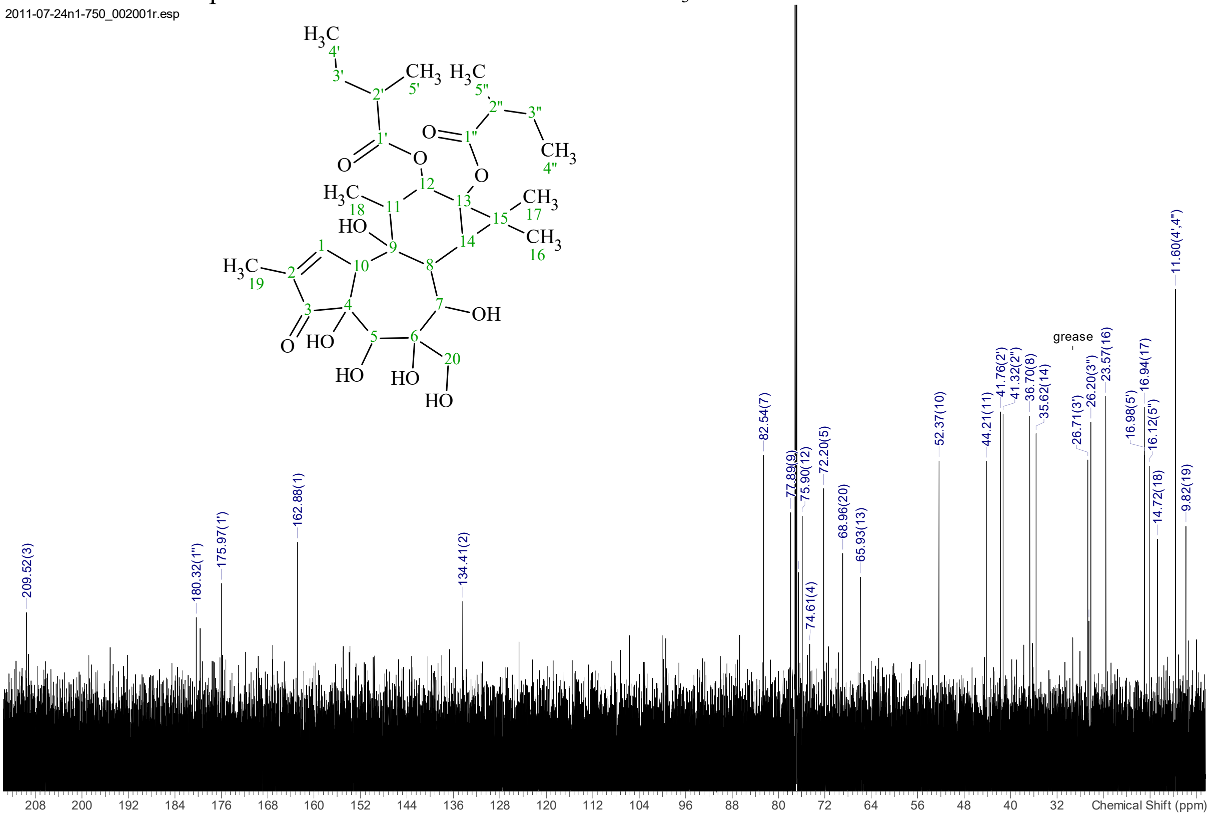

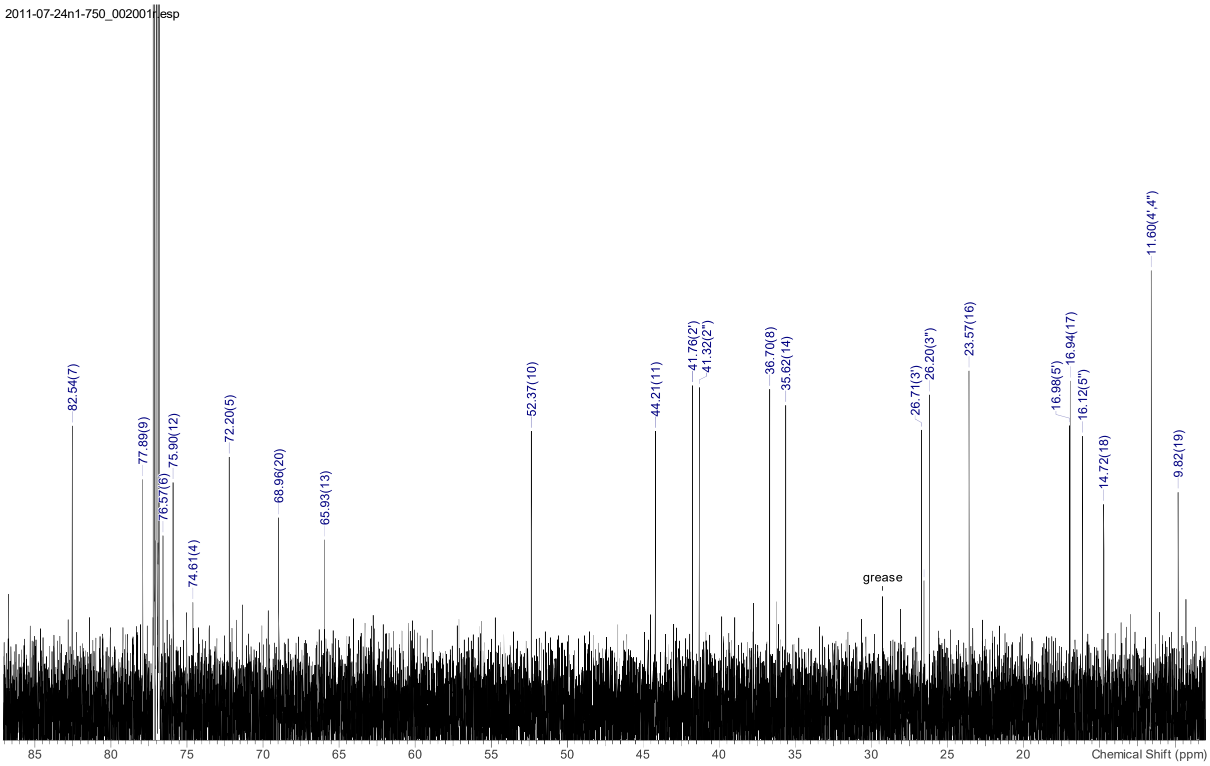

S3.45 ^{1}H NMR spectrum for EBC-211 recorded in $CDCl_3$.

2011-11-29n1-750_001001r.esp

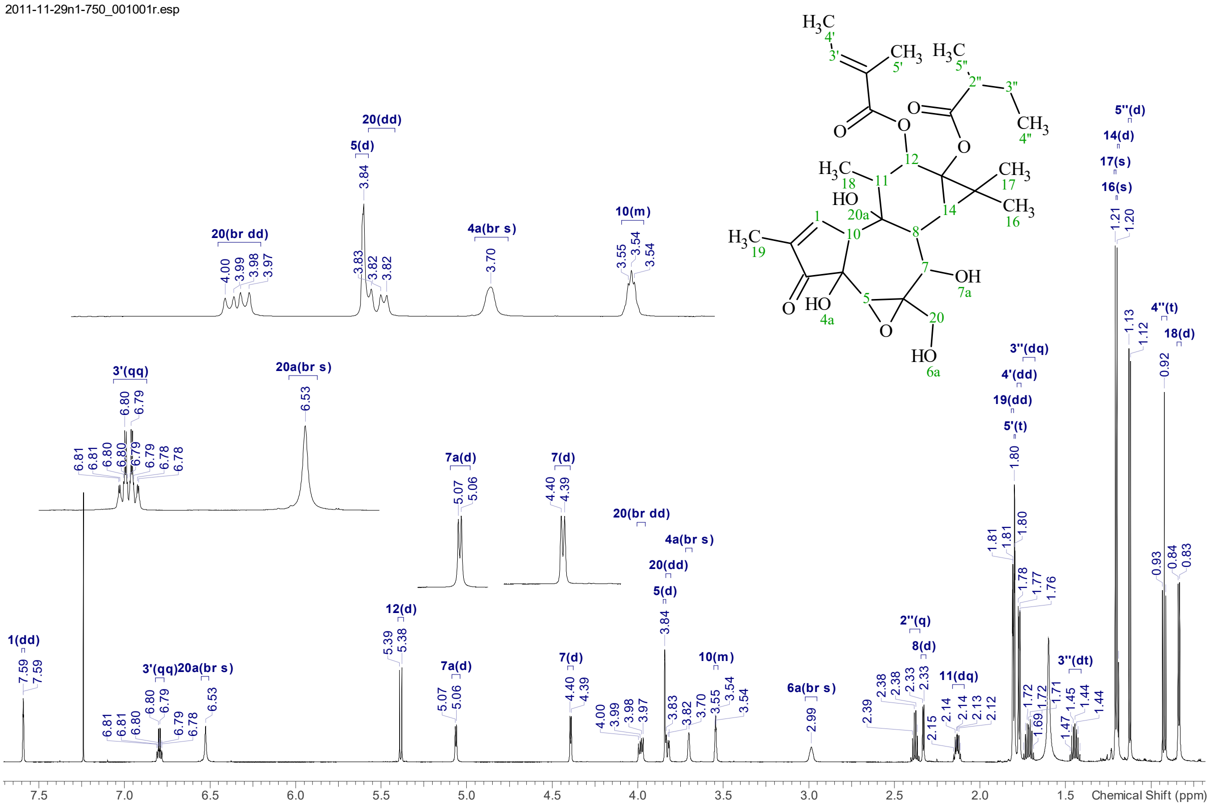

2011-11-29n1-750_001001r.esp

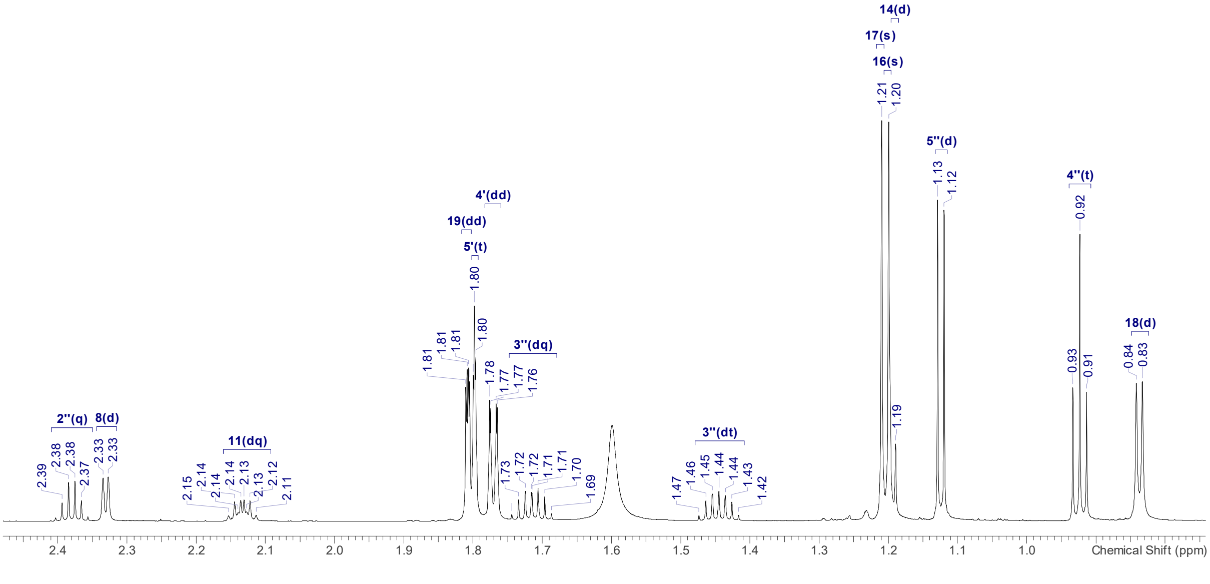

S3.46 ^{13}C NMR spectrum for EBC-211 recorded in $CDCl_3$.

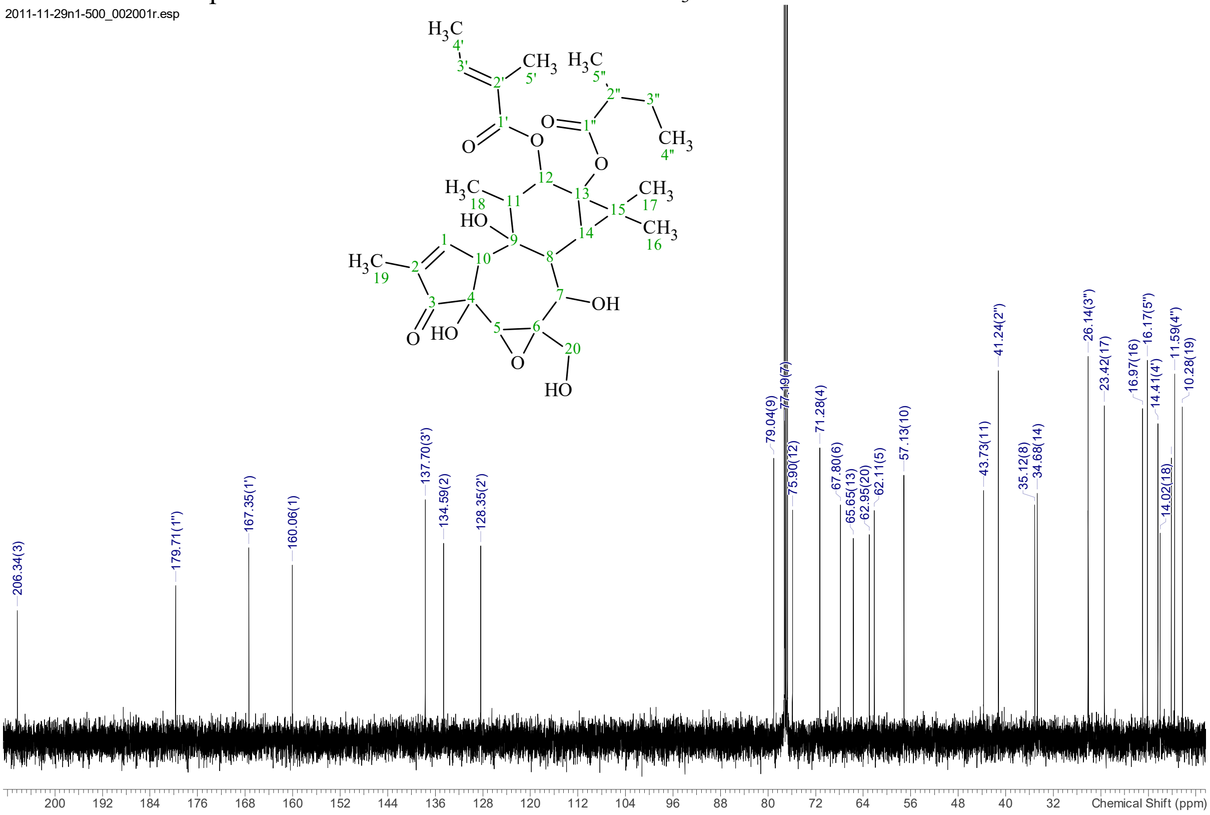

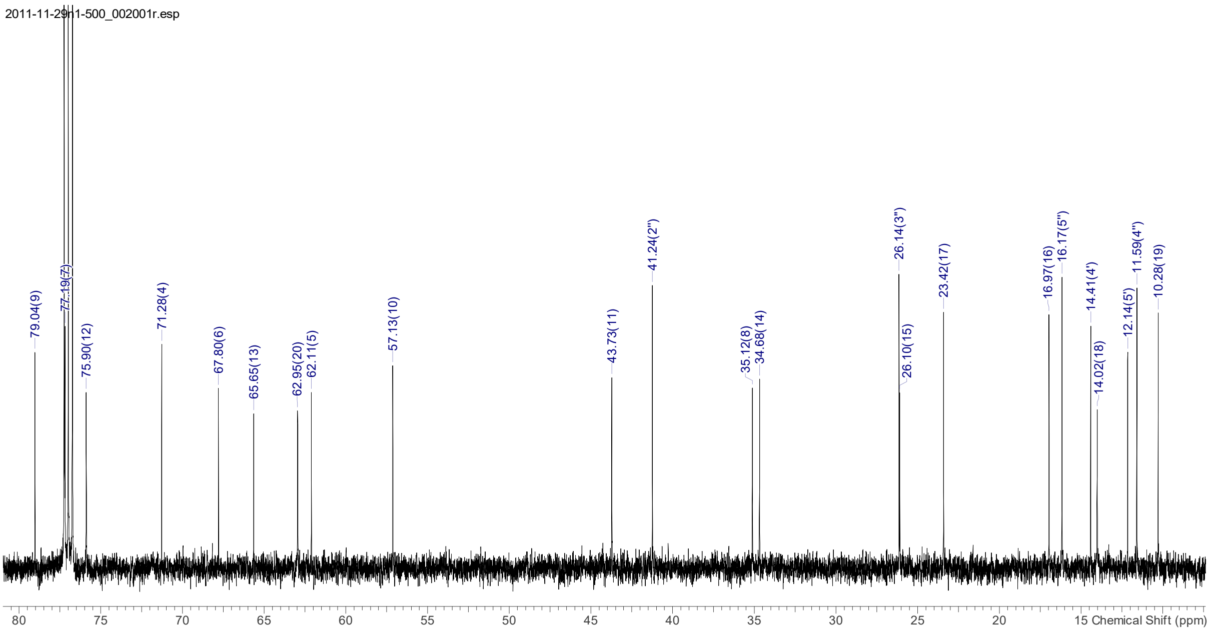

S3.47 ^{1}H NMR spectrum for EBC-344 recorded in $CDCl_3$.

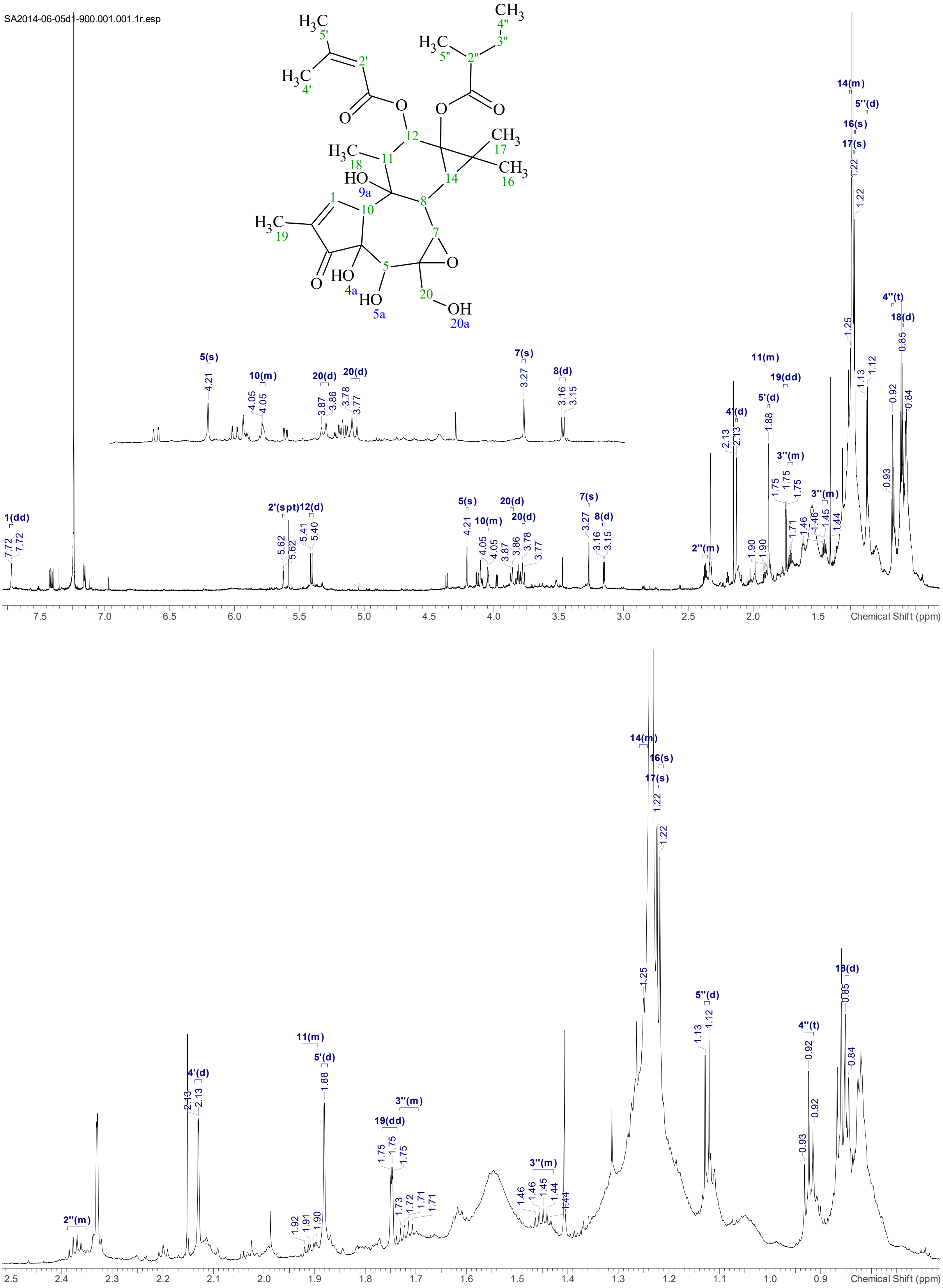

S3.48 ^{13}C NMR spectrum for EBC-344 recorded in $CDCl_3$.

SA2014-06-05d1-900.002.001.1r.esp

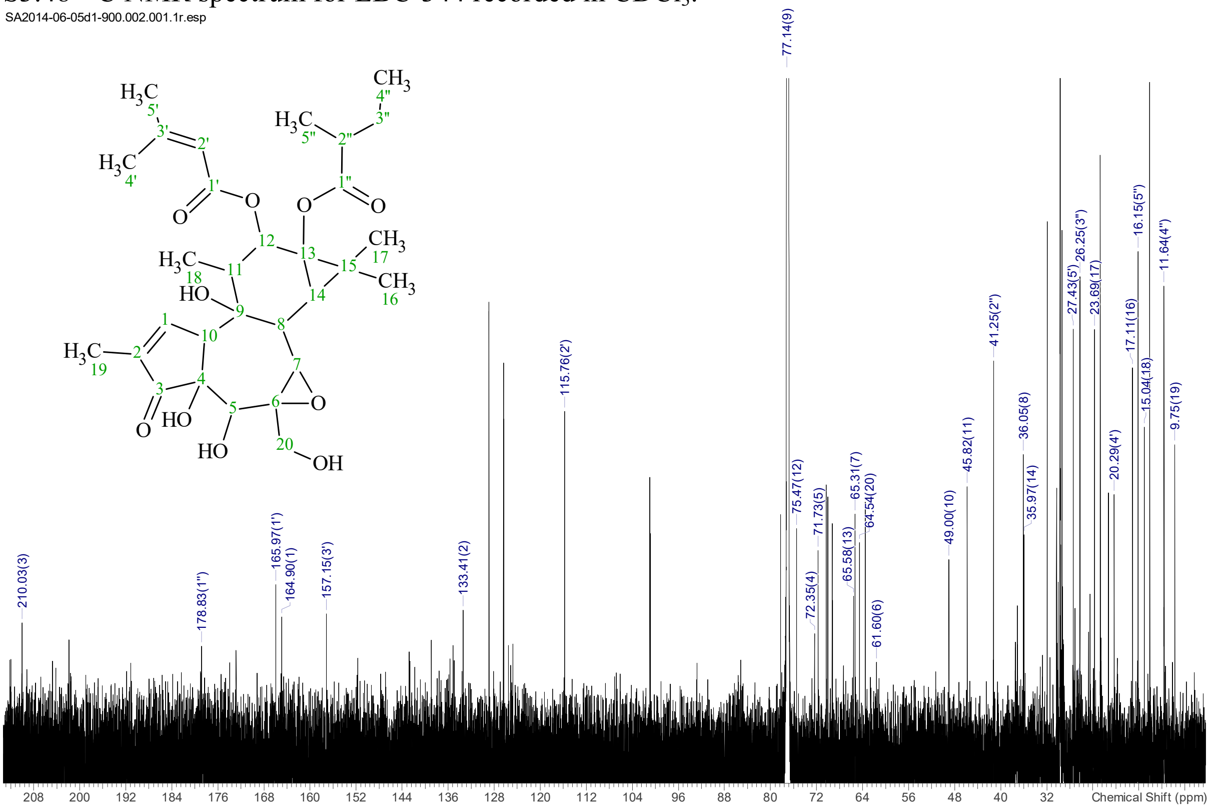

SA2014-06-05d1-900.002.001.1r.esp

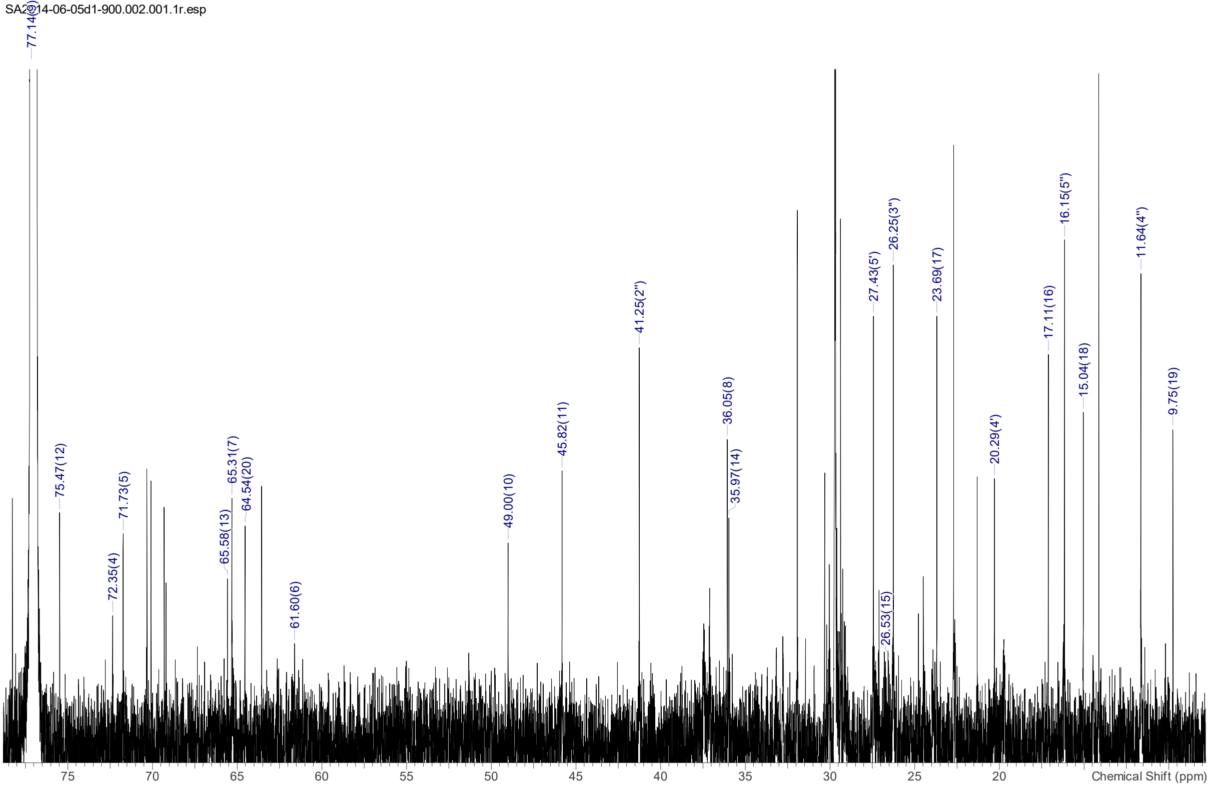

Supplement: Supplementary file 1 — Supplementary Information. [file 41598_2020_80397_MOESM1_ESM.pdf]
